# Proteo-genomics-guided interpretation of somatic mutations in cancer genomes

**DOI:** 10.1016/j.ccell.2026.07.017

**Published:** 2026-08-21

**Authors:** Do Young Hyeon, Yuxiang Zhou, Wendy K. Chung, Felix Dietlein

**Affiliations:** 1 Computational Health Informatics Program, Boston Children’s Hospital, Harvard Medical School, Boston, MA 02215, USA; 2 Department of Pediatrics, Boston Children’s Hospital, Harvard Medical School, Boston, MA 02215, USA; 3 Department of Medical Oncology, Dana-Farber Cancer Institute, Harvard Medical School, Boston, MA 02215, USA; 4 Cancer Program, Broad Institute of MIT and Harvard, Cambridge, MA 02142, USA; 5 Lead contact

## Abstract

Cancer genomes harbor hundreds of mutated genes, but their exact roles in tumorigenesis often remain incompletely understood. Here, by integrating protein structures with mutation data from 139,818 patients, we generate detailed, functional maps of mutations for 173 cancer genes in 18+ tumor types. In most cancer genes, oncogenic mutations accumulate in “significantly mutated regions” aligned with key functions, including DNA-binding domains in transcription factors (e.g., *FOXA1*), ligand-binding sites in receptors (e.g., *ERBB2*), catalytic domains in chromatin remodelers (e.g., *EP300*), substrate-recognition sites in ubiquitin-proteasome regulators (e.g., *SPOP*), and degradation signals in cell-cycle proteins (e.g., *CCND1*). In other cancer genes, mutations align more closely with constraints in three-dimensional protein structures, such as targeting the interior core (e.g., *TP53*) or a specific secondary structure (e.g., *PTEN*). Broadly, our study provides a proteo-genomic framework to characterize the effects of mutations in tumorigenesis and integrate them into workflows for clinical interpretation in precision medicine.

## In brief

Hyeon et al. integrate protein structures with population-scale cancer genomics data to characterize significantly mutated regions in 173 cancer-relevant genes. These regions align with functional domains, three-dimensional structural constraints, and gene-specific biology, providing a framework for classifying mutation patterns in cancer genes and prioritizing sparsely annotated variants for clinical interpretation.

## Graphical Abstract

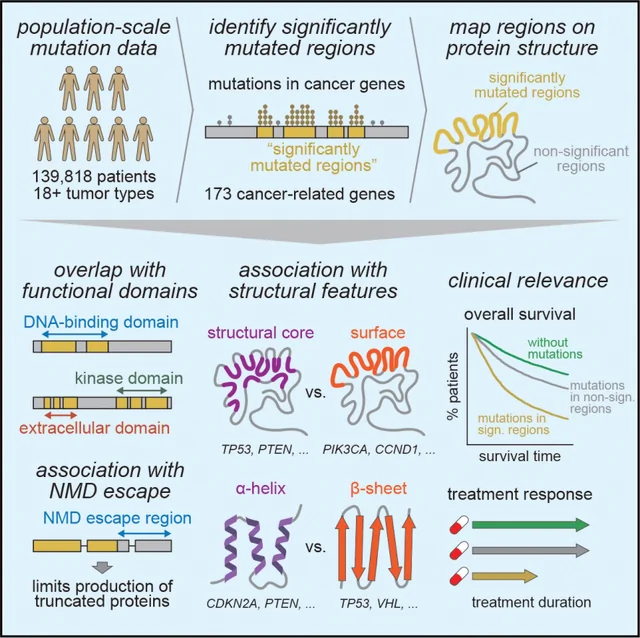

## INTRODUCTION

Large-scale consortia have sequenced thousands of cancer genomes,^1,2^ identifying hundreds of genes that are mutated during tumor development. While these studies significantly advance our understanding of the genomic landscape in almost every tumor type,^3–5^ they provide limited mechanistic insights into how mutations in cancer genes exactly contribute to tumorigenesis,^6^ often limiting their clinical interpretation in precision medicine.^7^

Most cancer genes harbor multiple domains, each executing a different function in cellular signaling.^8^ For example, receptor kinases contain extracellular domains for ligand binding, transmembrane domains for signal transduction, and kinase domains for activating downstream pathways.^9^ Functions also depend on residue positions in three-dimensional protein structures, such as stabilizing the interior core vs. mediating interactions at the surface.^10^ Mutations in the same gene can thus elicit distinct effects, depending on their functional and structural location.^11^ Therefore, we hypothesized that characterizing the spatial distribution of mutations in cancer genes—i.e., which regions, functional domains, and structural elements they preferentially target—could reveal insights into their roles in tumorigenesis.

To test this hypothesis, we uniformly analyzed the mutation patterns of 139,818 patients across more than 18 tumor types.^12,13^ We found that most cancer genes harbor “significantly mutated regions”—i.e., regions with a high density of mutations—that were linked to functional and structural features related to their roles in tumorigenesis.^14,15^ This strategy applied not only to oncogenes, where mutations often clustered in a few hotspot positions,^16^ but also to tumor suppressors, where mutations were distributed more broadly but still followed discernible spatial distribution patterns. Even for truncating mutations, often assumed to target the entire gene,^17^ we observed clear regional preferences within cancer genes, tied to functional constraints in nonsense-mediated decay.^18^ Our study thus offers a proteo-genomics framework for linking mutations to their roles in tumorigenesis, with implications for grouping patients into clinically informative subgroups in precision medicine.^19^

## RESULTS

### A proteo-genomics framework maps significantly mutated regions in cancer genes

We developed a proteo-genomics framework to dissect mutation patterns in cancer genes and link them to their roles in tumorigenesis (Figure 1A). A key component of our framework is a hidden Markov model^20^ that identifies significantly mutated regions with a high concentration of mutations in cancer genes. Critically, our model did not simply search for regions with a high number of mutations but compared patterns against several biological covariates, such as nucleotide contexts, that influence background mutation rates of individual genomic positions within cancer genes. A key advantage of this architecture is its direct interpretability, providing a direct readout of significantly mutated regions^20^ at single-base-pair resolution, without requiring prior knowledge of their location, and linking them to structural and functional features in cancer genes (Figure 1A). A detailed description of our framework, its statistics, and biological variables is provided in the STAR Methods, along with several analyses verifying its accurate calibration (Figures S1–S5).

We applied our framework to tumor sequencing data from 139,818 patients,^12,13^ after removing low-quality samples, low-confidence mutations, and potential germline variants (Figures 1A and 1B, STAR Methods). This provided a functional map of significantly mutated regions in 173 cancer-relevant genes, linking them to features in three-dimensional protein structures, functional constraints, and protein domains. Significantly mutated regions were enriched for functionally annotated residues (*p* = 2.35 × 10^−4^ for missense mutations; *p* = 3.88 × 10^−2^ for truncating mutations), supporting their biological relevance (Figures 1C, S5D, and S5E).

### Overlap of significantly mutated regions and functional domains

To characterize the biological signals captured by our framework, we first analyzed the overlap between significant regions and functional domains (Figures 1D–1F, 2A–2H, and S6A–S6D; Tables S1–S3).

#### Missense mutations in DNA-binding and regulatory domains of transcription factors

Lineage-specific transcription factors regularly harbored missense mutations in their DNA-binding domains (Figures 2A, 2H, and S6A), such as the ETS domain of *ETV6* (*p* = 1.03 × 10^−57^; chi-squared test; see STAR Methods), the zinc-finger domain of *IKZF1* (3.63 × 10^−15^), the IRF domain of *IRF4* (5.38 × 10^−32^), the Runt domain of *RUNX1* (1.37 × 10^−95^), the forkhead domain of *FOXA1* (0.29), and the HMG domain of *SOX9* (4.07 × 10^−65^). These domains bind DNA motifs to regulate cell-type-specific gene transcription and maintain cellular identity.^21^

By contrast, stress- and response-activated transcription factors were primarily mutated in their regulatory domains (Figures 2A, 2H, and S6A), including the Neh2 domain of *NFE2L2* (*p* = 1.38 × 10^−30^; activated by KEAP1), the SH2 domain of *STAT3* (2.15 × 10^−54^; dimerizes with upstream receptors), the N-terminal domain of *FOXO1* (6.14 × 10^−8^; phosphorylated by AKT1), and the MH2 domain of *SMAD2*, *SMAD3*, and *SMAD4* (*p* < 4 × 10^−6^; interacts with TGFβ receptors), whereas significant mutations were notably absent from their DNA-binding domains. Unlike lineage-specific transcription factors, these genes are not constitutively active in specific cell types but are triggered by upstream pathways in response to cellular stressors.^22^ Analogously, ligand-activated transcription factors^23^ were primarily mutated in their ligand-binding E-domains (Figures 2A, 2H, and S6A), such as nuclear receptors *AR* in prostate cancer (*p* = 1.21 × 10^−5^), *ESR1* in breast cancer (8.47 × 10^−3^), and *RXRA* in bladder cancer (0.32).

*TP53* was a notable exception, harboring missense mutations in both its DNA-binding (*p* = 1.42 × 10^−34^) and tetramerization domains (1.09 × 10^−21^) (Figure 1D). This suggested that missense mutations engaged distinct mechanisms to disrupt the function of *TP53*, depending on their domain-specific location.

#### Mutations in catalytic and substrate-recognition domains of chromatin and ubiquitin modifiers

A distinct pattern occurred in the ubiquitin-proteasome system, where missense mutations accumulated in substrate-recognition domains (Figures 2B, 2H, and S6B), such as the MATH domain of *SPOP* (*p* = 4.22 × 10^−24^), the beta-domain of *VHL* (1.46 × 10^−11^), the hydrolase domain of *BAP1* (1.02 × 10^−27^), the RING finger domain of *CBL* (1.03 × 10^−64^), and the WD40 repeats of *FBXW7* (3.01 × 10^−33^). By marking cancer genes for degradation, these domains control oncogenic pathways,^24^ including androgen signaling in prostate cancer^25^ (*SPOP*), hypoxia signaling in kidney tumors^26^ (*VHL*), MAPK signaling^27^ (*CBL*), DNA repair and apoptosis^28^ (*BAP1*), and c-Myc stability^29^ (*FBXW7*).

Similarly, chromatin modifiers displayed mutational significance in their catalytic domains (Figures 1D, 2B, 2H, and S6B), such as the MTase domain of *DNMT3A* (*p* = 5.63 × 10^−24^), the HAT domains of *CREBBP* (6.27 × 10^−121^) and *EP300* (1.48 × 10^−135^), the SET domains of *EZH2* (8.35 × 10^−8^) and *SETD2* (5.35 × 10^−97^), or the cysteine-rich domain of *TET2* (0.055). These domains transfer acetyl and methyl groups to shape epigenomic structures and control the expression of cancer-relevant genes^30^ related to PIK3CA signaling^31^ (*DNMT3A* and *EZH2*), TP53 response^32^ (*EP300* and *CREBBP*), or double-strand break repair^33^ (*SETD2*). Similarly, genes in DNA and RNA restructuring were primarily mutated in their catalytic domains, including the helicase domains of *CHD4*, *CHD5*, *SMARCA4*, and *ERCC2* (*p* < 5 × 10^−30^; nucleosome repositioning and nucleotide excision repair), the exonuclease domain of *POLE* (6.92 × 10^−94^; DNA proofreading during replication), the RNB domain of *DIS3* (3.24 × 10^−45^; degradation of abnormal RNA), and the RNase-IIIb domain of *DICER1* (3.30 × 10^−44^; cleaving of precursor microRNAs).

#### Receptors harbor significant mutations in intra- and extracellular domains

Receptor tyrosine kinases often carried mutations in multiple domains. Their intracellular mutations primarily targeted kinase domains (*EGFR* [*p* = 2.38 × 10^−6^], *ERBB2* [3.47 × 10^−4^], *FLT3* [6.68 × 10^−3^], *MET* [0.042], and *ALK* [2.28 × 10^−8^]) and transmembrane domains (*FGFR2* [*p* = 4.91 × 10^−26^], *FGFR3* [9.17 × 10^−67^], *EGFR* [1.29 × 10^−57^], *ERBB3* [5.53 × 10^−11^], *KIT* [1.03 × 10^−6^], and *RET* [1.84 × 10^−80^]), altering motifs related to kinase activity, such as ATP-binding pockets, activation loops, αC-helices, autoinhibitory phosphorylation motifs, or dimerization sites^34^ (Figures 2C, 2D, 2H, and S6C). Epidermal and fibroblast growth factor receptors carried additional mutations in their extracellular ligand-binding regions (e.g., *EGFR* and *ERBB3*), while these mutations were absent in other receptors (e.g., *FLT3*, *MET*, *KIT*, and *RET*), reflecting distinct activation mechanisms (Figures 2D, 2H, and S6C).

Several non-kinase receptor genes were mutated in similar domains, such as the intracellular ITAM signaling motif of *CD79B* (*p* = 1.87 × 10^−9^), the extracellular domain of *TNFRSF14* (1.34 × 10^−9^), and the EGF-repeat and heterodimerization domains of *NOTCH1* (1.16 × 10^−21^) (Figures 1E and 2H). Downstream signaling genes in the MAPK/ERK pathway also displayed mutations in both catalytic and regulatory domains, mirroring mutation patterns of receptor genes (Figures 2E and S6D). Mutations similarly targeted the GTPase domains of *KRAS*, *NRAS*, *HRAS*, *RAC1*, and *RHOA*; but unlike other catalytic domains, these mutations did not spread across the domain but accumulated in a few hotspots (Figure 2C).

#### Shared and tumor-type-specific mutation patterns in cancer genes

To determine whether the overlap of significant regions and functional domains was tumor type specific, we tested whether missense mutations mapped to distinct locations in different cancer types (STAR Methods).

In most cases, significantly mutated regions were either confined to a single cancer type (Figures 1B and S6E–S6G) or showed no significant differences in their location across cancer types (72.5%, 348/480 regions) (Figures S7A and S7B; Table S4), consistent with shared functional constraints. In a subset of cancer genes with cell-type-specific functions, however, we observed notable exceptions (Figures S7C–S7G). For example, *EGFR* harbored kinase-domain mutations in lung adenocarcinomas, whereas glioblastomas showed enrichment in extracellular domains (Figure S7C). Other receptor tyrosine kinases showed similar tumor-type-specific differences, including *ERBB2* and *ERBB3* (Figures S7D and S7E). Tumor-type-specific patterns were also present in *CCND1* with blood cancers enriched for mutations in the N-terminal domain and endometrial cancers in the C-terminal loop (Figure S7F). Likewise, *NOTCH1* differed in hematologic malignancies, with mutations in regions linked to receptor activation and autoinhibition, and brain tumors, with enrichment in ligand-binding and heterodimerization domains (Figure S7G).

#### Truncating mutations in regions predicted to escape nonsense-mediated decay

Truncating mutations typically followed different distribution patterns than missense mutations, even when co-occurring in the same genes (Figures 1D, 1E, and S8), and were not tied to a specific domain, apart from a few exceptions (Figures 1E and 1F). At the same time, truncating mutations were also not uniformly distributed across tumor suppressor genes but were instead often notably absent from their N- and C-terminal loops (Figure 2F). This pattern was consistent with regional constraints related to nonsense-mediated decay,^18^ a mechanism that normally targets transcripts with premature termination codons for degradation. Because truncating mutations in C-terminal regions of tumor suppressors are less likely to trigger nonsense-mediated decay, they may still produce functional proteins and thus be less advantageous to tumor cells.

Indeed, regions in tumor suppressors lacking truncating mutations closely matched those predicted to escape nonsense-mediated decay (Figures S9A and S9B; Table S5). In patients with matched expression data, truncating mutations in regions associated with nonsense-mediated decay correlated with lower expression levels, whereas those in regions escaping this mechanism did not differ in expression from non-mutated patients (Figure S9C). The size and location of regions escaping nonsense-mediated decay varied substantially between genes, depending on the size of their last exon (Figures S10A and S10B). The absence of truncating mutations in N- and C-terminal loops was particularly prominent in tumor suppressors related to genome integrity (*CDKN1A*, *CDKN1B*, *CDKN2A*, *RB1*, *STAG2*, and *TP53; p* < 1 × 10^−15^), chromatin remodeling (*BCOR*, *BCORL1*, *CREBBP*, *DNMT3A*, *EP300*, *RBM10*, and *TET2*; *p* < 3 × 10^−20^), and differentiation (*PTEN*, *RASA1*, *STK11*, and *VHL; p* < 5 × 10^−32^) (Figures 1D, 1F, and 2F), but it also occurred in many other tumor suppressors (Figure 2H; Table S2).

In many oncogenes, truncating mutations followed an opposite pattern (Figures 1E and 2G) and were enriched in their C-terminal regions, including cyclins *CCND1* (*p* = 2.82 × 10^−5^), *CCND2* (1.11 × 10^−23^), *CCND3* (3.49 × 10^−43^), and the cell-cycle regulator *PPM1D* (3.29 × 10^−81^). In these C-terminal parts, truncating mutations are less likely to trigger nonsense-mediated decay and instead remove regulatory motifs that mark proteins for post-translational degradation (e.g., PEST motifs and ubiquitination sites). This is particularly relevant for proteins that are expressed only transiently in response to stress or during certain cell-cycle phases and require precise control of their protein levels through post-translational degradation.^35^ We observed the same truncating mutation patterns in receptor oncogenes *CXCR4* (*p* = 1.90 × 10^−7^), *ERBB2* (8.65 × 10^−119^), *ERBB3* (2.33 × 10^−76^), *FGFR2* (9.84 × 10^−14^), *FGFR3* (2.65 × 10^−96^), *NOTCH1* (4.84 × 10^−14^), and *NOTCH2* (9.97 × 10^−281^) (Figures 2G and 2H). These C-terminal truncating-mutation patterns remained evident in tumor types in which microsatellite instability (MSI) was uncommon and in samples that were not classified as MSI-high, suggesting that they differed from prior reports of C-terminal frameshift indels in microsatellite-unstable tumors^36^ (Figures S10C and S10D).

#### Significant regions identify functional mutations outside canonical cancer genes and domains

We used experimental data from cell-line mutagenesis screens^37–39^ to evaluate whether our framework also captured biologically relevant signals in less-well-studied cancer genes and domains (Figures S10E–S10G).

For *POT1* and *PPM1D*, predictions from our model closely matched data from these experiments, which recorded significantly higher effects for mutations in significant vs. non-significant regions of these genes (*p* = 3.01 × 10^−3^ and 7.58 × 10^−5^; Wilcoxon rank-sum test) (Figures S10E and S10F). In *POT1*, our model identified a significant region spanning its DNA-interacting domain (*p* = 3.26 × 10^−121^), which binds and protects single-stranded telomeric ends. Concordantly, functionally relevant mutations in mutagenesis data aligned with this domain, implicating them in telomere dysfunction^40^ (Figure S10E). For *PPM1D*, our model identified a significant region in the C-terminal end, which contains the SIAH1-mediated degradation motif. Experimental data confirmed that functional mutations accumulated in this region (Figure S10F), linking them to increased PPM1D stability, an inhibitor of TP53.^41^ In *KRAS*, our model further identified a significant region outside canonical hotspots, where mutation effects have been less well characterized. Mutagenesis screening data corroborated that mutations in this region were associated with gain of function (Figure S10G), potentially altering interactions near residue L23 with the downstream effector RAF1.^42^ Additional examples of significant regions in less-established domains included the EC1 domain of *CDH1* (*p* = 4.51 × 10^−27^; cell-cell adhesion), the iSH2 domain of *PIK3R1* (1.16 × 10^−44^; PI3K regulation), and the NES domain of *XPO1* (5.66 × 10^−98^; nuclear export) (Tables S1, S2, and S3).

Together, these analyses provided a framework for grouping cancer genes according to their altered functions in tumorigenesis, capturing shared domain-level mutation architectures among functionally related cancer genes and distinguishing them from groups shaped by different constraints (Figures 2A–2G). For example, lineage-specific transcription factors were primarily mutated in DNA-binding domains, chromatin modifiers in catalytic domains, ubiquitin-proteasome regulators in substrate-recognition domains, and receptors in ligand-binding, transmembrane, kinase, or regulatory domains (Figure 2H). This region-level organization is particularly relevant because many cancer genes have multiple functions,^8^ each relevant in different biological and signaling contexts. A map of significantly mutated regions is provided in the supplemental information (Tables S1–S3) and as an interactive resource online (oncofold.org).

### Structural features align with spatial distributions of mutations in cancer genes

For most genes, significant regions did not overlap evenly within domains but only with certain subparts (Figures 3 and S11). We systematically characterized structural constraints that correlated with these patterns.

#### Mutations at residues involved in three-dimensional molecular interactions

In many genes, mutation patterns were driven by the extent to which residues mediated interactions with other molecules. For example, *FBXW7* exhibited 10 separate significant regions, covering 32% of its WD40 domain (Figure 3). Notably, all 10 significant regions corresponded to substrate-interaction sites (e.g., the degron motif of c-Myc),^43^ while the rest of the WD40 domain was not significantly mutated (*p* = 9.69 × 10^−13^; Mann-Whitney U test). Similarly, mutations in *SPOP* clustered into two distinct regions within its MATH domain, both of which participated in substrate interactions (e.g., the androgen receptor),^44^ while opposite-facing parts of the domain were not significantly mutated (*p* = 1.65 × 10^−8^). This suggested that mutations selectively targeted substrate-recognition sites within these domains, altering their ability to degrade oncogenic substrates (Figure 3). A similar, but narrower mutation pattern occurred in *KRAS*, *HRAS*, *NRAS*, and *RAC1*, where GTPase domains span almost the entire proteins, but mutations occur only in a few hotspots (Figure 2C). These hotspot positions were distinguished by their proximity to the GTP ligand (*p* < 5.22 × 10^−6^), highlighting the importance of GTP binding for their tumorigenic role^45^ (Figures 3 and S11).

Likewise, protein-ligand interactions dictated the spatial distribution of mutations in transcription factors (Figure 3). For example, mutations in *FOXA1* overlapped with its forkhead domain and accumulated in 5 distinct regions directly facing the DNA (*p* = 3.84 × 10^−9^). Other transcription factors, such as *IRF4*, exhibited similar patterns at protein-DNA interaction sites (*p* = 5.27 × 10^−5^), suggesting that mutations altered the specificity of their DNA-binding patterns.^46^

The three-dimensional geometry of mutation patterns thus reflected the size and shape of the interacting partner, with mutational imprints ranging from compact clusters at focal binding sites (e.g., GTP) to mutations spread across broader interaction interfaces (e.g., DNA) and surfaces (e.g., protein-protein interactions) (Figures 3 and S11).

#### Mutations destabilizing the interior core and secondary structures of proteins

An opposite pattern emerged in *TP53* (Figure 3), where missense mutations clustered within the interior core rather than its DNA-binding surface (*p* = 4.10 × 10^−3^). This indicated that *TP53* mutations did not alter its DNA-binding specificity but instead destabilized the structure of the domain (Figure 3), consistent with the frequent co-occurrence of truncating mutations in *TP53*^47^ (Figure 1D). Many tumor suppressors exhibited similar patterns of missense mutations accumulating in core residues (e.g., *PTEN*, *CDKN2A*, *BAP1*, and *STK11*), primarily at residues impacting protein stability (ΔΔG) (Figures 4A and S12). Furthermore, missense mutations in protein cores often accumulated within their α-helices (e.g., *CDKN2A* and *PTEN*) or β-sheets (e.g., *TP53* and *VHL*), pointing to different modes of inactivation via structural destabilization vs. loss of substrate recognition or binding of central interaction partners (Figures 4A and S13–S15).

Significant regions thus reflected an interplay between three-dimensional protein structures and domain architecture, with missense mutations concentrated at specific residues within domains (e.g., those at the surface) rather than uniformly affecting residues within domains. Notably, these associations were largely absent for truncating mutations (Figure S16), suggesting that their distribution was less affected by structural constraints.

#### Structural context distinguishes distinct modes of cancer gene (in)activation

To systematically characterize structural constraints affecting the spatial distribution of missense mutations, we integrated a unified model of secondary structures (α-helices, β-sheets, and loops), intrinsically disordered regions, residue location in the core vs. surface (solvent accessibility), effects on protein stability (ΔΔG), and the three-dimensional geometry of mutational clusters (Figures 3, S11, and S17). For each structural feature, we quantified the extent to which it overlapped with significant regions, providing a systematic, quantitative readout of spatial mutation patterns (Figures S12, S13, S16, and S18; Table S5; STAR Methods).

Reassuringly, structural features robustly distinguished oncogenes from tumor suppressors (area under the curve [AUC] = 0.856) and genes with dual roles as oncogenes and tumor suppressors (Figures 4B and S19). For missense mutations, the most discriminating features were related to protein stability (difference in Gibbs free energy [ΔΔG]; AUC = 0.833), structural context (predicted local distance difference test [pLDDT]; AUC = 0.838), and the surface vs. core (solvent accessibility; AUC = 0.782) (Figure 4B). Structural features further organized cancer genes into coherent subgroups (Figures 4C and 4D). Tumor suppressors differed primarily by the secondary structure of their missense mutations: those enriched for missense mutations in α-helices or β-sheets also had missense mutations accumulate within core residues impacting protein stability (ΔΔG), whereas those with mutations in loops tended to harbor surface-exposed missense mutations (Figure 4C). Oncogenes, by contrast, rarely showed enrichment of missense mutations in α-helices or β-sheets, but instead segregated primarily according to different three-dimensional geometries of their mutational clusters. Another group of oncogenes harbored missense mutations at surface-exposed residues or within loops in general, rather than a defined geometric pattern (Figure 4D).

In sum, these analyses provided an integrated proteo-genomic resource for distinguishing different modes of gene (in)activation, shaped by distinct combinations of structural constraints in each cancer gene (Figures 4C and 4D). In tumor suppressors, significant regions distinguished whether missense mutations primarily disrupted a specific domain; destabilized protein cores, α-helices, or β-sheets; or perturbed molecular interaction surfaces (Figure 4C). In oncogenes, they distinguished three-dimensional geometries of mutational clusters, perturbing small-molecule-binding sites, DNA interfaces, protein-protein interfaces, or broader regulatory surfaces (Figure 4D). Protein structures were important in resolving these patterns, particularly when mutations from distinct parts of the linear sequence converged on shared structural elements or three-dimensional interaction surfaces.

### Significant regions are enriched for literature-curated oncogenic mutations

To verify the biological accuracy of the significant regions identified by our framework, we evaluated their enrichment for oncogenic mutations annotated in the literature. As references, we used the COSMIC Mutation Census,^48^ CIViC,^49^ and ClinVar^50^ (cancer-specific somatic mutations only), which curate oncogenic mutations based on reported tumorigenic roles and clinical relevance.

Overall, literature-curated oncogenic mutations were strongly enriched in significant relative to non-significant regions within the same cancer genes (Figures 5A and S20A–S20C; Table S6). This enrichment was consistent across COSMIC (AUC = 0.82; *p* < 3.86 × 10^−20^; DeLong’s test), CIViC (AUC = 0.81; *p* < 3.30 × 10^−1^), and ClinVar (AUC = 0.85; *p* < 1.24 × 10^−3^), despite the different curation strategies of these resources (Figure 5A). For example, the top 10% of significantly mutated residues contained 62.4%, 57.7%, and 70.9% of the oncogenic mutations from COSMIC, CIViC, and ClinVar (Figure 5A).

We repeated the same analyses for pathogenicity scores^15,51–53^ (AlphaMissense, CADD, phyloP, and FoldX) and driver identification methods^54–56^ (Hotspot3D, OncodriveCLUSTL, and ActiveDriver). As expected, they also showed enrichment for annotations from COSMIC, CIViC, and ClinVar. However, their enrichment was less pronounced and more variable across individual genes than that of significant regions from our model (Figures 5A and S20A–S20C).

Notably, these approaches are designed to detect distinct biological signals. Pathogenicity scores identify mutations of general functional importance, regardless of a specific disease context. For example, in *KRAS*, they assigned high scores across much of the GTPase domain, reflecting its central role in KRAS function (Figures 5B and 5C)—whereas in cancer, oncogenic mutations were concentrated at a few hotspots in *KRAS* and exhibited limited overlap with pathogenicity scores. In *TP53*, by contrast, oncogenic mutations are distributed more broadly (Figures 5B and 5C). Driver identification methods that search for recurrent or spatially clustered signals did not recover non-recurrent oncogenic mutations annotated in COSMIC for *TP53* (Figures 5B, 5C, and S20D). Together, our framework thus captured a cancer-specific signal that was well aligned with knowledge bases and added to that provided by pathogenicity scores and identification methods.

### Significant regions localize mutations of biological and clinical relevance

To further define the biological relevance of significant regions, we analyzed matched clinical, expression, and multi-omics data. Across these orthogonal data types, mutations in significant regions showed several hallmarks of oncogenic events (Figures 6A–6D; Table S7): they were associated with stronger changes in gene expression (*p* = 3.18 × 10^−7^), transcription factor activity (*p* = 8.18 × 10^−148^), and downstream signaling (*p* = 1.58 × 10^−2^) (Figures 6A–6C; S21A–S21D; Table S8). They also tended to occur earlier during tumor evolution (*p* = 3.53 × 10^−21^) (Figures 6D S21E, and S21F). For example, in *TP53*, missense mutations in significant regions showed different *TP53* expression (*p* = 3.57 × 10^−8^) and transcriptional activity (*p* = 1.50 × 10^−162^) than non-mutated tumors, whereas tumors with mutations in non-significant regions did not show these changes and largely resembled the baseline of non-mutated tumors (Figure S22). Similar differences were observed across many other cancer genes, tumor types, and datasets (Figures S22 and S23).

Mutations in significant vs. non-significant regions were also associated with different clinical survival (*p* = 3.18 × 10^−3^) and therapy response (*p* = 5.90 × 10^−2^) (Figures 6E and S24; Tables S8 and S9). Mutations in highly significant regions were further linked to known resistance phenotypes, such as doxorubicin in breast cancer (*TP53*; adjusted *p* = 4.5 × 10^−3^) or fluorouracil and leucovorin in colorectal cancer (*KRAS*; adjusted *p* = 1.16 × 10^−2^), compared with non-mutated patients, whereas less-significant mutations in the same genes and cancer types were not associated with these effects after accounting for tumor stage, hormone receptor status, subtype, and clinical variables (Figures 6F and S25; Table S10). While some differences were only borderline significant and larger treatment-specific cohorts would be needed to confirm them, they suggested that the location of mutations informed their associations with clinical phenotypes.

In sum, significant regions captured functionally and clinically relevant parts of cancer genes. Our framework derived this biological signal from population-scale sequencing data without experiments or functional assays, offering a systematic characterization of mutations in cancer genes.

### Significant regions provide clinical context for interpreting sparsely annotated mutations

We asked whether significant regions identified by our model offered useful information for interpreting mutations in clinical workflows. In practice, clinical interpretation of somatic mutations in cancer genes relies heavily on expert-curated knowledge bases.^57^ Yet, these resources evolve continuously, differ in scope and evidence criteria, and often disagree on which mutations they annotate as oncogenic. When we compared widely used knowledge bases^48–50,58,59^ (COSMIC, CIViC, ClinVar, OncoKB, and CKB), only 40.4% of mutations were labeled consistently by at least two independent resources (Figures 6G and S26A). This limited concordance persisted when restricting to top-tier annotations (29.6%).

Notably, the fraction of mutations falling within significant regions gradually increased with the number of supporting databases (Figures 6H and S26B), indicating that significant regions tracked with growing confidence in oncogenicity. Furthermore, oncogenic annotations tended to cluster close to one another (Figure S26C), suggesting that significant regions could offer clinically relevant context, especially for mutations with limited or conflicting annotations in existing knowledge bases.

We found several lines of evidence supporting this possibility. First, in mutagenesis data from cell-line screens,^60–62^ singly annotated mutations (i.e., those labeled as oncogenic by one database but none of the others) showed significantly stronger functional effects in significant vs. non-significant regions for *TP53* (*p* = 5.58 × 10^−16^; Mann-Whitney U test), *VHL* (*p* = 1.46 × 10^−5^), and *PTEN* (*p* = 6.45 × 10^−7^) (Figure S26D). Second, ClinVar annotations accumulated disproportionately within significant regions over time, particularly after accounting for their smaller size (Figure S26E), indicating that mutations in significant regions were more likely to become annotated as oncogenic in future releases than those in non-significant regions. Third, significant regions were enriched for oncogenic annotations from ClinVar, CKB, COSMIC, OncoKB, and CIViC (Figure S26F)—even after excluding mutations also annotated by other databases (Figure S26G), showing that significant regions captured oncogenic mutations beyond those that already reached consensus across databases. Fourth, mutations in significant vs. non-significant regions of the same genes were associated with different patient outcomes, informing their clinical interpretation (Figures 6E, 6F, S24, and S25).

Together, significant regions not only recovered well-established oncogenic mutations but may also help prioritize less-well-annotated mutations for further clinical evaluation (Figures 6G, 6H, and S26). By defining the boundaries of significant regions and their structural and functional context, our study distinguished mutations in functionally relevant regions vs. those in less-informative parts of the same genes. Specifically, significant regions tracked with increasing confidence across expert-curated knowledge bases (Figures 6H, S26A, and S26B), and mutations in these regions showed stronger associations with gene expression, transcription factor activity, downstream signaling, clonality, survival, and therapy response (Figures 6A–6F and S21–S25). Particularly in settings where annotations are sparse, conflicting, or still evolving, significant regions may help reviewers prioritize mutations for closer evaluation, support more consistent interpretation across knowledge bases, and identify potentially oncogenic mutations before they reach broad consensus. Combined with expert curation, they may provide meaningful context for interpreting mutations that would otherwise remain uncertain.

## DISCUSSION

Over the past decade, hundreds of genes have been nominated as “significantly mutated” in cancer.^3,4^ Our study adds a functional layer to this increasingly “long tail” of potentially cancer-relevant genes by providing a standardized proteo-genomic atlas across 173 genes in 139,818 patients, defining the boundaries of significantly mutated regions, and relating them to protein domains, three-dimensional structural constraints, mutational mechanisms, multi-omics phenotypes, clinical outcomes, and expert-curated annotations. By integrating proteomic and genomic information, our framework captured a broad spectrum of mutation architectures—ranging from focal hotspots, narrow mutational clusters, and domain-level enrichments to alterations distributed across interaction surfaces, structurally constrained residues, and positional preferences of truncating mutations that spanned large parts of the gene and were shaped by nonsense-mediated decay and post-translational degradation motifs. Our atlas shifts the interpretation of mutations from the gene to the region level, distinguishing functionally informative parts from those in less-informative regions within cancer genes.

While mutations in canonical cancer genes have been characterized in great mechanistic detail,^60^ functional studies typically focus on one or a few genes at a time, using different experimental systems and readouts.^63^ By transforming lists of mutated genes into harmonized maps of significantly mutated regions, our unified approach consolidated these observations, grouped cancer genes with similar spatial architectures, distinguished different modes of gene (in)activation, and enabled principles learned from well-characterized cancer genes to be extended to less-studied genes with similar mutation patterns. Besides established patterns in canonical cancer genes, our framework also supported the interpretation of mutations in less-established genes and domains (e.g., *CDH1*, *PIK3R1*, *POT1*, *PPM1D*, *XPO1*, or a non-canonical region in *KRAS*), tumor-type-specific differences (e.g., those in *CCND1* and *NOTCH1*), and mutations with sparse, conflicting, or evolving annotations in expert-curated knowledge bases. This is particularly relevant as sequencing studies in precision oncology shift from broad discovery cohorts toward increasingly specific clinical settings^64^ (e.g., resistance or exceptional response to a defined therapy), where sample sizes are often limited and the central challenge is not to discover entirely new cancer genes, but to evaluate whether mutations in known cancer genes affect functionally meaningful regions.^65^ Our atlas provides a reference map for interpreting mutations observed in future clinical cohorts based on their location within significant regions and associated functional and structural features.

Two recent developments are instrumental for this study. First, the growing scale of tumor sequencing data provides the necessary resolution for characterizing spatial distributions of mutations in cancer genes.^12,13^ Earlier studies, such as The Cancer Genome Atlas (TCGA), lack the size for these fine-grained analyses^1,2^ and identify mutated cancer genes in aggregate,^3–5^ rather than analyzing spatial distributions of their mutations. Second, deep-learning models provided highly accurate predictions of the protein structures of almost all cancer genes,^14^ allowing their use for systematic genomic analyses through the lens of structural biology. Until recently, structural analyses required resource-intensive experiments, which are available only for well-studied cancer genes and often limited to subdomains. Building on these advances, we integrated proteomics^14,15^ with population-scale cancer genomics data^12,13^ to detect subtle—yet biologically meaningful—mutation patterns in cancer genes and link them to structural and functional constraints shaping their effects in tumor development.

More broadly, our study shows the potential of proteo-genomic strategies in precision medicine. Biologically, they can resolve mutation patterns that appear diffuse in linear genomic sequences but converge in three-dimensional protein structures, where they can be linked to structural elements, interaction surfaces, ligand-binding sites, or regulatory mechanisms. In tumor suppressors, for example, our framework distinguished multiple modes of inactivation: missense mutations in α-helices might perturb protein-protein interactions, particularly when they affected residues on exposed helical faces,^66^ whereas mutations accumulating in β-sheets were more consistent with compromised stability, especially when mutations disrupted structural scaffolds for strand pairing or packing.^67^ Even truncating mutations, often assumed to spread uniformly within cancer genes,^17^ exhibited clear regional preferences related to nonsense-mediated decay.^18^ That way, proteomics-guided dissection of mutation patterns could help move beyond labels of oncogenes vs. tumor suppressors toward a more nuanced assessment of mutations and their multifaceted roles in tumorigenesis.^19^

Clinically, proteo-genomics strategies could further complement curated expert knowledge, particularly for mutations with sparse or conflicting annotations, and help harmonize interpretations in clinical workflows. This is particularly relevant as several pathogenicity scores were not primarily designed to detect tumor-specific signals and yet are often utilized to prioritize somatic mutations in cancer genes. While our atlas is not intended to assign definitive functional or clinical significance, it provides standardized context for prioritizing mutations and integrating them with existing biological and clinical knowledge. Ultimately, as genome sequencing data continue to grow in many areas of precision medicine, we hope that proteo-genomic strategies will support many genome-driven tasks, including the stratification of patients into clinically informative subgroups.

To support these initiatives across the broader research community, we provide our proteo-genomic framework and its functional maps as an interactive resource online (oncofold.org), with an interactive view of significant regions within three-dimensional protein structures, diverse tracks for contextualizing mutation patterns, and options for customized search and downloads of annotations from our framework.

## RESOURCE AVAILABILITY

### Lead contact

Requests for further information and resources should be directed to the lead contact, Felix Dietlein (felix.dietlein@childrens.harvard.edu).

### Materials availability

This study did not generate new reagents.

### Data and code availability

- An interactive resource to visualize significant regions in cancer genes and map them on their protein structures is available online (oncofold.org).
- Coordinates of significant regions, the source code of our proteo-genomics model, a user manual, and a test dataset are available on Zenodo (https://zenodo.org/records/21430016).
- Clinical annotations and somatic whole-genome sequencing data from the CPCT and the HMF were obtained through request DR-391. Requests for these data can be submitted at https://www.hartwigmedicalfoundation.nl/en/data/data-access-request/. AACR GENIE data were downloaded through Synapse (https://synapse.org/genie) and cBioPortal (https://genie.cbioportal.org/). Treatment data for patients in AACR GENIE (Biopharma Collaborative) cohorts were obtained through request ACT-3038. All other data are publicly available and links are provided in the STAR Methods.
- Any additional information required to reanalyze the data reported in this paper is available from the lead contact upon request.

## STAR★METHODS

### METHOD DETAILS

#### Somatic mutation dataset and filtering criteria

We downloaded the AACR Project GENIE dataset^12,13^ (version 15.0; December 27, 2023) from synapse.org (https://doi.org/10.7303/syn53210170). We analyzed all protein-coding SNVs from this dataset, and we excluded synonymous mutations, due to inconsistencies in their reporting across GENIE genomic profiling centers (most sequencing sites did not report them at all).

To identify and exclude potential germline variants, we cross-referenced mutations with gnomAD^68,69^ (v4; 730,947 normal whole exomes and 76,215 normal whole genomes) and ExAC^70^ (final release; 60,706 normal whole exomes), which we downloaded from https://gnomad.broadinstitute.org/downloads. GnomAD variants were lifted over from Hg38 to Hg19 (chain files downloaded from the UCSC Genome Browser^71^; https://hgdownload.soe.ucsc.edu/goldenPath/hg38/liftOver/). Mutations in GENIE with allelic counts ≥10 in gnomAD and ExAC datasets were excluded from our analysis as putative germline variants.

To ensure uniform coverage, we further determined the fraction of protein-coding positions covered by each assay in GENIE^12,13^ (genomic_information.txt file in AACR GENIE). Assays that cover only hotspot positions or subregions of a gene were excluded. Only mutations from assays with ≥98% gene coverage were included to avoid confounding effects due to partial gene coverage.

We performed all subsequent analyses separately for either missense mutations, nonsense mutations or both types of mutations in combination, as they revealed distinct insights into the biology of cancer genes.

#### Identification of significantly mutated regions in cancer genes

Let ℓ denote the total number of substitution positions for a given gene. For simplicity of the notation, we annotated each possible nucleotide substitution as a distinct index (e.g., C>A and C>T substitutions of the same base pair were indexed separately). Positions excluded as germline variants, low coverage sites, or synonymous mutations in the previous step were not included in the index in our notation. Furthermore, as we performed this analysis either for missense mutations, nonsense mutations, or both mutation types, the index iterates only over those mutation type(s) included in the analysis.

For each position i∈{1,…,ℓ}, we defined $n_{i}\in N_{\geq 0}$ as the observed number of somatic mutations at position i. We further determined $m_{i}\in R_{\geq 0}$, the background mutability of position i, based on its surrounding genomic sequence context. For this, we distinguished 192 sequence contexts^90^ (5′ and 3′ flanking bases and 12 possible substitution types) and computed the ratio of the total number of mutations to the total number of covered positions within each context. Lastly, we defined $e_{i}$ as the functional effect of the mutation at position i, with $e_{i}=0$ for synonymous mutations, $e_{i}=1$ for nonsense mutations, and $e_{i}\in [0,1]$ for missense mutations as predicted by AlphaMissense^15^ (a tool that compares original vs. mutated amino acids based on their relevance in AlphaFold2 structures; AlphaMissense_hg19.tsv.gz on https://console.cloud.google.com/storage/browser/dm_alphamissense; “am_pathogenicity” column, see method details in the AlphaMissense publication).^15^

##### Overview of the Baum-Welch algorithm

To identify significantly mutated regions in cancer genes, we trained a Hidden Markov model for distinguishing non-significant regions (baseline state) vs. significant regions (other states in the model). For a Hidden Markov model with s hidden states (s is provided as a training parameter, see below), we defined $\omega_{i,j}\in [0,1]$ for each state j∈{1,…,s} as the probability that position i is assigned to state j, with $\sum_{j=1}^{s}\omega_{i,j}=1\forall i\in \{1,\ldots ,\ell \}$. We further defined $\theta_{i,j}\in R_{\geq 0}$ as the expected number of mutations at position i in state j, which we factorized as $\theta_{i,j}=m_{i}\cdot f_{j}\left(e_{i}\right)$, with $f_{j}:[0,1]↤R_{\geq 0}$ being the state-specific function that maps effect scores to relative mutation rates. We then computed the probability $p_{i,j}\in [0,1]$ of observing $n_{i}$ mutations at position i in state j, via a Poisson-lognormal distribution as $p_{i,j}=P\left(Y_{\text{PLN}}=n_{i}|\theta_{i,j},\nu \right)$, denoting the point probability of a random variable $Y_{\text{PLN}}$ distributed by a Poisson-lognormal distribution with distribution mean $\theta_{i,j}$ and variance ν of the normal component.

$$P\left(Y_{PLN}=n_{i}|\theta_{i,j},\nu \right)=\int_{0}^{\infty }P\left(Y_{\text{Poisson}}=n|\lambda \right)\cdot f_{\text{log-norm}}\left(\lambda |log\;\theta -0.5\cdot \nu^{2},\nu \right)\cdot d\lambda$$

We fixed variance $\nu \in R_{\geq 0}$ across all states to prevent overfitting into discrete, low-variance states.

Our goal was to estimate weights $\omega_{i,j}$ that best explain the observed mutation counts $n_{i}$ while avoiding overfitting. For this, we employed a penalized version of the Baum-Welch algorithm to train and optimize a Hidden Markov Model. $\omega_{i,j}$ and $f_{j}$ are learned by iterative training of the Hidden Markov model via the Baum-Welch algorithm and $\theta_{i,j},p_{i,j}$, and ν are iteratively updated based on these parameters. By default, we set j=1 as the baseline state and defined $f_{1}\left(e_{i}\right)=c$ as a constant function, indicating that its mutation rates depend solely on their background mutability but not its effect $e_{i}$. We further included a small probability offset γ≥1 to state j=1 (i.e., $p_{i,1}=\left.\gamma \cdot P\left(Y_{PLN}=n_{i}|\theta_{i,j},\nu \right)\right)$ to ensure the model remained in the baseline state, unless sufficient evidence supported a different state.

###### 1. Parameters of the algorithm:

Our implementation requires specifying four training parameters.

s the number of states in the Hidden Markov Model (see above),

τ the index at which counts $n_{i}$ are capped (see below),

γ the probability offset of the baseline state (see below), initialization strategy: uniform or random strategy to initialize weights (see below).

The Baum-Welch algorithm further uses the following internal variables, which it initializes and iteratively updates throughout the course of model training.

$\pi_{j}$ the prior probability of state j∈{1,…,s},

$\alpha_{i,j}$ the probability of state j at position i, considering the data in positions 1,…,i-1

(forward variable)

$\beta_{i,j}$ the probability of state j at position i, considering the data in positions i+1,…,ℓ

(backward variable)

In addition, we define a fixed s×s matrix $A\in R_{\geq 0}^{s\times s}$ of transition probabilities from state j to $j^{\prime }$ as

$$A_{j,j^{\prime }}=\left\{\begin{array}{cc} 1-\ell^{-1} & \;\text{for}\;j=j^{\prime }\\ \ell^{-1}\cdot (s-1{)}^{-1} & \;\text{for}\;j\neq j^{\prime } \end{array}\right.$$

This matrix penalizes unnecessary state changes, ensuring that the model remains in the same state, unless sufficient evidence in the data justifies a transition. To avoid hotspots dominating the solution, we capped counts $n_{i}$ to the (ℓ-τ) th largest count in each gene during the training of the model (no cap if τ=0), where τ is specified as a parameter for the algorithm (see above).

###### 2. Initialization phase:

We initialized weights $\omega_{i,j},i\in \{1,\ldots ,\ell \}$ and j∈{1,…,s}, either by

random initialization: $\omega_{i,j}\sim Uniform(0;1)$ or

uniform initialization: $\omega_{i,j}=(0.95+0.1\cdot x)/s$, where x~Uniform(0;1)

followed by normalizing $\sum_{j=1}^{s}\omega_{i,j}$ to 1.0 for each position i∈{1,…,ℓ}.

We used both strategies, as we found that the convergence behavior of the algorithm sometimes varies depending on the initialization strategy, if there were multiple local optima.

Based on the initialized weights $\omega_{i,j}$, we computed $\theta_{i,j},p_{i,j}$, and ν for each position i∈{1,…,ℓ} and state j∈{1,…,s} (see method details below). We further initialized the prior state probabilities uniformly as $\pi_{j}=s^{-1}$.

###### 3. Iterative training phase:

Each iteration of the Baum-Welch algorithm consists of a forward and backward pass, followed by weight updates and parameter recalculations.

For the forward pass, we initialized $\alpha_{1,j}=\pi_{j}\cdot p_{1,j}$ and recursively set $\alpha_{i,j}=\sum_{k=1}^{s}\alpha_{i-1,k}\cdot A_{k,j}\cdot p_{i,j}$ for ascending i=2,…,ℓ and j∈{1,…,s}. For the backward pass, we set $\beta_{\ell ,j}=1$ and recursively computed $\beta_{i,j}=\sum_{k=1}^{s}\beta_{i+1,k}\cdot A_{j,k}\cdot p_{i+1,k}$ for descending i=ℓ-1,…,1 and j∈{1,…,s}.

Based on $\alpha_{i,j}$ and $\beta_{i,j}$, we then updated the weights of each position i∈{1,…,ℓ} and state j∈{1,…,s}

$$\omega_{i,j}=\frac{\alpha_{i,j}\cdot \beta_{i,j}}{\sum_{k=1}^{s}\;\alpha_{i,k}\cdot \beta_{i,k}}$$

Based on these updated weights $\omega_{i,j}$, we recomputed $\theta_{i,j},\nu$, and $p_{i,j}$ (see below). We also updated the prior probabilities $\pi_{j}=\omega_{0,j},\;j\in \{1,\ldots ,s\}$, for the next iteration.

We repeated this procedure for a total of 50 iterations. Empirically, we found that it required several iterations for $\omega_{i,j}$ to stabilize after updating $\theta_{i,j},\nu$, and $p_{i,j}$. Since $\theta_{i,j},\nu$, and $p_{i,j}$ are resource-intensive to compute, we updated these variables only every 5 iterations. We capped $\alpha_{i,j},\beta_{i,j},p_{i,j},\omega_{i,j}$, and $\pi_{j}$ at a minimum of $10^{-5}$ to avoid overfitting to outliers and stored them in their logarithmic form to maintain numerical precision across iterations.

####### 4. Evaluation phase:

In the final evaluation phase, we updated $\theta_{i,j},\nu$, and $p_{i,j}$ based on the final weights $\omega_{i,j}$ one final time. We then determined the most likely state for each position i∈{1,…,ℓ}

$${\hat{\omega }}_{i}=\underset{j\in \{1,\ldots ,s\}}{arg\;max}\omega_{i,j}$$

and computed the total log likelihood of the solution with these final parameter estimates as

$$L=\sum_{i=1}^{\ell }logP\left(Y_{PLN}=n_{i}|\theta_{i,{\overset{ˆ}{\omega }}_{i}},\nu \right)$$

with $Y_{\text{PLN}}$ distributed according to a Poisson-lognormal distribution with distribution mean $\theta_{i,{\overset{ˆ}{\omega }}_{i}}$ and variance ν of the normal component. Unlike in the initialization and training phases, counts $n_{i}$ were not capped in the evaluation phase to ensure that the final log likelihood reflects how well the model explains the original, unmodified count data.

###### Computation of $\theta_{i,j},\nu$, and $p_{i,j}$

We updated $\theta_{i,j}$, after new weights $\omega_{i,j}$ were set for each state and position by calculating the local average of counts in positions in the same state with similar effects $e_{i}$. We then determined variance ν via maximum-likelihood estimation and probabilities $p_{i,j}$ as a Poisson-lognormal distribution with these updated parameters.

####### 1. Computation of predicted mutation counts:

To compute the predicted mutation count $\theta_{i,j}$ at position i in state j, we distinguished between positions related to missense and nonsense mutations, which we denoted by ℳ and 𝒩, respectively.

For nonsense mutations i∈𝒩, we computed $\theta_{i,j}$ as a weighted average of observed and background mutation counts from all other nonsense positions in the same state j∈{1,….s} as

$$\theta_{i,j}=\left(\frac{\sum_{k\in 𝒩,k\neq i}\;\omega_{k,j}\cdot n_{k}}{\sum_{k\in 𝒩,k\neq i}\;\omega_{k,j}\cdot m_{k}}\right)\cdot m_{i}$$

To compute $\theta_{i,j}$ for missense mutations i∈ℳ, we accounted for the predicted effect $e_{i}$ of each mutation. We sorted positions based on their effects $e_{i}$ and reindexed their associated variables accordingly as ${\tilde{e}}_{i},{\tilde{m}}_{i},{\tilde{n}}_{i}$, and ${\tilde{\omega }}_{i,j}$ (i.e., index i refers to the position with the i th largest effect ${\tilde{e}}_{i}$). We then defined the cumulative weight percentile for each i∈ℳ as $\Omega_{i,j}=\sum_{k\in ℳ,k\leq i}{\tilde{\omega }}_{k,j}/\sum_{k\in ℳ}{\tilde{\omega }}_{k,j}$. Predictions ${\tilde{\theta }}_{i,j}$ were then computed as a weighted average of positions with similar effects ${\tilde{e}}_{i}$, using a ±5% window around $\Omega_{i,j}$

$${\tilde{\theta }}_{i,j}=\left(\frac{\sum_{k\in ℳ,k\neq i,\left|\Omega_{k,j}-\Omega_{i,j}\right|\leq 0.05}{\tilde{\omega }}_{k,j}\cdot {\tilde{n}}_{k}}{\sum_{k\in ℳ,k\neq i,\left|\Omega_{k,j}-\Omega_{i,j}\right|\leq 0.05}{\tilde{\omega }}_{k,j}\cdot {\tilde{m}}_{k}}\right)\cdot {\tilde{m}}_{i}$$

We then mapped ${\tilde{\theta }}_{i,j}$ back to their original genomic indices, thereby obtaining $\theta_{i,j}$ for each i∈ℳ and state j∈{1,…s}. The first factor of this equation reflects the mapping function $f_{j}\left(e_{i}\right)$ of state j∈{1,…s}.

####### 2. Maximum-likelihood estimation of the variance:

The variance parameter ν governs the dispersion of the log-normal component in the Poisson-lognormal distribution. Given counts $n_{i}$, predictions $\theta_{i,j}$, and weights $\omega_{i,j}$, we estimated ν by maximizing the overall weighted log likelihood

$$\nu =\underset{\nu \in (0,10)}{argmax}\sum_{i=1}^{\ell }\sum_{j=1}^{s}\omega_{i,j}\cdot logP\left(Y_{PLN}=n_{i}|\theta_{i,j},\nu \right)$$

with $Y_{\text{PLN}}$ randomly distributed according to a Poisson-lognormal distribution with distribution mean $\theta_{i,j}$ and variance ν of the normal component. Because this distribution does not have a closed form (see method details below), we identified the maximum likelihood by an exhaustive grid search of ν between 0.0 and 10.0, in 0.01 increments. Given the scale of this computation (ℓ⋅s is typically large; likelihoods have to be computed over ℓ⋅s summands for 1000 candidate ν), we used a GPU to compute the 1000 likelihood sums in parallel (see method details below).

####### 3. Computation of point probabilities:

We computed point probabilities $p_{i,j}$ of observing $n_{i}$ mutations at position i∈{1,…,ℓ}, given prediction $\theta_{i,j}$ and variance ν. The point probability of a Poisson-lognormal distribution with distribution mean θ and variance ν of the normal component is defined as

$$P\left(Y_{PLN}=n|\theta ,\nu \right)\;=\int_{0}^{\infty }\;P\left(Y_{\text{Poisson}}=n|\lambda \right)\cdot f_{log\text{-norm}}\left(\lambda |log\;\theta -0.5\cdot \nu^{2},\nu \right)\cdot d\lambda \;\;=\int_{0}^{\infty }\;P\left(Y_{\text{Poisson}}=n|\lambda \right)\cdot \lambda^{-1}\cdot f_{\text{norm}}\left(log\;\lambda |log\;\theta -0.5\cdot \nu^{2},\nu \right)\cdot d\lambda$$

with the point probability of a Poisson distribution and probability density of a normal distribution

$$P\left(Y_{\text{Poisson}}=n|\lambda \right)=\frac{\lambda^{n}e^{-\lambda }}{n!}\;f_{\text{norm}}(\lambda |\theta ,\nu )=\frac{1}{\sqrt{2\pi \nu }}\cdot exp\left(\frac{(\lambda \;-\;\theta {)}^{2}}{-2\nu }\right)$$

Since the Poisson-lognormal distribution has no closed-form solution, we approximated it by summing over a grid of discrete λ values (grid range: $\pm 5\cdot max(1,\sqrt{\nu })$ around the log mean; step size: 0.1). Since these computations are often repeated with similar parameters (e.g., for maximum likelihood estimation of the variance), we precomputed a lookup table of Poisson-lognormal probabilities for a wide range of n,θ, and ν parameters, and we used a GPU to parallelize the computation of the lookup table. That way, Poisson-lognormal probabilities were readily available in all subsequent computation steps (see method details on the approximation and GPU-based parallelization below).

Using this lookup table, we computed $p_{i,j}=P\left(Y_{PLN}=n_{i}|\theta_{i,j},\nu \right)$ for positions i∈{1,…,ℓ} and states j≥2. For baseline state j=1, we used a small probability offset γ≥1 to define the probability as $p_{i,j}=\gamma \cdot P\left(Y_{PLN}=n_{i}|\theta_{i,j},\nu \right)$, ensuring the model remains in the baseline state unless sufficient evidence in the data justifies another state. The probability offset γ is chosen as a training parameter of the algorithm.

###### Selection of the optimized Hidden Markov model

We trained Hidden Markov models with different numbers of states s∈{1,…,5}, probability offsets γ∈{1.0,1.25,1.5,2.0,5.0}, cutoffs τ∈{0,2,5,10,25}, and uniform vs. random initialization strategies. For each combination of these parameters, we trained 10 independent models, since random initializations can sometimes converge to different local optima. That way, we obtained for each combination s,γ,τ a set of likelihoods $L_{s,\gamma ,\tau }=\left\{L_{s,\gamma ,\tau ,a,b}|a\in \{1,\ldots ,10\},b\in \{\right.rand,\;unif\}$, with $L_{s,\gamma ,\tau ,a,b}$ denoting the likelihood of the model trained with parameters s,γ,τ in the ath iteration and initialization strategy b.

Our goal was to find parameters s,γ,τ that best explained the observed data, while avoiding unnecessarily complex solutions. To compare the likelihoods L and $L^{\prime }$ from different parameter settings, we defined their normalized distance as

$$dist\left(L,L^{\prime }\right)=\frac{avg(L)\;-\;avg\left(L^{\prime }\right)}{max\left(stdev(L),stdev\left(L^{\prime }\right),stdev\left(L,L^{\prime }\right)\right)}$$

with the pooled standard deviation

$$stdev\left(L,L^{\prime }\right)=\sqrt{\frac{stdev(L{)}^{2}\cdot (|L|\;-\;1)\;+\;stdev{\left(L^{\prime }\right)}^{2}\cdot \left(\left|L^{\prime }\right|\;-\;1\right)}{|L|\;+\;\left|L^{\prime }\right|\;-\;2}}$$

This metric quantifies by how many standard deviations model L (with potentially higher complexity) exceeds the likelihood of a model $L^{\prime }$. Since complex solutions typically explain observed data better, our requirement was dist>5 (i.e., an improvement of 5 standard deviations) to pick a more “complex” over a “simple” solution, commonly referred to as the 5-sigma rule. This rule is commonly used to pick a model that explains the data reasonably well, while avoiding overfitting or unnecessarily complex solutions.

####### 1. Partitioning of solutions:

We noticed that models with the same parameter settings sometimes yielded solutions that significantly differed in their likelihood. Thus, we partitioned each $L_{s,\gamma ,\tau }$ into 2 or 3 components $L_{s,\gamma ,\tau }^{\left(1\right)},L_{s,\gamma ,\tau }^{\left(2\right)},L_{s,\gamma ,\tau }^{\left(3\right)}$ using a Gaussian mixture model (via sklearn.mixture.GaussianMixture in Python) and we evaluated whether these components differed significantly in their likelihood based on the 5-sigma rule. If partitions differed by more than 5 standard deviations, i.e., $\underset{i\neq j}{max}dist\;(L_{s,\gamma ,\tau }^{\left(i\right)},L_{s,\gamma ,\tau }^{\left(j\right)},)>5$, we selected the component with ${\tilde{L}}_{s,\gamma ,\tau }=arg\;\underset{i}{max}\;\left\{avg\left(L_{s,\gamma ,\tau }^{\left(i\right)}\right)||L_{s,\gamma ,\tau }^{\left(i\right)}|\geq 3\right\}$. Otherwise, we retained all solutions and set ${\tilde{L}}_{s,\gamma ,\tau }=L_{s,\gamma ,\tau }$.

####### 2. Optimizing training parameters:

We further used the 5-sigma rule to iteratively pick the best solution, while avoiding unnecessary complexity. For this, we iterated from low-to high-complexity solutions and evaluated whether they significantly improved the likelihood. We initialized $L_{\text{opt}}={\tilde{L}}_{1,1,0,0}$ (trivial solution with only one state) and iterated through the parameters in the following order:

for s in 1, ...,5:

for τ in 25, 10, 5, 2, 0:

for γ in 1.0, 1.25, 1.5, 2.5, 5.0:

if $dist\left({\tilde{L}}_{s,\tau ,\gamma },L_{\text{opt}}\right)>5$:

then update $L_{\text{opt}}={\tilde{L}}_{s,\tau ,\gamma }$

After these iterations, we arrived at an optimized solution $L_{\text{opt}}$. We referred to $L_{\text{opt}}^{\left(1\right)}$ as the most recent selection, $L_{\text{opt}}^{\left(2\right)}$ as the second most recent, etc.

###### Summarizing weights across solutions

After identifying the optimal parameter selection, our next goal was to summarize solutions in $L_{\text{opt}}$ into a robust set of weights. A key reason we could not simply average weights across solutions was that states are not necessarily discovered in the same order (e.g., the 2nd state of solution 1 might correspond to the 3rd state in solution 2) and states did not always align across solutions (e.g., several local optima).

For $K=\left|L_{\text{opt}}\right|$ solutions to summarize, we denoted by $\omega_{i,j}^{\left(k\right)}$ the weights in solution k∈{1,…,K}, each with $s^{\left(k\right)}$ states (we did not require the number of states to be the same in each solution), positions i∈{1,…,ℓ}, and states $j\in \left\{1,\ldots ,s^{\left(k\right)}\right\}$. We pursued two orthogonal strategies to summarize solutions.

####### 1. Summary of similar states:

Our first strategy was to identify states that showed similar spatial patterns along the gene and we gradually summarized these states from different solutions. We defined the distance between two states – state $j_{1}$ from solution $k_{1}$ and state $j_{2}$ from solution $k_{2}$ – as

$$\begin{array}{c} dist\left(\left(k_{1},j_{1}\right);\left(k_{2},j_{2}\right)\right)=\sqrt{\sum_{i=1}^{e}\;{\left(\omega_{i,j_{1}}^{\left(k_{1}\right)}-\omega_{i,j_{2}}^{\left(k_{2}\right)}\right)}^{2}}\\ \;\text{for}\;1\leq k_{1},k_{2}\leq K,1\leq j_{1}\leq s^{\left(k_{1}\right)},\;\text{and}\;1\leq j_{1}\leq s^{\left(k_{1}\right)}. \end{array}$$

Based on this distance metric, we performed hierarchical clustering on $\left\{(k,j)|1\leq k\leq K,1\leq j\leq s^{\left(k\right)}\right\}$ with minimum linkage. Importantly, we only merged clusters $C_{1},C_{2}$ with $k_{1}\neq k_{2}\forall \left(k_{1},j_{1}\right)\in C_{1},\left(k_{2},j_{2}\right)\in C_{2}$ (i.e., states from the same solution are not merged). We terminated if there were no more clusters to summarize based on this rule. That way, we arrived at clusters $C^{\left(1\right)},\ldots ,C^{\left(n\right)}$ and we summarized their weights, for i∈{1,…,ℓ} and n∈{1,…,N}, as

$$\vartheta_{i,n}={avg}_{(k,j)\in C^{\left(n\right)}}\omega_{i,j}^{\left(k\right)}$$

Lastly, we removed “unutilized” states $n^{\prime }$ with $\vartheta_{i,n^{\prime }}<\underset{1\leq n\leq N}{max}\;\vartheta_{i,n}\forall i\in \{1,\ldots ,\ell \}$. We added weights $\vartheta_{i,n^{\prime }}$ from discarded states $n^{\prime }$ to the most similar state m

$$m=\underset{1\leq m\leq N}{arg\;min}\sqrt{\sum_{i=1}^{e}\;{\left(\vartheta_{i,n^{\prime }}-\vartheta_{i,m}\right)}^{2}}$$

That way, we arrived at a final set of N summarized states and weights $\vartheta_{i,n}$.

######## 2. Summary of similar positions:

As an orthogonal strategy, we identified positions that were assigned to similar states across solutions. Each summarized state then represented a subset of positions that were assigned to the same states across solutions. This strategy had the advantage that it did not require selecting “similar” states but summarized states in a fully agnostic manner.

For positions $i,i^{\prime }\in \{1,\ldots ,\ell \}$, we defined the following distance metric

$$dist\left(i,i^{\prime }\right)=\sqrt{\sum_{k=1}^{K}\;\sum_{j=1}^{s^{\left(k\right)}}\;{\left(\omega_{i,j}^{\left(k\right)}-\omega_{i^{\prime },j}^{\left(k\right)}\right)}^{2}}$$

reflecting whether i and $i^{\prime }$ were assigned to similar states across different solutions.

For clusters C⊆{1,…,ℓ}, we defined their centroids as ${\overset{‾}{\omega }}_{C,j}^{\left(k\right)}={avg}_{i\in C}\omega_{i,j}^{\left(k\right)}$ for k∈{1,…,K} and $j\in \left\{1,\ldots ,s^{\left(k\right)}\right\}$ and their distance metric as

$$dist\left(C,C^{\prime }\right)=\sqrt{\sum_{k=1}^{K}\;\sum_{j=1}^{s^{\left(k\right)}}\;{\left({\overset{‾}{\omega }}_{C,j}^{\left(k\right)}-{\overset{‾}{\omega }}_{C^{\prime }j}^{\left(k\right)}\right)}^{2}}$$

Using these distance metrics, we used hierarchical clustering to identify groups of positions that were assigned to similar states. We terminated once we reached N clusters (N chosen from strategy 1). To reduce the computational cost (the number of pairwise comparisons $\ell^{2}\gg 0$ is large), we only merged adjacent positions and clusters for the initial ℓ-100 clustering rounds, and we considered all pairs in the final 100 iterations. This made little difference as the number of state transitions is much smaller than 100, and positions in the same state are part of the same cluster by the definition of the metric. That way, we arrived at N clusters $C^{\left(1\right)}\dot{\cup }\ldots \dot{\cup }C^{\left(N\right)}=\{1,\ldots ,\ell \}$, each summarizing positions that were assigned to similar states across the K solutions.

To assign weights $\phi_{i,n}$ based on this partition, we performed a soft cluster assignment, measuring the distance of each position i∈{1,…,ℓ} to each cluster centroid ${\overset{‾}{\omega }}_{C^{\left(n\right)},j}^{\left(k\right)}$, with n∈{1,…,N}. We defined

$$\phi_{i,n}=\frac{f_{exp}(dist(i,C^{\left(n\right)})|\lambda )}{\sum_{n^{\prime }=1}^{N}f_{exp}(dist(i,C^{\left(n^{\prime }\right)})|\lambda )}$$

where $f_{\text{exp}}(d|\lambda )=\lambda \cdot e^{-\lambda \cdot d}$ is the probability density function of the exponential distribution.

We optimized its decay parameter λ to best explain the observed distribution

$$\lambda =arg\;\underset{\lambda }{max}\;\sum_{i=1}^{e}\sum_{n=1}^{N}log\;f_{exp}(dist(i,C^{\left(n\right)})|\lambda )$$

######## 3. Harmonizing both strategies:

To harmonize weights $\vartheta_{i,n}$ and $\phi_{i,n}$ from both strategies, we reordered the states of $\phi_{i,n}$ to best match $\vartheta_{i,n}$ by minimizing $dist\left(n,n^{\prime }\right)=\sqrt{\sum_{i=1}^{\ell }\;{\left(\vartheta_{i,n}-\phi_{i,n^{\prime }}\right)}^{2}}$. That way, our final weights captured both state- and position-level similarity across solutions in $L_{\text{opt}}$.

####### Implementation of the Hidden Markov model on a GPU

Since the Poisson-lognormal distribution does not have a closed form, we approximated it using a fine summation grid. Furthermore, given counts $n_{i}$ and predictions $\theta_{i}$, we optimized the variance as a maximum likelihood estimation via an exhaustive grid search. Both tasks required substantial compute time (see above) but could be efficiently parallelized using CUDA kernels on NVIDIA GPUs.

We developed our code on a workstation with an AMD Ryzen Threadripper PRO 3995WX (64 cores), 256 GB memory, 3x NVIDIA GeForce RTX3090, and 1x NVIDIA RTX A5000 (Windows OS). To run our code on a larger number of genes, we launched multiple, parallel instances in the Lambda Cloud (https://cloud.lambda.ai/), each with 30 CPU cores, 200 GB memory, and 1x NVIDIA A10 Tensor Core GPU or 1x NVIDIA A100 Tensor Core GPU (gpu_1x_a100_smx4 or gpu_1x_a10) (Ubuntu OS). A run of our code across all genes required ~20 h on 8 instances with an A100 or 19 instances with an A10.

To configure GPUs for the use of CUDA kernels, we downloaded NVIDIA drivers (https://www.nvidia.com/Download/index.aspx) as well as the CUDA developer toolkit (https://developer.nvidia.com/cuda-downloads), including the NVIDIA CUDA compiler nvcc (version 12.5.40). We verified the correct setup of drivers and toolkit using the following commands both on Windows and Ubuntu OS.

nvcc –version

nvidia-smi

######## 1. CUDA kernel source code:

GPU commands of the CUDA kernel were written in C++ (cu file; see https://docs.nvidia.com/cuda/cuda-compiler-driver-nvcc/for details). CUDA functions that were accessed by the host were labeled as *global* and functions that were executed only internally by the GPU as *device*. We compiled the cu file into a ptx file using the CUDA compiler.

nvcc -ptx <path to cu file> -o <path to ptx file>

When moving the cu code to a different instance (e.g., for cloud computing), we recompiled the cu code to optimize it for the local GPU architecture.

We further used JCuda (http://www.jcuda.org/downloads/downloads.html; version 10.1.0) in Java to allocate GPU memory, copy variables from the host memory to the GPU, launch the CUDA kernel, execute CUDA functions, synchronize them, and copy their results back from the GPU to the host memory.

######## 2. CUDA kernel configuration:

To parallelize *K* tasks, we launched a CUDA kernel with ⌈*K*/256⌉ blocks and 256 threads per block (dimensions: x = 256, y = 1, z = 1). For example, for 1000 parallel tasks, we launched a CUDA kernel with 5 blocks, each with 256 threads. Within each thread, we computed its task index *k* as

k=blockIdx.x*blockDim.x+threadIdx.x

with blockDim.x = 256. Within each task, we first checked whether its task index was within bounds *k*<*K* (a few threads in the last block were outside this range and were terminated after this step).

Within task k, the index k was then used to determine which input parameters to read from the input arrays as well as to compute other parameters for the task (see method details below). We further allocated GPU memory to an output array o of length K. After completing its computation, task k wrote its result to position k in the output array o. After all threads finalized their computations, we copied this array back from GPU to the host memory of the computer.

######## 3. Precomputing of the Poisson-lognormal distribution:

To compute a lookup table of the Poisson-lognormal distribution, we varied the variance parameter ν from 0 to 10 in 0.01 increments (1000 values), counts n from 0 to 250 (250 values), and distribution average θ from $1\times 10^{-9}$ to $1.7\times 10^{9}$ in 1.025-fold increments (1700 values). For each combination of these values, we created a separate task to compute the point probability of the Poisson-lognormal distribution, resulting in a total of $K=4.25\times 10^{8}$ tasks (each for a different combination of n,θ,ν).

We prepared input arrays n,θ,ν of length K and copied the values of these input arrays from the host memory of the computer into the memory of the GPU. We further allocated GPU memory to an output array o of length K for storing the output of these K tasks.

Task k then read its input parameters $n_{k},\theta_{k},\nu_{k}$ from position k in these arrays and approximated the integral of the Poisson-lognormal distribution as

$$o_{k}=\frac{\sum_{\lambda \in \Lambda_{k}}P\left(Y_{\text{Poisson}}=n_{k}|\lambda \right)\cdot f_{log-\text{norm}}\left(\lambda |log\;\theta_{k}-0.5\cdot \nu_{k}^{2},\nu_{k}\right)}{\sum_{\lambda \in \Lambda_{k}}f_{log-\text{norm}}\left(\lambda |log\;\theta_{k}-0.5\cdot \nu_{k}^{2},\nu_{k}\right)}$$

with the probability density function of the log-normal distribution

$$f_{log\text{-norm}}(\lambda |log\;\theta ,\nu )=\lambda^{-1}\cdot f_{\text{norm}}(log\;\lambda |log\;\theta ,\nu )$$

and summation grid

$$\Lambda_{k}=\left\{log\;\lambda =log\;\theta_{k}-0.5\cdot \nu_{k}^{2}\pm 0.1\cdot j\left|j\in Z,\left|log\;\lambda -\left(log\;\theta_{k}-0.5\cdot \nu_{k}^{2}\right)\right|\leq 5\cdot max(1,\sqrt{\nu })\right\}\right.$$

After completing its computation, task k wrote this result to position k in the output array o. We then copied the output array o back to the host memory of the computer. In addition, we kept a copy of the array o in the internal memory of the GPU for subsequent tasks on the GPU that required the Poisson-lognormal distribution.

In subsequent computation steps, this table allowed us to instantaneously look up the point probability of the Poisson-lognormal distribution within this grid. In very rare cases, when probabilities were needed for parameters outside the range of the grid (i.e., $n_{k}>250,\;\theta_{k}>1.7\times 10^{9}$, or $\nu_{k}>10$), we computed these point probabilities on the fly. Even though the precompute task consumed a considerable time and memory (typically ~10–15 min on an RTX3090), this strategy outperformed, in our tests, computing Poisson-lognormal probabilities on the fly, since the precomputing task could be parallelized on GPUs highly efficiently and computing Poisson-lognormal probabilities often became the computational bottleneck of subsequent tasks, especially when training a larger number of models in parallel.

######## 4. Maximum-likelihood estimation of the variance:

Given counts $n_{i}$, weights $\omega_{i}$, and predictions $\theta_{i}$, we optimized the variance ν through an exhaustive grid search of ν between 0 and 10 (0.01 increments; 1000 candidates). This required computing a sum $\sum_{i}\omega_{i}\cdot logP\left(Y_{\text{PLN}}=n_{i}|\theta_{i},\nu \right)$ for each ν over a large number of ℓ⋅s≫0 summands, with ℓ the number of positions and s the number of states in the model.

We parallelized the computation of these sums (1000 parallel threads, each for a different ν). For this, we copied arrays n,θ,ω of length I=ℓ⋅s from the host memory to the GPU, executed K=1000 parallel threads with ν=k/100 in thread k, wrote its result to $o_{k}$ in an output array o of length 1000, copied o from the GPU back to the host memory, and selected ν with the maximum likelihood.

In practice, we had a large number of CPU cores (~30–64) optimizing Hidden Markov models and variance parameters in parallel, but the number of GPUs (~1–4) was smaller than the number of parallel processes. Therefore, it was computationally more efficient to “bundle” variance optimization tasks from multiple processes and launch them in parallel on the GPU. For this, we implemented a thread that collected variance optimization tasks from different processes, waited until at least 20 tasks (up to 200 tasks) accumulated or a timeout had passed (10 s; which rarely happened in practice) and launched l these tasks on a GPU in parallel.

To launch R variance optimization tasks in parallel, each task 1≤r≤R with $I_{r}=\ell_{r}\cdot s_{r}$ summands, we combined their arrays into a joint array of total length $\sum_{r=1}^{R}I_{r}$

$$\underset{\text{task}\;1}{\underbrace{n_{1}^{\left(1\right)},\ldots ,n_{I_{1}}^{\left(1\right)}}},\ldots ,\underset{\text{task}\;r}{\underbrace{n_{1}^{\left(r\right)},\ldots ,n_{I_{r}}^{\left(r\right)}}},\ldots ,\underset{\text{task}\;R}{\underbrace{n_{1}^{\left(R\right)},\ldots ,n_{I_{R}}^{\left(R\right)}}}$$

(similar combined arrays for weights $\omega_{i}^{\left(r\right)}$ and predictions $\theta_{i}^{\left(r\right)}$).

We copied these combined arrays from the host memory to the GPU and launched a CUDA kernel with 1000⋅R threads. In thread r⋅1000+k, with 1≤r≤R and 1≤k≤1000, we then set ν=k/100 and included array positions $\sum_{a=1}^{r-1}I_{a}+1$ to $\sum_{a=1}^{r}I_{a}$ in the computation (i.e., counts $n_{1}^{\left(r\right)},\ldots ,n_{I_{r}}^{\left(r\right)}$; weights $\omega_{1}^{\left(r\right)},\ldots ,\omega_{I_{r}}^{\left(r\right)}$, and predictions $\theta_{1}^{\left(r\right)},\ldots ,\theta_{I_{r}}^{\left(r\right)}$). Cumulative lengths $\sum_{a=1}^{r}I_{a}$ were copied as an additional array from the host memory to the GPU to accurately partition these computations between tasks.

After task r⋅1000+k was completed, we wrote its result to position r⋅1000+k within an output array o of length 1000⋅R, and copied o from the GPU back to the host memory. We then returned 1000 likelihoods to each variance optimization process (likelihoods of 1000 candidate ν). Within each process, we then selected the optimized variance from these likelihoods.

###### Protein structure-based characterization of significantly mutated regions

We downloaded protein structures of cancer genes (pdb files) from the AlphaFold Protein Structure Database^14,72^ (https://ftp.ebi.ac.uk/pub/databases/alphafold/latest/UP000005640_9606_HUMAN_v4.tar for the entire human proteome; https://alphafold.ebi.ac.uk/entry/for individual pdb files). These structure predictions were generated with AlphaFold2 for nearly the entire human proteome.^14,72^ To color protein structures based on significantly mutated regions and selected functional domains (e.g., DNA-binding domains in transcription factors), we set the b-factor of amino acids to 1.0 (for those in significantly mutated regions), 2.0 (for non-significant regions that are part of the selected domain), and 0.0 (for non-significant regions outside domains). We used these b-factor values to color protein structures (see below).

To integrate experimentally solved structures into our website (oncofold.org), we further downloaded PDB files from the PDBe-KB database^73,74^ on December 15, 2025 (https://www.ebi.ac.uk/pdbe/pdbe-kb/). For each gene, we selected a representative structure by prioritizing those that 1) contained no mutated or modified residues, 2) were not part of a complex or bound to ligands, and 3) covered the largest fraction of the protein sequence. We set their colors based on significantly mutated regions in the same way as for AlphaFold-predicted structures.

####### Modeling of ligands into protein structures

For some genes, it was helpful to visualize them together with ligands (e.g., peptides, small molecules) for characterizing their function (e.g., significantly mutated regions facing interaction sites, such as GTP for GTPases, DNA for transcription factors, substrates for catalytic sites). These ligands were not part of the original AlphaFold structures, so we downloaded experimental crystal structures from the Protein DataBank (https://www.rcsb.org) that co-crystallized these ligands together with the gene of interest. Typically, these experimental crystal structures only included a subdomain of the gene of interest, so we had to combine both files to visualize both structures together.

However, the two files could not be combined directly as they typically use different coordinate systems. We used the Kabsch algorithm^91^ to transform the coordinate system of the experimental pdb file into that of the AlphaFold structure. For this, we determined amino acid residues that were part of both the AlphaFold and experimental crystal structure pdb files (*n* residues) and used the Kabsch algorithm to find a rotation matrix that minimized the distance between the coordinates of the corresponding residues in the AlphaFold and the rotated experimental crystal structure file.

In brief, let A and B denote the n×3 matrices of 3D coordinates of the n amino acids that were part of both the AlphaFold and experimental crystal structure file, respectively. To find an optimal transformation from B into A, we identify the centroids $a,b\in R^{3}$ of these matrices as

$$a_{j}={avg}_{1\leq i\leq n}A_{i,j}\;\text{and}\;b_{j}={avg}_{1\leq i\leq n}B_{ij},\;\text{for}\;1\leq j\leq 3$$

We then center A,B on their centroids and define n×3 matrices $\overset{‾}{A},\overset{‾}{B}\in R^{n\times 3}$ as

$$\begin{array}{c} {\overset{‾}{A}}_{i,j}=A_{i,j}-a_{j}\;\text{and}\;{\overset{‾}{B}}_{i,j}=B_{i,j}-b_{j}\\ \;\text{for}\;1\leq i\leq n\;\text{and}\;1\leq j\leq 3. \end{array}$$

We then determine their 3×3 cross-covariance matrix as $H={\overset{‾}{B}}^{T}\cdot \overset{‾}{A}$, followed by performing a singular value decomposition of H as $H=U\cdot S\cdot V^{T}$, with U,V orthogonal and S diagonal.

Based on this decomposition, we define the optimized rotation matrix R as

$$\begin{array}{ll} \;R=U\cdot V^{T} & \text{if}\;det\left(U\cdot V^{T}\right)=1\;\text{(no}\;\text{reflection)}\\ R=U\cdot \left(\begin{array}{ccc} & & \;\;\\ 0 & 1 & 0\\ 0 & 0 & -1 \end{array}\right)\cdot V^{T} & \text{if}\;det\left(U\cdot V^{T}\right)=-1\;\text{(reflection)} \end{array}$$

as well as the translation vector as $t=-R^{T}\cdot b+a$.

We used the translation vector t and rotation matrix R to transform the 3D coordinates of the ligand from the experimental crystal structure file into the coordinate system of the AlphaFold file. We then merged these transformed coordinates with the AlphaFold pdb file, thereby obtaining a structure file of the full gene and its ligand. We labeled the AlphaFold protein structure as chain “A”, small molecules as chain “C” (e.g., GTP), and larger ligands as chains “L”, “M”, “N”, etc. (e.g., DNA).

####### Visualization and coloring of protein structures

We loaded this combined pdb file into ChimeraX^83^ (version 1.8; 2024–06-10; downloaded from https://www.cgl.ucsf.edu/chimerax/download.html) and executed the following commands in ChimeraX to visualize protein structures and color them based on significantly mutated regions and selected domains.

set bgColor white

graphics silhouettes true width 3

select/A

hide sel surfaces

hide sel atoms

cartoon style width 3 thickness 1.5

select/A@@bfactor = 1.0

color sel #D8AA00

select/A@@bfactor = 2.0

color sel #63A4E5

select/A@@bfactor = 0.0

color sel #B4B4B4

select/L

hide sel surfaces

color sel #FF0000

(we repeated these 3 commands for additional chains in the ligand [e.g., *select/M*, *select/N*, etc.])

select/C

hide sel surfaces

style sel ball

color sel #FF0000

lighting default

select clear

surface cap false

That way, significantly mutated regions were colored in orange, domains were colored in blue, ligands were colored in red, and other parts of the gene were colored in light gray.

####### Characterizing the spatial distribution of mutations based on AlphaFold structures

We derived several metrics from AlphaFold structures to characterize the spatial distribution of mutations, including their 3D distance, structural stability, and other features in protein structures. We systematically evaluated associations between these metrics and significantly mutated regions, thereby identifying the structural features that influenced the spatial distribution of mutations for each gene. Based on these associations, we arranged genes into structurally coherent subgroups and distinguished oncogenes vs. tumor suppressors.

######## 1. 3D distance of mutations:

To characterize the spatial distribution of significantly mutated regions, we analyzed their 3D geometry in AlphaFold protein structures. We denote by ${\vec{r}}_{i}\in R^{3}$ the coordinates of the amino acid at position i∈{1,…,ℓ}. We define the centroid of each state j∈{1,…,s} as $\vec{\mu }={avg}_{i\in \{1,\ldots ,\ell \}|{\overset{ˆ}{\omega }}_{i}=j}{\vec{r}}_{i}$. We centered coordinates of residues in state j on their centroids ${\vec{r}}_{i}-\vec{\mu }$ and computed the 3×3 covariance matrix $S\in R^{3\times 3}$ as

$$S={avg}_{i\in \{1,\ldots ,\ell \}|{\overset{ˆ}{\omega }}_{i}=j}\left({\vec{r}}_{i}-\vec{\mu }\right){\left({\vec{r}}_{i}-\vec{\mu }\right)}^{T}$$

We then performed an eigen decomposition of S as $S\cdot {\vec{v}}^{\left(k\right)}=\lambda^{\left(k\right)}\cdot {\vec{v}}^{\left(k\right)}$, where $\lambda^{\left(1\right)}\leq \lambda^{\left(2\right)}\leq \lambda^{\left(3\right)}$. We denoted by ${\vec{v}}_{\text{max}}={\vec{v}}^{\left(3\right)}$ the eigenvector with the largest eigenvalue, representing the main axis of the spatial distribution of residues in state s. We further identified eigenvector ${\vec{v}}_{\text{min}}={\vec{v}}^{\left(1\right)}$ with the smallest eigenvalue, representing the vector orthogonal to the best-fitting plane of residues in state s.

Using these vectors, we defined the following 3D distance metrics for each position i∈{1,…,ℓ}:

Euclidean distance to center:

$$d_{dot}(i)=\left\|{\vec{r}}_{i}-\vec{\mu }\right\|$$

Perpendicular distance to the principal axis:

$$d_{\text{line}}(i)=\left\|{\vec{v}}_{max}\times \left({\vec{r}}_{i}-\vec{\mu }\right)\right\|/\left\|{\vec{v}}_{max}\right\|$$

Distance to the best-fit hyperplane:

$$d_{\text{plane}}(i)={\left({\vec{r}}_{i}-\vec{\mu }\right)}^{T}{\vec{v}}_{\text{min}}/\left\|{\vec{v}}_{\text{min}}\right\|$$

where ‖⋅‖ denotes the Euclidean distance and $\vec{v}\times \vec{w}$ the cross product of vectors $\vec{v},\vec{w}$.

If a ligand or small-molecule structure was available, we additionally defined.

Distance to the nearest ligand atom:

$$d_{\text{ligand}}(i)={min}_{k}\;\left\|{\vec{r}}_{i}-{\vec{p}}_{k}\right\|$$

where ${\vec{p}}_{k}$ denotes the 3D coordinates of atoms in the ligand or small molecule structure.

We plotted $d_{\text{dot}}(i),d_{\text{line}}(i),d_{\text{plane}}(i)$, and $d_{\text{ligand}}(i)$ as a function of position i∈{1,…,ℓ} and examined which of these metrics was most aligned with the spatial distribution of residues in state j. For example, alignment with $d_{\text{dot}}(i)$ indicated that mutations clustered around a specific spot (e.g., a binding pocket or binding site of a small molecule). Alignment with $d_{\text{line}}(i)$ suggested that mutated residues stretch across a longer structure (e.g., protein-DNA interactions or peptide binding grooves). $d_{\text{plane}}(i)$ was typically most aligned if the surface of an entire part of a protein was mutated (e.g., large catalytic domains or protein-protein interaction sites).

######## 2. Stability and regulatory tails of the protein structure:

We quantified the stability stab (i) of each position i∈{1,…,ℓ} in the protein structure based on mutation-induced changes in protein thermodynamic stability (ΔΔG). To estimate ΔΔG, we ran the “Position-Scan” module in the FoldX Suite ^53^ (version 5.1), testing all possible amino acid substitutions (option “a”) at each residue. For each residue, we calculated the average ΔΔG across the 19 non-self substitutions after capping individual ΔΔG values to the range of −5 to 5 kcal/mol to reduce the influence of extreme outliers.

To identify intrinsically disordered regions, we used the pLDDT score,^14^ which evaluates the local distance between atoms and quantifies their stereochemical plausibility. A high pLDDT indicates that the residue is part of a stable structure that can be predicted with high confidence. By contrast, a low pLDDT often indicates that the residue is part of a region that is highly flexible or intrinsically disordered without a well-defined structure. We extracted pLDDT scores from the b-factor column of the AlphaFold pdb files (AlphaFold provides these scores to evaluate the regional confidence score of its model).^72^ We further used this metric to define intrinsically disordered N- and C-terminal tails of protein structures as residues $\left\{1,\ldots ,k_{1}\right\}$ and $\left\{k_{2},\ldots ,\ell \right\}$ with $stab(i)<0.8\;\forall i\in \{1,\;\left.\ldots ,k_{1}\right\}\cup \left\{k_{2},\ldots ,\ell \right\}$.

######## 3. Other proteomics variables:

We used the DSSP algorithm^84,85^ to characterize the secondary protein structure of each amino acid residue i∈{1,…,ℓ}, including its solvent accessibility (i.e., whether amino acids are located at the surface or buried within the core of the protein structure), their rotation around N-Cα and Cα-C bonds, secondary structure (e.g., alpha-helices or beta-sheets), and energy of hydrogen bonds.

To run the dssp algorithm, we installed the Biopython library^86^ (version 1.81) using the command line.

pip install biopython

We further installed the DSSP executable command mkdssp (version 0.9.9).

sudo apt-get install dssp (on Ubuntu)

brew install brewsci/bio/dssp (on MacOS)

(see installation instructions of dssp on https://ssbio.readthedocs.io/en/latest/instructions/dssp.html).

In Python 3 (version 3.11.3), we then executed the following commands to run the DSSP algorithm^84,85^

from Bio.PDB import PDBParser

from Bio.PDB.DSSP import DSSP

pdb_filename=<path to pdb file>

structure = PDBParser().get_structure(“protein”, pdb_filename)

dssp = DSSP(model = structure[0], in_file = pdb_filename, dssp = ’mkdssp’,file_type = ”pdb”)

where we specified the path to the pdb file(s) from AlphaFold (e.g., AF-P30281-F1-model_v4.pdb).

We then extracted the values from the dssp object using the following commands in Python.

for chain in structure.get_chains():

for residue in chain:

dssp_key = (chain.get_id(), residue.get_id())

print (dssp[dssp_key])

That way, we obtained several variables for every residue characterizing its protein structure, including secondary structure including alpha-helices (H), beta-sheets (E), etc.

relative SASA the solvent-accessible surface area (SASA) of a residue, normalized by the theoretical maximum possible SASA for each amino acid

phi the rotation around the N-Cα bond (torsional angle of amino acids)

psi the rotation around the Cα-C bond (torsional angle of amino acids)

nh_o1_energy the energy of the hydrogen bond from the backbone NH to the first acceptor O

o_nh1_energy the energy of the hydrogen bond from the first donor NH to the backbone C=O

nh_o2_energy the energy of a second hydrogen bond from NH to another O

o_nh2_energy the energy of a second hydrogen bond to C=O from another NH.

We tested the spatial alignment of each structural variable with significantly mutated regions. For example, alignment with SASA indicated that mutations primarily targeted the surface vs. the interior core of a protein.

######## 4. Evaluation of association with significantly mutated regions:

We evaluated statistical significance of associations between significantly mutated regions and structural features using a random permutation test. For each protein, we randomly selected the same number of regions, each matched in length to the corresponding significantly mutated region, 10,000 times. To weight the metrics with mutation density, we computed the normalized mutation count for residue i within significant regions as

$$c_{i}=\frac{\sum_{j\in \{1,\ldots ,t\}}n_{i,j}}{\sum_{j\in \{1,\ldots ,t\}}m_{i,j}}$$

where $n_{i,j}$ denotes the observed number of mutations and $m_{i,j}$ the context-dependent mutation rate for each amino acid substitution j∈{1,…,t}.

For each permutation, we then calculated the following weighted metrics for secondary structures, structure-derived metrics (ΔΔG, pLDDT score, and relative SASA), pairwise distances, and 3D distance metrics.

Weighted fraction of overlap with secondary structures:

$$f=\sum_{\omega_{i}=s}c_{i}/\sum c_{i}$$

Weighted average of structure-derived metrics:

$$f=\sum c_{i}\alpha_{i}/\sum c_{i}$$

Weighted average of pairwise distances:

$$f=\sum \;d_{i,j}\left(c_{i}+c_{j}\right)/\sum \;\left(c_{i}+c_{j}\right)$$

Weighted average of Euclidean distance to center:

$$f=\sum c_{i}d_{dot}(i)/\sum c_{i}$$

Weighted average of perpendicular distance to the principal axis:

$$f=\sum c_{i}d_{\text{line}}(i)/\sum c_{i}$$

Weighted average of distance to the best-fit hyperplane:

$$f=\sum c_{i}\left|d_{\text{plane}}(i)\right|/\sum c_{i}$$

where $\omega_{i}=s$ indicates that residue i is assigned to secondary structure $s,\alpha_{i}$ denotes the structure-derived metric of each residue i, and $d_{i,j}$ denotes the Euclidean distance between residues i and j.

In each permutation, we additionally shuffled the normalized mutation counts across residues to disrupt any inherent relationship between structural features and specific residue positions. We then estimated one-sided *p*-values from the resulting empirical distributions. To avoid spurious associations, we restricted the analysis to genes for which the average normalized mutation count in significant regions was greater than that in non-significant regions and at least three residues were included in significantly mutated regions.

######## 5. Classification of oncogenes and tumor suppressors:

To generate a model to classify cancer genes as oncogenes or tumor suppressors based on their associations with structural features, we used log-transformed one-sided *p*-values derived from permutation tests, with the sign determined by the direction of the association. These signed log *p*-values were used as input features in a logistic regression model with LASSO regularization, implemented using the “lassoglm” function in the Statistics and Machine Learning Toolbox (version 24.1) of MATLAB R2024a. To estimate deviance, we performed 10-fold cross-validation with 100 Monte Carlo repetitions and determined lambda for this model as the value with minimum deviance. To ensure robust model training, we restricted the training set to 56 oncogenes or tumor suppressors annotated in the Cancer Gene Census (CGC) that had 1) a ratio of normalized mutation counts between significant and non-significant regions >2 and 2) an average number of mutations per residue >1.5. We subsequently evaluated the regression model on genes not annotated as oncogenes or tumor suppressors in CGC, as well as genes annotated as both categories, if they had a ratio of normalized mutation counts between significant and non-significant regions greater than 1.

###### Biological follow-up analyses of significantly mutated regions

We performed multiple follow-up analyses to assess the biological validity of significantly mutated regions in cancer genes.

####### Quantification of mutational excess in significantly mutated regions

To verify that mutations accumulated in the significant regions identified by our model, we compared the excess of mutations in significant vs. non-significant regions within cancer genes. To quantify the mutational excess of regions, we used a metric similar to the dN/dS ratio.^92^ Because synonymous mutations were not reported in GENIE,^12,13^ we modified the ratio to use missense mutations with AlphaMissense^15^ scores $e_{i}<0.1$ as a marker of the background mutation rate, assuming these mutations were mostly benign.

We first computed the background mutation rate $r_{b}$ in benign positions

$$r_{b}=\frac{\sum_{i\in \{1,\ldots ,\ell \}|e_{i}<0.1}n_{i}}{\sum_{i\in \{1,\ldots ,\ell \}|e_{i}<0.1}m_{i}}$$

where $n_{i}$ denotes the observed number of mutations and $m_{i}$ the context-dependent mutation rate of position i. We further determined for each position i∈{1,…,ℓ} its most significant state ${\hat{\omega }}_{i}=\underset{j\in \{1,\ldots ,s\}}{arg\;max}\omega_{i,j}$ and computed the mutation rate of each state s∈{1,…,s} in non-benign positions as

$$r_{j}=\frac{\sum_{{\overset{ˆ}{\omega }}_{i}=j|e_{i}>0.98}n_{i}}{\sum_{{\overset{ˆ}{\omega }}_{i}=j|e_{i}>0.98}m_{i}}$$

(we repeated the analysis for different cutoffs of $e_{i}$).

We then compared $r_{j}$ with the background mutation rate $r_{b}$ and identified states with mutational excess as $S_{\text{sel}}=\left\{j\in \{1,\ldots ,s\}|r_{j}/r_{b}\geq \right.\;2.0\}$. We then determined the total number of mutations $n_{\text{sel}}=\sum_{{\overset{ˆ}{\omega }}_{i}\in S_{\text{sel}}|e_{i}>0.98}n_{i}$ and background mutability $m_{\text{sel}}=\sum_{{\overset{ˆ}{\omega }}_{i}\in S_{\text{sel}}|e_{i}>0.98}m_{i}$ of these states.

To assess whether these counts significantly exceeded those expected by chance, we determined a p-value

$$p_{\text{sel}}=P\left(Y_{PLN}\geq n_{\text{sel}}|r_{b}\cdot m_{\text{sel}},\nu \right)$$

as the upper tail of a Poisson-lognormal distribution with distribution mean $r_{b}\cdot m_{\text{sel}}$ and ν from the Hidden Markov model, signifying whether the observed number of mutations exceeded that expected by chance.

Analogously, we defined $S_{\text{non-sel}}=\left\{j\in \{1,\ldots ,s\}|r_{j}/r_{b}<2.0\right\},n_{\text{non-sel}}=\sum_{{\overset{ˆ}{\omega }}_{i}\in S_{\text{non-sel}}|e_{i}>0.98}n_{i}$, and $m_{\text{non-sel}}=\sum_{{\overset{ˆ}{\omega }}_{i}\in S_{\text{non-sel}}|e_{i}>0.98}m_{i}$ for the remaining states in the cancer gene, with

$$p_{\text{non-sel}}=P\left(Y_{\text{PLN}}\geq n_{\text{non-sel}}|r_{b}\cdot m_{\text{non-sel}},\nu \right)$$

We then compared $p_{\text{sel}}$ and $p_{\text{non-sel}}$ for each cancer gene, quantifying whether the mutational excess signal accumulated within the significantly mutated regions identified by our model vs. the rest of the gene.

####### Enrichment of functionally annotated residues in significantly mutated regions

To characterize the functional relevance of significantly mutated regions, we obtained residue-level functional annotations from InterPro database^75^ (downloaded protein2ipr.dat.gz from https://www.ebi.ac.uk/interpro/download/ on October 07, 2025). We used residues annotated as “Domain” or “Homologous_superfamily” to calculate the fraction of functionally annotated residues within significant and non-significant regions. To reduce spurious effects, the analysis was restricted to genes containing at least one significant region composed of more than two amino acid residues. The enrichment of functional annotations in significant regions was then evaluated by comparing these fractions against those of non-significant regions.

####### Cancer type enrichment of significantly mutated regions

We assessed cancer type enrichment of individual significantly mutated regions for genes harboring multiple significant regions in the overall cohort. First, we counted the number of patients with mutations in each discrete significant region for each cancer type. For each region-cancer pair, we performed a one-sided Fisher’s exact test comparing the number of patients within a given cancer type who harbored mutations in the region of interest to patients from other cancer types or patients with mutations outside the region. We adjusted the resulting *p*-values for multiple comparisons using the Benjamini-Hochberg method to obtain false discovery rates (FDRs). To reduce spurious associations, we performed the tests only when the following criteria were satisfied: 1) the number of patients within the cancer type ≥50, 2) the number of patients with mutations in the region ≥50, and 3) the expected frequency $E=\frac{\left(\mathrm{Row}\;\mathrm{total}\times \mathrm{Column}\;\mathrm{total}\right)}{\mathrm{Grand}\;\mathrm{total}}$ in all cells of the contingency table ≥3. Finally, we identified significant region-cancer pairs as those with FDR <0.01 and odds ratio >2. We next evaluated whether significantly mutated regions within the same gene showed differing cancer type-specific distributions. For each gene-cancer pair, we calculated the fraction of significant regions showing significant cancer type enrichment (i.e., significant region-cancer pairs) among the total number of significant regions within that gene. We identified 104 gene-cancer pairs with a fraction <1, indicating the presence of cancer type-specific mutated regions within those genes.

####### Associations between significantly mutated regions and nonsense-mediated decay and alternative translation initiation

To connect nonsense-mediated decay (NMD) escape regions to the mutation patterns captured by our model, we applied established rules to predict NMD escape^93^: truncations are predicted to escape NMD if they occur in the last exon or within 50 nucleotides upstream of the final exon-exon junction (canonical rules). We applied these rules to the representative transcript of each gene and annotated residues located within predicted NMD escape regions. For visualization (Figure S10A), we additionally annotated NMD escape regions using three rules^94^: truncations occurring within the first 150 nucleotides of the coding sequence, within an exon spanning more than 407 nucleotides, or within intronless transcripts are predicted to escape NMD.

To predict regions associated with alternative translation initiation (ATI), we identified downstream start codons near the N-terminus that could function as alternative start codons, using NetStart 2.0^87^ (accessed on October 24, 2025; https://services.healthtech.dtu.dk/services/NetStart-2.0/) and TIS Transformer^88^ implemented in the TRISTAN suite (version 1.0.0; https://github.com/TRISTAN-ORF/TRISTAN). We applied probability thresholds of 0.625 for NetStart 2.0 and 0.05 for TIS Transformer, following the optimal universal thresholds reported previously.^87^ If the predicted alternative start codons from the two methods were inconsistent, we selected the more downstream alternative start codon identified by either method as the representative one. To ensure that proteins produced via ATI would retain substantial functional consequences, we restricted the analysis to genes for which the predicted alternative start codon was located within the first half of the coding sequence and left at least 100 residues downstream of the site.

To quantify the depletion of significantly mutated regions within NMD escape regions and ATI regions (regions between conventional and alternative translation initiation sites), we calculated the weighted fraction of overlap with NMD sensitive regions (residues not included in predicted NMD escape regions) and non-ATI regions (residues not included in predicted ATI regions), respectively, as

$$f=\frac{\sum_{\omega_{i}=s}c_{i}}{\sum c_{i}}$$

where $\omega_{i}=s$ indicates that residue i is assigned to NMD sensitive region or non-ATI region s, and $c_{i}$ represents the normalized mutation count of residue i.

We evaluated statistical significance of associations between significantly mutated regions and NMD/ATI using the same procedure described in section 3.3.4.

####### Evaluation of functional effects of mutations using mutagenesis screening data

We assessed whether the computational predictions of our model captured regions with a high density of functionally relevant mutations identified through experimental assays in less-well-studied cancer genes and domains. To this end, we compiled experimental mutagenesis screening data for *KRAS*,^39^ *POT1*,^37^ and *PPM1D*^38^ from previous studies. For *KRAS*, we used gain-of-function scores for 3,536 missense mutations spanning amino acid residues 2–188, calculated as log_2_-fold-changes based on the average allele representation before and after transducing a mutation screening library into HA1E cells (an immortalized human kidney epithelial cell line) expressing wild-type *KRAS* (“HA1E_WT_LFC” column in Extended Data Table S1). For *POT1*, we used log_2_-fold-changes in allele frequency measured between one vs. three weeks after introducing mutations into POT1 locally haploid cells derived from human embryonic stem cells (“mean_wk3_log2fc” column in Table S1). We included 1,140 missense mutations annotated as “Alanine” or “Substitution” covering 517 amino acid residues. For *PPM1D*, we used log_2_-fold-changes in single guide RNA abundance in Molm13 cells (a human acute myeloid leukemia cell line) measured after 12 days of treatment with cytarabine (chemotherapy agent) compared to vehicle (DMSO) (“AraC-DMSO Repl123 Day12 LFC” column in Table S5). We included 466 truncating mutations spanning 317 residues. To visualize log_2_-fold-changes as functional scores in a consistent direction (y axis of Figure S10E–S10G), we negated the log_2_-fold-change values for *POT1*, as negative log_2_-fold-changes in this assay indicate deleterious effects on cell growth.

To evaluate whether significantly mutated regions can help prioritize oncogenic mutations with limited database support (Figure S26D), we further collected public experimental mutagenesis screening data for *TP53*,^60^ *VHL*,^61^ and *PTEN*.^62^ For *TP53*, we used z-scores for missense mutations, measuring differences in allele frequency in library-infected p53^WT^ A549 cells (a human lung adenocarcinoma cell line) after 12 days of nutlin-3 (an agent interrupting the interaction of p53 with MDM2, a negative regulator of p53 stability and transcriptional function) treatment compared to early time points (“A549_p53WT_Nutlin-3_*Z* score” column in Table S3). For *VHL*, we used function scores across missense mutations, estimated from the log_2_-ratio between day 6 vs. day 20 after transfection of saturation genome editing libraries into HAP1 cells (a haploid human cell line) (“function_score_final” column in Table S1). For *PTEN*, we used cumulative fitness scores for missense mutations, estimated from read counts in mutant samples with error correction based on sequencing of wild-type samples in an artificial humanized yeast model (“Cum_score” column in Table S2).

####### Benchmarking of significantly mutated regions against literature-curated mutations

We compared significantly mutated regions from our model with cancer-relevant mutations reported in the literature. For this, we first identified the most significant state ${\hat{\omega }}_{i}=\underset{j\in \{1,\ldots ,s\}}{arg\;max}\omega_{i,j}$ of each position i∈{1,…,ℓ}, and we computed the emission rate from our model as

$$f_{{\overset{ˆ}{\omega }}_{i}}\left(e_{i}\right)=\theta_{i,{\overset{ˆ}{\omega }}_{i}}/m_{i}$$

where $f_{j}(\cdot )$ is the mapping function of state $j,e_{i}$ is its predicted effect, $m_{i}$ is the background mutation rate of position i, and $\theta_{i,j_{i}}$ is the predicted count from our model in state j. The emission rate $f_{{\overset{ˆ}{\omega }}_{i}}\left(e_{i}\right)$ quantified whether the Hidden Markov model considered the position as significantly mutated (i.e., $f_{{\overset{ˆ}{\omega }}_{i}}\left(e_{i}\right)\gg 1$) or part of the background distribution (i.e., $f_{{\overset{ˆ}{\omega }}_{i}}\left(e_{i}\right)\approx 1$).

To benchmark the emission rates of our model against literature-based annotations, we downloaded the COSMIC Mutation Census^48^ (v100/Hg19; cmc_export.tsv on https://cancer.sanger.ac.uk/cmc/home), including all missense and nonsense SNVs from all COSMIC tiers 1–3 (not labeled as “Other”). As an orthogonal reference, we further obtained data from the ClinVar database^50^ (GRCh37; downloaded January 13, 2026; variant_summary.txt.gz on https://www.ncbi.nlm.nih.gov/clinvar/docs/downloads/), including all missense and nonsense SNVs labeled as “somatic” and annotated as “Pathogenic”, “Likely pathogenic”, or “drug response”. In addition, we downloaded the CIViC database^49^ (version CIViCpy_v5.1.0/Hg19; January 01, 2026; “Accepted Variants VCF” file on https://civicdb.org/releases/main), including all missense and stop-gained SNVs annotated as “SOMATIC” with supporting evidence (typically in the form of a literature reference).

We then sorted mutations based on the emission rates from our model, and we gradually counted the number of mutations $c_{i}$ in COSMIC, ClinVar, and CIViC databases. We normalized these counts based on the total number of mutations C in COSMIC, ClinVar, and CIViC, respectively, and plotted ROC curves $c_{i}/C$ against their sorted indices i (ranging from 1 to the total number of positions included in the analysis). We excluded genes with partial annotation of mutations across their coding sequence (e.g., mutations are annotated only in certain regions of a gene) or with transcript models inconsistent with our genomic dataset. Accordingly, for each database, we restricted the analysis to genes with at least five annotated mutations and matching transcript IDs, resulting in a total of 91, 30, and 14 genes for COSMIC, ClinVar, and CIViC, respectively. Enrichment of the emission rates for literature-curated COSMIC, ClinVar, and CIViC mutations indicated the ability of our model score to prioritize biologically relevant mutations.

To contextualize the performance and added value of our model, we repeated the analysis using three previously reported methods: HotSpot3D,^54^ OncodriveCLUSTL,^55^ and ActiveDriver.^56^ We applied HotSpot3D (version 0.6.0) to the filtered missense SNVs from the AACR Project GENIE dataset (see method details above for filtering criteria), using default parameters and proximity data downloaded from synapse.org(https://www.synapse.org/Synapse:syn9705109). Mutations were ranked based on closeness centrality and significantly mutated regions were defined using a cluster closeness threshold corresponding to the top 5% of clusters across all genes. We also applied OncodriveCLUSTL via its web interface (accessed on January 16, 2026; https://bbglab.irbbarcelona.org/oncodriveclustl/analysis) to the same filtered missense SNVs, using default parameters. We ranked mutations according to the resulting *p*-values and identified significantly mutated regions as residues containing at least one genomic position with *p*-value <0.05. Finally, we ran ActiveDriver (version 1.0.0), using protein sequences and post-translational modification sites downloaded from ActiveDriverDB^95^ (https://activedriverdb.org/download/) on January 8, 2026. Significantly mutated regions were defined using a region-level *p*-value <0.05. For mutation-level ranking, the corresponding region-level *p*-value was assigned to each mutation within that region. As a reference, we annotated mutations using multiple orthogonal metrics. We incorporated predicted pathogenicity using AlphaMissense scores^15^ (see method details above), deleteriousness using PHRED-normalized CADD scores^51^ (v1.6/Hg19; whole_genome_SNVs.tsv.gz on https://cadd.gs.washington.edu/download), and evolutionary conservation using PhyloP 100-way vertebrate alignment scores^52^ downloaded from dbNSFP v4.^76^ We further estimated the predicted effect on protein stability (ΔΔG) using the “PositionScan” module in the FoldX Suite^53^ (version 5.1), testing all possible amino acid substitutions (option “a”) at each residue. We repeated the COSMIC, ClinVar, and CIViC enrichment analyses by sorting mutations based on each of these metrics.

We performed the analysis at both the residue and genomic position levels (Figures 5A and S20A–S20C). The residue-level analysis enabled comparison across all methods and metrics, as all methods and metrics provide residue-level *p*-values or scores. The genomic position-level analysis allowed evaluation at higher resolution and generally demonstrated superior performance. For methods and metrics providing *p*-values or scores at the genomic position or amino acid substitution level, we summarized values to the residue level by taking the minimum *p*-value or maximum score. Since ActiveDriver and FoldX do not provide *p*-values or scores at individual genomic positions, they were excluded from the genomic position-level analysis.

####### Multi-omics data for biological and clinical follow-up of significantly mutated regions

To complement literature-based annotations, we leveraged three independent datasets to assess the biological and clinical impact of mutations on expression (mRNA, protein, phosphopeptide), patient survival, and clinical therapy response.

######## 1. Hartwig Medical Foundation (HMF):

We obtained somatic mutation calls under controlled data access (DR-391).^2,77^ We included all mutations annotated as *“All filters passed”* in the VCF files from the HMF tools Purple v3.9 and Sage v3.2.3. For patients with multiple tumor samples, we prioritized those with matched RNA-seq data and the highest number of somatic mutations. We included all coding variants and classified them based on the annotation in “IMPACT” (missense_variant, stop_gained, stop_lost, start_lost, frameshift_variant, inframe_insertion, inframe_deletion, splice_donor_variant, splice_acceptor_variant). We also obtained matched mRNA expression data under DR-391, and used adjusted TPM values from the Isofox output, followed by log-transformation for our analyses.

######## 2. The Cancer Genome Atlas (TCGA):

We downloaded somatic variants from the GDC portal (https://portal.gdc.cancer.gov/) on Jun 24, 2024 as wxs.aliquot_ensemble_masked.maf files. If a patient had multiple samples, we followed the same criteria as for the HMF dataset. We classified coding mutations based on the “Variant_Classification” field (Missense_Mutation, Nonsense_Mutation, Nonstop_Mutation, Translation_Start_Site, Frame_Shift_Ins, Frame_Shift_Del, In_Frame_Ins, In_Frame_Del, Splice_Site). We further downloaded matched mRNA expression data as rna_seq.augmented_star_gene_counts.tsv files from the GDC portal on Jun 17, 2024, and used gene-level FPKM values, followed by log-transformation.

######## 3. Clinical Proteomic Tumor Analysis Consortium (CPTAC):

We downloaded somatic mutation calls processed by the Baylor College of Medicine (BCM) pipeline (Mutation_BCM_v1.zip) from the PDC portal (https://pdc.cancer.gov/pdc/cptac-pancancer) on Jun 11, 2024.^78,79^ We classified coding mutations using the same criteria as for the TCGA dataset. We further downloaded matched mRNA, protein, and phospho-peptide data (Proteome_BCM_GENCODE_v34_harmonized_v1.zip, RNA_BCM_v1.zip, and Phosphoproteome_BCM_ GENCODE_v34_harmonized_v1.zip) from the PDC portal on Jun 11, 2024. For RNA-seq data, we used gene-level log-transformed, upper quartile-normalized FPKM values from tumor samples. We used gene-level (protein) or peptide-level (phosphopeptide) log-fold-change values from tumor samples, normalized to reference channel intensity.

To normalize expression data between cancer types in these three datasets, we first auto-scaled the log-transformed expression values of each gene within each cancer type, followed by quantile normalization across all samples within each dataset. For analyses conducted separately within individual cancer types, we applied quantile normalization to the auto-scaled values across all samples within each cancer type.

####### Systematic comparison of the impact of mutations in significant vs. non-significant regions

We applied 6 orthogonal criteria to these datasets to assess the biological and clinical impact of mutations in cancer genes and compare the effects of mutations in significant vs. non-significant regions.

######## 1. Differential expression of mutated genes:

To quantify the differential expression of genes, we calculated the ratio of the median expression in mutated vs. non-mutated samples (x-fold expression). To avoid spurious associations resulting from a low number of mutations, we divided the total number of mutations by the number of amino acids and excluded genes with rates below 1.5 (missense mutations) and 0.5 (truncating mutations). Furthermore, we only included genes for which matched expression and mutation data were available for at least 3 independent patients in both significantly and non-significantly mutated regions.

######## 2. Effect on transcriptional activity:

For mutations in transcription factors, we compared their transcriptional activity in mutated vs. non-mutated samples based on the differential expression of their target genes. We used a two-sided Wilcoxon rank-sum test (z-statistic) to compare target gene expression in mutated vs. non-mutated samples. Target genes were obtained from TFLink,^80^ comprising 16,634 small-scale interactions between 839 transcription factors and 4,680 target genes for homo sapiens.

######## 3. Changes to downstream signaling:

To probe the impact of mutations on downstream signaling, we selected for each gene the hallmark and oncogenic signature gene set in MSigDB^81^ that was the most relevant to its downstream activity (for mRNA and protein data). We further selected the most relevant PTM signature in PTMSigDB (https://github.com/nicolerg/ssGSEA2/tree/master/db/ptmsigdb)^82^ for phosphopeptide data. We then performed a gene set enrichment analysis or post-translational modification signature enrichment analysis (PTM-SEA) based on the differential expression of these signatures between mutated vs. non-mutated samples (z-statistic; two-sided Wilcoxon rank-sum test) using the ssGSEA2 package (version 1.0.0).^82^ Genes without a relevant downstream signaling signature were excluded from this analysis. The selected signatures for each gene are listed in Table S5.

######## 4. Clonality vs. subclonality of mutations:

We estimated and compared the clonality of mutations in significant vs. non-significant regions of cancer genes as follows.

######### Step 1: Raw allelic fraction.

For each somatic mutation, we calculated the raw allelic fraction as

$$f=n_{\text{alt}}/\left(n_{\text{ref}}+n_{\text{alt}}\right)$$

where $n_{\text{ref}}$ and $n_{\text{alt}}$ are the number of reads supporting the reference (i.e., non-mutated) and alternate (mutated) allele, respectively. To ensure that differences in copy number did not confound our analyses, we limited our analyses to somatic mutations in regions without copy number alterations (i.e., CNV = 0), as per the CNV data in GENIE^12,13^ (mutations without copy number information were excluded).

######### Step 2: Within-sample normalization.

To control for variability in sequencing depth and tumor content, we normalized the allelic fraction of each mutation to the most clonal event in each sample:

$$\tilde{f}=f/max\left\{f_{1},\ldots ,f_{k}\right\}$$

where $\left\{f_{1},\ldots ,f_{k}\right\}$ denotes the allelic fractions of all mutations in the same sample. This assumes the highest allelic fraction in a non-amplified region likely corresponds to a clonal heterozygous event present in all tumor cells.

######### Step 3: Normalizing to a common baseline.

To place clonality scores on a common baseline, we normalized the clonality of each mutation to the median clonality of all somatic mutations

$$\tilde{r}=\tilde{f}/median\left\{{\tilde{f}}_{i}\right\}$$

Ratios $\tilde{r}\approx 1$ indicate that the clonality is near the baseline of other somatic mutations in the dataset.

######### Step 4: Summary of clonality.

For each gene g, we computed the median clonality ratio $\tilde{r}$ across mutations with AlphaMissense scores ≥0.9 in significant $\left({\tilde{r}}_{g,\text{sig}}\right)$ and non-significant regions $\left({\tilde{r}}_{g,\text{ns}}\right)$ of g. We compared the distribution of ${\tilde{r}}_{g,\text{sig}}$ and ${\tilde{r}}_{g,\text{ns}}$ to assess the clonality in significant vs. non-significant regions.

######## 5. Association with clinical survival:

To evaluate whether mutations correlate with differences in patient survival, we collected overall survival data from HMF and TCGA. To avoid spurious associations, we excluded genes for which survival data were available for fewer than 10 patients with mutations in significant or non-significant regions.

To adjust survival times for differences between cancer types and other patient-specific variables, we used the glmnet package (version 4.1.8)^89^ to build a baseline Cox regression model with LASSO regularization that predicts survival based on the total number of coding mutations and cancer types. The optimal lambda for this model was determined via 10-fold cross-validation repeated 100 times. Final regression coefficients were obtained as the median of the coefficients from Cox regression fitted to 100 random 90% patient subsets.

Using fixed coefficients from this baseline regression model, we built a second Cox regression model that integrates both the predictors of the baseline model and the mutation status in significant and non-significant regions of each cancer gene. To account for the impact of other coding mutations in the same gene beyond the mutation type of interest (i.e., missense or truncating), we included the mutation status of all other coding mutations in the gene as an additional covariate. We followed the same Cox regression procedure with LASSO regularization as for the baseline model.

We finally quantified the effect of mutations on survival as the absolute value of the regression coefficient in the second model (hazard ratio), reflecting differences in survival that cannot be explained by differences between tumor types or other patient-specific variables. To visualize survival times, we adjusted them for tumor type-specific differences across cancer types using the ggadjustedcurves function in the survminer package (version 0.5.0) (https://github.com/kassambara/survminer).

######## 6. Association with therapy response:

To determine whether mutations correlated with differential outcomes to therapies, we collected pre-biopsy treatment information for patients in the HMF dataset (Figure S24C), including the type of therapy (e.g., anti-EGFR) and its duration (i.e., the time until the therapy was discontinued, such as due to lack of efficacy or disease progression). We only included treatments with data for at least 50 patients and gene-treatment pairs with annotated treatment data for at least 5 mutations in significant and non-significant regions of the gene. For patients who received multiple treatments with the same type of drug, we selected the longest duration to represent that treatment class. For each type of treatment, we applied the same Cox regression procedure with LASSO regularization as for the overall survival analysis to compare the efficacy in mutated vs. non-mutated patients. We adjusted treatment durations for tumor type-specific differences following the same survminer procedure as for overall survival data.

To further investigate therapy associations, we collected treatment data for patient subgroups from the AACR GENIE cohorts, available through the GENIE Biopharma Collaborative (BPC) under controlled data access in cBioPortal (accessed on March 05, 2026; https://genie.cbioportal.org/) and Synapse (ACT-3038; BrCa v1.0-public, NSCLC v2.0-public, and CRC v2.0-public; https://www.synapse.org/Synapse: syn27056700).^12,13^ For each treatment, we summarized progression-free survival (PFS) as the time to disease progression based on the later documentation of disease worsening that was recorded in both an imaging report and medical oncologist assessment, or death (PFS-I-and-M). We applied the same Cox regression procedure with LASSO regularization used in the overall survival analysis to evaluate differences in PFS by mutation status. We first constructed a baseline model using clinical covariates, including age, sex, sample type, stage at diagnosis, distant metastasis status, and cancer-specific variables (e.g., hormone receptor subtype for breast cancer and microsatellite instability status for colorectal cancer). We then incorporated deleterious mutation status (e.g., nonsense and frameshift mutations) and significance scores for missense mutations into this baseline model. To reduce spurious associations, we only included treatments with data from at least 50 patients and gene-treatment pairs with annotated PFS for at least 10 mutations in significant regions. In addition, we restricted the analysis to the genes that the average normalized mutation count in significant regions was greater than that in non-significant regions. For patients with multiple samples, we prioritized those harboring mutations in significant regions and with higher significance scores. To further reduce potential false positives, we quantified the differences in normalized area under the Kaplan-Meier curves (restricted mean survival time, RMST) comparing both low-vs. high-significance score groups and non-mutated vs. high-score groups. High- and low-score groups were defined using the 50^th^ percentile of significance scores among all patients with mutations in significant regions, with patients above the 50^th^ percentile classified as high-score and those at or below as low-score. We included only gene-treatment pairs with annotated PFS for at least 3 mutations in both high- and low-score groups. To enable comparisons across treatments with differing durations, we normalized RMST values to a 0-to-1 scale by dividing by the time to the last non-censored event in the earlier group. If no non-censored events were observed, the earliest censored time was used instead.

##### Application for harmonizing annotations of oncogenic mutations in clinical knowledge bases

We first compared the annotation of oncogenic mutations between five major knowledge bases. To ensure a fair comparison across these resources, we restricted analyses to genes annotated in at least 3 of the 5 independent databases. Across all resources, we considered only mutations corresponding to amino acid substitutions in protein-coding regions. Mutations were classified as oncogenic according to the database-specific annotation schemes. We further defined a more stringent top-tier subset of oncogenic mutations in each database.

###### COSMIC.

We downloaded COSMIC Mutation Census^48^ cmc_export.tsv from https://cancer.sanger.ac.uk/cmc/home (v100). For the top-tier analysis, only variants assigned to tiers 1 and 2 were considered, whereas tier 3 variants were further included in the full analysis. Variants assigned to tier “Other” were not labeled oncogenic.

###### CIViC.

We used 01-Aug-2024-civic_accepted_and_submitted.vcf from CIViC^49^ (https://civicdb.org/releases/main) to annotate mutations. We retained variants annotated as “somatic” under CIViC Entity Variant Origin, of entity type “evidence” under CIViC Entity Type, and with a valid CIViC evidence rating (1–5). For the top-tier analysis, only variants with evidence levels A (validated), B (clinical), and C (case study) were included. Variants with evidence levels D (preclinical) and E (inferential) were additionally included in the full analysis.

###### ClinVar.

We used the ClinVar^50^ release clinvar_20260226.vcf (https://ftp.ncbi.nlm.nih.gov/pub/clinvar/vcf_GRCh37/). We retained only variants annotated as somatic (origin 2) that mapped to a valid gene and amino acid substitution. We further required their associated diagnosis explicitly referring to cancer, including disease-specific terms (myeloma, leukemia, lymphoma, sarcoma, glioma, glioblastoma, medulloblastoma, hepatoblastoma, mesothelioma, neuroblastoma, melanoma, retinoblastoma, astrocytoma, meningioma) or containing generic terms related to cancer (cancer, tumor, carcinoma, neoplasm). We excluded diagnoses labeled as “predisposing”, “hereditary”, or “familial” to aim at somatic cancer diagnoses, not hereditary predisposition. For top-tier analyses, only variants annotated as “Pathogenic” were considered oncogenic. Variants annotated as “Likely_pathogenic” or “Pathogenic/Likely_pathogenic” were included in the full analysis. Variants annotated as “Benign”, “Likely_benign”, or “Benign/Likely_benign” were not labeled oncogenic.

###### CKB.

We downloaded annotations on March 10, 2026 (https://ckb.genomenon.com/gene/grid) for the publicly available CKB CORE resource.^59^ For the top-tier analysis, only variants labeled “gain of function” or “loss of function” were annotated as oncogenic. Variants annotated as “gain of function – predicted” or “loss of function – predicted” were further included in the full analysis. Variants labeled as “no effect”, “no effect – predicted”, or “unknown” were not annotated as oncogenic.

###### OncoKB.

We downloaded annotations of somatic mutations from OncoKB on March 10, 2026 for all genes represented in our framework (e.g., https://www.oncokb.org/gene/TP53/somatic).^58^ For top-tier analyses, only variants labeled “Oncogenic” were considered, whereas variants labeled “Likely Oncogenic” were additionally included in the full analysis. Variants labeled “Likely Neutral” were classified as non-oncogenic.

To compare these oncogenic annotations from external databases with our framework, we classified mutations using the emission rate $log\;f_{{\overset{ˆ}{\omega }}_{i}}\left(e_{i}\right)$, where ${\hat{\omega }}_{i}$ denotes the most probable state of position i and $e_{i}$ the corresponding effect score. For each gene, we computed the mean $\mu_{0}$ and standard deviation $\sigma_{0}$ across all mutations in baseline state ${\hat{\omega }}_{i}=0$. We classified mutations as significant if ${\hat{\omega }}_{i}\neq 0$ and $log\;f_{{\overset{ˆ}{\omega }}_{i}}\left(e_{i}\right)\geq \mu_{0}+2\cdot \sigma_{0}$ (i.e., those in significant regions with a non-baseline emission score). For example, synonymous mutations were not classified as significant, even when located in a significant region. Based on these classifications, we compared the external support for significant vs. non-significant mutations, including the number of independent databases that annotated them as oncogenic (Figures 6H and S26B) and their effects in mutagenesis screens (Figure S26D), based on the following analyses:

####### 1. Comparison of overlap:

For each mutation, we determined the number of databases that annotated it as oncogenic (Figure 6G) and the exact combination of supporting databases (Figure S26A), either using all oncogenic annotations or using only top-tier annotations. For each combination, we further quantified the fraction of mutations that were also classified as significant by our framework (Figures 6H and S26B).

####### 2. Local coherence:

To assess the local coherence of external annotations, we stratified mutations according to whether they were annotated in ≥1 database, in ≥2 databases, or as top-tier in ≥1 database. We then examined whether mutations in the surrounding neighborhood of these mutations were also annotated in ≥1 external database. As a negative control, we repeated the same analysis for mutations not annotated in any database. Enrichment was expressed relative to the baseline fraction of all mutations annotated in ≥1database (Figure S26C).

####### 3. Mutagenesis screening data:

We evaluated functional mutagenesis data for *TP53*, *VHL*, and *PTEN*, which provide an experimental readout of the effect of each mutation (Figure S26D). Mutations were stratified according to whether they were significant vs. non-significant in our framework, as well as the number of external databases in which they were annotated as oncogenic, either based on all or only top-tier annotations. To facilitate comparison across genes, functional scores were min-max normalized within each gene. For *TP53*, we used data from Giacomelli et al. (2018)^60^ (column A549_p53WT_Nutlin-3_*Z* score); for VHL, we used Buckley et al. (2024)^61^ (function_score_final); and for PTEN, we used Mighell et al. (2018)^62^ (cum_score) (Figure S26D).

####### 4. Accumulation of annotations over time:

Since expert-curated databases are evolving continuously, we examined the growth of external databases over time. For this, we downloaded all archived ClinVar releases between May 16, 2017 and March 02, 2026 (https://ftp.ncbi.nlm.nih.gov/pub/clinvar/vcf_GRCh37/archive_2.0/). We processed their VCF files chronologically and accumulated observed variants (i.e., once a variant appeared in a release, it was retained at all subsequent time points). For each release, we quantified the difference in the cumulative number of oncogenic mutations relative to the initial version, separately for significant and non-significant mutations in our framework (Figure S26E). To account for the smaller size of significant regions, we normalized raw counts by the fraction of significant and non-significant mutations in our framework, respectively, thereby adjusting for the lower probability of annotating a significant mutation as oncogenic by random chance.

####### 5. Database-specific enrichment:

For each database, we quantified how often mutations were annotated as oncogenic, separately for significant vs. non-significant mutations in our framework (Figure S26F). As a baseline, we used the overall fraction of mutations annotated as oncogenic in each database (x-fold enrichment relative to baseline). We repeated these analyses after excluding variants supported by any of the other four databases, thereby ensuring that the enrichment was not driven by a few canonical oncogenic mutations but rather by less-annotated mutations that have yet to achieve broader consensus (e.g., the OncoKB analysis was restricted to mutations not annotated as oncogenic in ClinVar, CKB, COSMIC, or CIViC).

#### Design and validation of a proteo-genomic framework for identifying regions under tumorigenic selection

To characterize biologically meaningful mutation patterns in cancer genes, we developed a proteo-genomic framework that distinguishes regions under tumorigenic selection vs. regions shaped by background mutational processes. Our model accounts for several biological covariates that influence background mutation rates at the resolution of single genomic positions. For example, mutational processes often preferentially target specific sequence motifs, causing mutations that accumulate at genomic positions without contributing to tumorigenesis (e.g., UV- or APOBEC-driven mutagenesis).^90^ Conversely, not all positions within biologically relevant regions are expected to be highly mutated, because structural constraints in the amino acid code can limit the functional impact of some substitutions (e.g., if the mutated and non-mutated amino acids share similar biochemical properties, a mutation may have only limited consequences regardless of its position).^92^ By incorporating these and other variables into the model, our framework identifies biologically relevant regions in cancer genes enriched for oncogenic mutations. We used several analyses to assess the statistical and biological validity of our model.

##### Model design and covariates underlying the background mutation model

A central component of our framework is a Hidden Markov model that distinguishes discrete hidden states related to non-significant regions (the baseline state) vs. significantly mutated regions (all other states). The baseline state captures the expected distribution of mutations under neutral processes, whereas the remaining states represent deviations from this background after accounting for relevant covariates. Expected mutation rates are modeled as a function of multiple features, including sequence context (Figures S1A–S1C), allowing the model to separate true signals of tumorigenic selection vs. patterns driven by regional fluctuations in underlying mutational processes. We allowed significant regions to be represented by multiple states to account for cancer genes with varying regional selection signals (e.g., those with oncogenic mutations acting through several independent domains).

To avoid overfitting, we fixed a baseline state and penalized excessive transitions between states. To maximize robustness, we used diverse penalization and initialization strategies and confirmed that the results were consistent across 500 independently trained models per gene.

Our model is specifically designed for panel sequencing data used in AACR GENIE,^12,13^ where each patient typically has mutations annotated in only a limited number of genes, and the number of sequenced genes varies depending on the assay used. This creates challenges for tumor-type- or sample-specific background models used for whole-exome sequencing data. First, per-sample mutation counts are too sparse to reliably infer an individual background mutation spectrum, including contributions from distinct mutational processes. Second, differences in the set of sequenced genes across assays can lead to unstable parameter estimates when background models are fit separately for different panels. Third, because panel assays are enriched for cancer-relevant genes, the observed mutations do not provide an unbiased estimate of the background distribution, as these genes harbor substantial signal from positive selection. These effects are less prominent in whole-exome sequencing data because the large number of mutations in non-cancer genes dominates the overall distribution.^92^ Therefore, to avoid instability due to the smaller mutation counts and the higher fraction of genes under selection in panel sequencing data, we used a unified background model that aggregates mutations across all genes for this particular dataset.

##### Calibration of the background model across genomic positions

To systematically assess the calibration of our framework, we evaluated how well the fitted background model captured the fine-scale distribution of mutation counts across genomic positions in the sequenced genes. Because our study is based on a population-scale cohort of 139,818 tumor patients, accurate modeling of ultra-rare events is essential for distinguishing significant regions vs. long-tailed background distributions in non-significant regions. We tested the calibration of each component of our model:

###### 1. Background mutability in significant and non-significant regions.

We first examined the relationship between the observed number of mutations per genomic position vs. the predicted background mutability in our model based on its surrounding genomic sequence context. Using a sliding-window approach, we found a close, linear relationship between the context-dependent background mutation rate in our model and the average number of observed mutations per position (Figure S1A). This relationship was observed not only in significant and non-significant regions of cancer genes but also in non-significantly mutated genes, indicating that genomic sequence context is a major determinant of the baseline mutability of genomic positions. Importantly, the calibration remained robust after stratifying positions by pathogenicity (AlphaMissense), indicating that sequence context-dependent mutability is consistently captured across positions with different potential functional consequences (Figures S1B and S1C).

###### 2. Pathogenicity modulates mutation patterns in significant regions.

Besides sequence context, the pathogenicity of residues was another major factor in our model affecting the mutability of each position. Within significant regions, mutations accumulated at positions with high predicted pathogenicity (AlphaMissense)^15^; whereas in non-significant regions, mutations were distributed largely independently of predicted impact (Figure S2). To test whether sequence context and pathogenicity scores represented unrelated biological variables, we grouped mutations by their predicted pathogenicity and examined the distribution of their context-dependent background mutation rates, and vice versa (Figures S1B and S1C). In both directions, distributions were broadly similar across percentile bins, indicating that sequence context and pathogenicity are largely unrelated factors affecting the mutability of genomic positions (Figures S1B and S1C).

###### 3. Modeling of overdispersed mutation counts.

We assessed whether the statistical distribution used by our model was consistent with the mutation-count data observed at the population scale by comparing expected vs. observed mutation frequencies across genomic positions in genes. Our framework uses a Poisson-lognormal distribution to model variation in mutation counts across genomic positions. We compared this distribution against Poisson and Gamma-Poisson models, which are widely used in cancer genomics^92^ (Figures S3A–S3C). The Poisson-lognormal model showed close alignment between expected vs. observed mutation counts across the full range of the distribution. Indeed, Q-Q plots showed close agreement between expected and observed frequencies down to below 0.001% (Figure S3B). Importantly, this calibration remained robust across regions spanning different AlphaMissense scores (Figures S3A–S3C). By contrast, Poisson and Gamma-Poisson models deviated in the long tail of the distribution (Figure S3B). This difference was especially pronounced in positions with substantial pathogenicity scores, where differences between significant vs. non-significant regions were most pronounced (Figure S3C). Histograms of mutation counts further showed that the Poisson-lognormal distribution accurately recapitulated the empirical distributions in both significant and non-significant regions of cancer genes, as well as in non-significantly mutated genes, whereas Poisson and Gamma-Poisson models consistently underestimated the frequency of higher counts (Figure S3C). These results remained robust across positions grouped by pathogenicity score percentiles (Figure S3C). Moreover, differences of significant vs. non-significant regions were most prominent in their tails rather than in bulk distributions (Figure S2B). Notably, the same differences emerged when using the original pathogenicity scores and percentile-transformed values, indicating that they were not due to differences in the distribution of pathogenicity between regions (Figures S2C and S2D). Furthermore, similar patterns were observed when analyzing the fraction of mutated positions, rather than the total number of mutations, demonstrating that the association with structural effect was not driven solely by a small number of extreme hotspots (Figures S2E and S2F). These analyses highlight the need for Poisson-lognormal models to capture overdispersion in the long-tailed distribution at individual genomic positions and in order to distinguish regions under tumorigenic selection.

###### 4. Validation of the baseline state as a reference for neutral mutation patterns.

We next asked whether the baseline state of the Hidden Markov model accurately captured background mutation patterns in regions not under detectable positive selection. Q-Q plots comparing quantiles of observed mutation counts vs. those expected under the baseline distribution showed close agreement for non-significant regions in cancer genes as well as for non-significantly mutated genes (Figure S3D). By contrast, significantly mutated regions deviated from the baseline distribution, confirming that biological factors beyond the neutral background processes shape their mutation patterns (Figure S3D). These results support the baseline state as a reference for neutral mutation patterns, while deviations reflect biologically meaningful signals related to selection in significant regions.

Together, these analyses demonstrate that our framework is well calibrated across multiple components: the sequence-dependent mutability provides an accurate estimate of the background in significant and non-significant regions; pathogenicity scores further modulate mutation rates in significant regions; the baseline state offers a neutral reference in non-significant regions; and the Poisson-lognormal distribution models overdispersed mutation counts at the population scale. This calibration remains accurate even at very low mutation frequencies.

##### Validation of the model using mutagenesis screening data in *TP53*

We examined mutation patterns in *TP53* – a gene that contains numerous mutational hotspots in hypermutated contexts owing to its exceptionally high overall mutation frequency^60^ (Figure S4A). We thus used *TP53* to assess the ability of our model to distinguish genuine regional signals of tumorigenic selection vs. the accumulation of mutations in hypermutated contexts.

Indeed, when we analyzed raw mutation counts at genomic positions without correcting for covariates, *TP53* exhibited several hotspots that coincided with highly mutable sequence contexts (Figure S4A). However, after applying the background correction from our framework, most of these apparent hotspots disappeared (Figure S4A), suggesting that many position-specific peaks in the raw count data were largely explained by local background mutability rather than positive selection acting on individual genomic positions. Indeed, normalized counts were more consistent with biological constraints related to the location of functional domains (e.g., the DNA-binding domain) and structural features (e.g., the protein core) than raw mutation counts (Figure S4A).

To further validate the background calibration of our model in *TP53*, we used mutagenesis screening data in *TP53*, which provide a direct functional readout for each mutation. We assessed the key variables in our model and their relationships: the raw number of mutations per position, the tumorigenic effect of each mutation (impact on tumor cell growth in the mutagenesis screen), the predicted pathogenicity of mutations (AlphaMissense), and the location of mutations in significantly mutated vs. non-significant regions (Figures S4B and S4C).

Background mutation rates predicted by our model correlated with the raw number of mutations per position, both within significant and non-significant regions (Figure S4B). Critically, however, they showed little or no correlation with the experimentally measured effects of mutations on tumor cell growth (Figure S4B), suggesting that raw mutation counts did not directly reflect tumorigenic selection but were modulated by the background mutability of each genomic position. By predicting local background mutability and adjusting mutation counts accordingly, our model identifies regions in which mutation recurrence is unlikely to be explained by background mutational processes alone, thereby distinguishing significantly mutated from non-significant regions in cancer genes.

In line with this, pathogenicity scores correlated strongly with the effects of mutations on tumor cell growth in significantly mutated regions (Figure S4C), indicating that mutations in these regions had functional consequences relevant to tumorigenesis. In contrast, this relationship was weak or absent in non-significant regions (Figure S4C), and even mutations predicted to be pathogenic did not necessarily confer measurable effects on tumor cell growth.

Thus, these analyses provide experimental support for our model in *TP53* and its ability to distinguish background mutability from tumorigenic selection. Background mutation rates in our model capture variation that is largely independent of functional effects, whereas significantly mutated regions are enriched for mutations for which predicted pathogenicity aligns with experimentally measured effects on tumor cell growth.

Together, these results indicate that the background model in our framework provides a stable and well-calibrated reference for panel sequencing data. In particular, our framework captured the broader regional pattern without overfitting to pseudo-hotspots in *TP53*, suggesting proper calibration of context-dependent background mutation rates in their tail probabilities, which are particularly important for detecting significantly mutated regions.

##### Enrichment of functional residue annotations within significantly mutated regions

To further characterize the functional relevance of significantly mutated regions, we collected residue-level functional annotations from InterPro,^75^ given its widespread use and coverage of a large number of genes. Residues located within significantly mutated regions showed a significantly higher fraction of functional annotation than residues in non-significant regions (median fraction, 82.7% vs. 69.0%; *p* = 2.35 × 10^−4^, two-sided Wilcoxon rank-sum test), supporting the functional relevance of the signal captured by our model (Figure 1C).

This enrichment was observed for missense mutations in both oncogenes and tumor suppressors (Figures S5D and S5E). A similar but weaker trend was observed for truncating mutations in tumor suppressors, likely because truncating mutations impose fewer residue-level constraints on gene inactivation and occur across broader regions than missense mutations. Together, these results provide orthogonal support for our model using an external annotation resource.

#### Biological determinants of the size and spatial distribution of significantly mutated regions

We applied our model to tumor sequencing data from 139,818 patients across more than 25 tumor types. Across this cohort, we detected significantly mutated regions in 173 genes, most of which are established cancer genes in the COSMIC Cancer Gene Census^48^ (86.7%; 150/173) (Figures 1A and 1B; Tables S1, S2, and S3).

On average, significantly mutated regions spanned ~22.3% of the gene length, although sizes varied substantially across genes (interquartile range, 3.3%−35.4%) and across functional classes of genes (*p* < 0.001, Kruskal-Wallis test). Narrow significant regions were often associated with mutational hotspots or clusters targeting small-molecule binding sites, such as GTP in *KRAS* or D2HG in *IDH1* (Figures S5A–S5C). Broader regions were more common in genes for which mutations altered interactions with larger molecules, such as DNA binding in *FOXA1*, substrate recognition in *SPOP*, or catalytic activity in *EP300* (Figures S5A–S5C).

The functional effects of individual residues were a central determinant of mutation patterns (Figures S2A–S2H). Within significant regions, mutations accumulated at positions with high predicted pathogenicity (AlphaMissense),^15^ and this trend persisted after accounting for differences in sequence-context-dependent mutability. Outside significant regions, by contrast, mutations were distributed largely independently of predicted impact and occurred at similar rates at positions with high and low AlphaMissense pathogenicity scores (Figures S2A–S2H). This means that mutations occasionally hit a pathogenic position in non-significant regions, but these hits did not occur at rates higher than expected by chance, suggesting that mutations in these regions confer little or no selective advantage to tumor cells, analogous to the interpretation of dN/dS ratios in evolutionary biology.^92^ Moreover, in non-significant regions of cancer genes, mutation rates were similar to those observed in arbitrary non-cancer genes (Figures S2B–S2F), indicating distinct selective pressures in significant vs. non-significant regions, further supporting the biological rationale of our framework.

Mutation types were another key determinant of spatial distribution patterns (Figures 1D–1F; S8A–S8C). Truncating mutations (i.e., those introducing premature stop codons) generally affected broader regions than missense mutations, which alter amino acid identity in the protein sequence (*p* < 0.001, Mann-Whitney U test). For example, in *TP53*, missense mutations were largely confined to the DNA-binding and tetramerization domains, whereas truncating mutations were distributed across most of the gene (Figure 1D). However, several notable exceptions emerged. In *NOTCH1*, missense mutations targeted a broader and largely non-overlapping region relative to truncating mutations (Figure 1E). Likewise, truncating mutations showed clear regional preferences in *APC*, *MAX*, and *PPM1D*, whereas missense mutations did not (Figure 1F). These findings indicate that missense and truncating mutations can act through distinct mechanisms and associated functional constraints (Figures S8A–S8C), even when occurring in the same cancer gene.

##### Shared vs. tumor-type-specific mutation patterns in cancer genes

We asked whether cancer types use distinct mechanisms to (in)activate the same cancer gene. To address this question, we tested whether mutations preferentially targeted different regions within the same gene by comparing the mutation burden of each region across tumor types (Fisher’s exact test).

Overall, we found that most significantly mutated regions showed no significant enrichment of mutations across tumor types (72.5%, 348/480 regions) – either because genes were predominantly mutated in a single tumor type (e.g., *ESR1* in breast cancer, *POLE* in endometrial cancer, *RET* in thyroid cancer) (Figures 1B and S6E–S6G) or because mutations targeted the same regions across cancer types (Figure S7A). Once we accounted for differences in mutational spectra between tumor types (e.g., UV in melanoma vs. tobacco exposure in lung cancer), the same significant regions emerged across tumor types, suggesting that selection acted on shared functional constraints rather than on tumor-type-specific regions (Figure S7A).

However, we detected a few notable exceptions to this trend (Figures S7B–S7G). For example, lung adenocarcinomas exhibited *EGFR* mutations primarily in the kinase domain (suggesting catalytic activation and sensitivity to ATP-competitive inhibitors), whereas glioblastomas showed mutations in the extracellular domains of *EGFR* (consistent with activation via dimerization-mediated mechanisms) (Figure S7C). Other receptor tyrosine kinases showed the same patterns, including *ERBB2* and *ERBB3*, with mutations preferentially accumulating in extracellular vs. kinase-associated regions in different tumor types, suggesting tumor-type-specific modes of receptor activation (Figures S7D and S7E). These different mutation patterns may have implications for their response to TKIs vs. extracellularly targeted strategies.

For *CCND1*, blood tumors were enriched for mutations in the N-terminal domain (linked to CDK4/6 binding and cyclin-CDK complex formation), while endometrial carcinomas mainly carried mutations in the C-terminal loop (linked to protein turnover via phosphorylation-dependent degradation) (Figure S7F). These patterns suggest distinct, tumor-type-specific activation modes, either altering cyclin-CDK interactions or stabilizing cyclin D1 to prolong signaling through the CDK4/6 axis – with potential implications for CDK4/6 inhibitor sensitivity.

Lastly, mutations in *NOTCH1* showed a striking split between tumor types, consistent with its tumor-type-dependent role as an oncogene vs. tumor suppressor. Hematologic malignancies preferentially accumulated mutations in regions associated with NOTCH1 activation and receptor autoinhibition, whereas brain tumors showed enrichment of mutations in EGF-like repeats, consistent with altered ligand binding and receptor heterodimerization (Figure S7G). Therefore, mutations can have gain- or loss-of-function effects across tissues, depending on their location within *NOTCH1*, which has mechanistic implications for their interpretation.

Taken together, most cancer genes harbor mutations in the same regions across tumor types, once background mutation rates are controlled for, suggesting that selection acts on shared functional constraints. However, a subset of genes exhibits genuine tumor-type-specific differences across regions, consistent with distinct mechanisms of (in)activation, particularly receptors and genes with cell-type-dependent functions.

##### Association of truncating mutation patterns with nonsense-mediated decay

Nonsense-mediated decay (NMD) degrades transcripts containing premature termination codons, limiting the production of truncated proteins.^18^ In tumor suppressors, truncating mutations that trigger NMD are expected to cause loss of protein expression, conferring a selective advantage to tumor cells. However, truncating mutations near the C-terminus often escape this mechanism, allowing the transcript to remain stable and potentially yield a functional protein product. We thus hypothesized that the depletion of truncating mutations in C-terminal regions of cancer genes reflects constraints imposed by regions escaping from NMD, as they confer limited advantage to tumor cells.

To test this hypothesis, we predicted NMD-escape regions using established rules.^93^ We then quantified the depletion of truncating mutations within these NMD-escape regions using permutation tests that preserve both the length of individual significantly mutated regions and the residue-level mutational burden. We performed this analysis for 134 genes in which the average normalized mutation count in significant regions exceeded that in non-significant regions. Results are summarized across 19 oncogenes and 88 tumor suppressors as a volcano plot (Figure S9A).

Among tumor suppressors, *EP300* and *CREBBP* showed strong associations with predicted NMD-escape regions (Figure S9A), suggesting that the depletion of truncating mutations at their C-terminal ends was linked to NMD escape. To further examine the biological consequences of this pattern, we compared gene expression across patients in three groups (Figure S9C): (i) those with truncating mutations in the C-terminal NMD-escape region, (ii) those with truncating mutations in the rest of the gene, and (iii) those without truncating mutations in the gene. As expected, patients carrying truncating mutations outside the NMD escape region showed significantly reduced mRNA levels compared with non-mutated patients (*p* = 2.41 × 10^−5^ for *EP300; p* = 1.15 × 10^−5^ for *CREBBP*), consistent with NMD-mediated degradation of mutated transcripts. By contrast, patients with truncating mutations in the C-terminal escape regions did not show significant reductions in expression relative to non-mutated patients (Figure S9C). These results suggest that truncating mutations in NMD-escape regions have less pronounced effects on transcript levels and may provide a smaller selective advantage to tumors, explaining their depletion from these regions.

The size and impact of predicted NMD-escape regions varied substantially across tumor suppressors. For example, in *APC*, the final exon spans nearly half of the coding sequence and is predicted to be broadly NMD-insensitive (Figure S10A). Concordantly, truncating mutations were depleted across the entire second half of *APC*. In CDKN2A, by contrast, the terminal exon is short. In line with this pattern, the regional depletion of truncating mutations was less extensive in this gene (Figure S10A). In addition, single-exon genes such as *CEBPA* are not expected to engage NMD in the same way,^18^ indicating that these genes require a different interpretive framework.

Together, these results suggest that the transcript architecture is an important determinant of truncating mutation patterns in tumor suppressors. Regions associated with NMD escape help explain the regional depletion of truncating mutations in the C-terminus of many tumor suppressor genes.

As an orthogonal mechanism, we investigated whether alternative translation initiation (ATI) further contributed to the spatial distribution of truncating mutations in cancer genes. ATI initiates translation at downstream methionine codons and produces stable protein products, thereby bypassing N-terminal truncating mutations. This mechanism has been implicated in oncogenic activity in a few cancer genes.^96^

To quantify the association between truncating mutations and ATI, we identified downstream N-terminal methionines that could serve as alternative start codons using NetStart 2.0^87^ and TIS Transformer.^88^ We used the union of predictions from both tools when they were concordant; we selected the more downstream start codon when predictions were discordant. Analogous to our analysis of nonsense-mediated decay, we quantified the depletion of truncating mutations in the region between the canonical and alternative translation initiation sites for 25 genes that met two criteria: (1) the average normalized mutation count in significant regions exceeded that in non-significant regions, and (2) the predicted alternative start codon was located within the first half of the coding sequence, with more than 100 residues remaining downstream. Results are summarized in a volcano plot (Figure S9B).

Among tumor suppressors, *CYLD* was the only gene significantly associated with ATI, whereas others showed no significant depletion of truncating mutations in ATI-associated regions (Figure S10B). Therefore, mechanisms other than ATI are more likely to underlie the spatial distribution of truncating mutations in most tumor suppressors.

##### Structural constraints and 3D geometries shape missense mutation patterns in cancer genes

In most cancer genes, missense mutations showed complex spatial patterns, shaped by an interplay between constraints in 3D protein structures and the location of functional domains. We therefore sought to systematically define, for each cancer gene, which structural constraints shape the spatial distribution of its missense mutations. To this end, we quantified the overlap between significant regions and a wide range of structural features for each gene.

###### 1. Enrichment of missense mutations in secondary structures.

For each gene, we quantified whether significantly mutated regions were enriched in α-helices, β-sheets, or loops relative to a null distribution model that preserves overall mutational burden and residue coverage (permutation test). This analysis revealed that missense mutations accumulated in different secondary structures, particularly in tumor suppressors – indicating distinct modes of gene inactivation (Figures 4A and S13A–S13C).

In the structural biology literature, enrichment of mutations in α-helices has often been linked to perturbation of protein-protein interactions, because a substantial fraction of interaction interfaces are mediated by exposed helical faces and coiled-coils.^97^ Enrichment in β-sheets is commonly interpreted as effects on domain core stability, since β-strands frequently form the scaffold of folded domains and substitutions in these elements can destabilize packing and folding, potentially increasing misfolding and degradation.^67^ In contrast, enrichment in loops is often interpreted as reflecting disruption of regulatory segments and flexibility, although loops can have heterogeneous roles and are not always direct proxies for core stability.

In line with these mechanisms, we observed that mutation patterns were more frequently enriched for α-helices in tumor suppressors than in oncogenes, consistent with destabilizing mechanisms being more prominent in functional inactivation. Particularly strong enrichment was observed in *ARID1A*, *STK11*, and *CDKN2A* – in line with the baseline function of these tumor suppressors depending on their interaction with ligands (Figures 4A and 4B). Enrichment in β-sheets was also more frequent and stronger in tumor suppressors than in oncogenes, particularly pronounced in *FBXW7*, *VHL*, and *SPOP* – indicating that missense mutations altered the stability of their key domains, rather than their interactions with a specific ligand (Figures 4A and 4B). Unlike α-helices and β-sheets, loops were not enriched in tumor suppressors compared to oncogenes – consistent with the role of loops being more multifaceted and less directly linked to folding stability (Figures S13C and S19A).

###### 2. Associations of missense mutations with other structural features.

To capture additional structural constraints shaping the distribution of missense mutations, we quantified the overlap between significant regions and solvent accessibility, intrinsically disordered regions, and stability-related metrics derived from protein structural models (Figures 4A and S12A–S12C). In particular, we aimed to define mutation patterns that were diffuse in linear genomic sequences but coherent in 3D protein structures.

Similar to the overlap with secondary structures, associations with structural constraints were more pronounced in tumor suppressors than in oncogenes, reflecting that missense mutations tended to target the interior protein core (enrichment in residues with low solvent accessibility), structurally important regions (enrichment in regions with low intrinsic disorder), and residues with destabilizing effects (enrichment in residue substitutions that decrease the structural stability) more often in tumor suppressors vs. oncogenes (Figures 4A and S12A–S12C). Notably, however, tumor suppressors were not uniformly enriched for each of these properties; each gene exhibited a unique combination of these patterns, suggesting that missense mutations engage distinct mechanisms to inactivate tumor suppressor genes.

###### 3. Classification of 3D geometries of mutation clusters in protein structures.

In addition to structural features, we analyzed whether missense mutations formed coherent spatial clusters in 3D structures. Our motivation was to characterize mechanisms of mutations converging at a specific interaction site, such as ligand-binding pockets, DNA-binding surfaces, or substrate-recognition interfaces. To characterize distinct patterns, we classified mutations by their 3D geometry, distinguishing compact “pocket-like” clusters (often consistent with small-molecule/ion binding) from broader surface clusters (often reflecting macromolecular interactions, such as DNA or other proteins). Importantly, this analysis is inherently structure-aware, capturing mutation patterns that are not contiguous in linear genomic sequences but converge in the folded protein.

Overall, 3D clustering geometry was more prominent in oncogenes than in tumor suppressors (Figure S18A–S18D and S19A), suggesting that oncogenic activation more frequently requires changes to specific interfaces or interaction sites, whereas tumor suppressor inactivation more often arises through more distributed destabilizing effects. We further observed that 3D clustering geometries differed substantially between oncogenes, consistent with the spatial footprint of the perturbed interaction partner (Figures S18A–S18D). For example, in *KRAS*, mutations converged on a focal site in 3D, consistent with perturbation of a compact functional interaction site with GTP. In contrast, mutations in *FOXA1* and *FBXW7* were more consistent with an extended 3D “linear” imprint, compatible with perturbation of interactions involving larger binding partners (e.g., DNA or protein substrates). In *PTEN*, mutations clustered more widely across a larger portion of its surface – consistent with their role in modulating protein complex formation.

###### 4. Structure-based distinction of oncogenes vs. tumor suppressors based on mutation patterns.

We next integrated structure-derived features into a unified model to distinguish oncogenes vs. tumor suppressors based on mutation patterns. To ensure this assessment was structure-driven, the model was blinded to oncogene vs. tumor-suppressor labels and provided only with quantitative features describing how missense mutation patterns align with each feature. We also intentionally excluded truncating mutations because they are strongly skewed toward tumor suppressors and would inflate classification performance without insights into structural features.

Using this joint model, oncogenes robustly separated from tumor suppressors based on the structural context of their significantly mutated regions (AUROC = 0.86). Inspection of model coefficients indicated that discrimination was driven primarily by stability-related features and enrichment at the protein core, consistent with missense mutations causing structural destabilization in tumor suppressors (Figures 4B and S19A).

We further evaluated the regression model on genes that were not annotated as oncogenes or tumor suppressors, but often had dual roles in both categories. Our model accurately distinguished these cases (Figure S19B), highlighting the biological value of structural information for interpreting patterns of significantly mutated regions in cancer genes – particularly in cases where mutation patterns are more complex than dichotomous labels of oncogenes vs. tumor suppressors and exhibit multifaceted effects.

###### 5. A joint structure-feature matrix reveals molecularly coherent subgroups of cancer genes.

To move beyond supervised classifications, we integrated structure-derived features into a joint matrix (signed log *p*-values, with the directionality of enrichment vs. depletion for each feature). We used these features to arrange cancer genes into molecularly coherent subgroups (see Figures 4C and 4D for an overview). Notably, most clusters harbored associations with multiple structural features. For example, localization of missense mutations in secondary structures often co-occurred with their enrichment at residues that impact protein structure, suggesting that most missense mutation patterns are shaped by the interplay of multiple functional and structural constraints (Figures 4C and 4D).

Within tumor suppressors, subclusters primarily reflected whether missense mutations were primarily enriched in (i) α-helices, (ii) β-sheets, (iii) loops, or (iv) exhibited weak alignment with any secondary-structure criteria – likely reflecting different modes of gene inactivation (Figure 4C). Within oncogenes, clusters aligned primarily with the 3D geometry of mutational clusters, distinguishing (i) compact single-site clusters (compatible with small-ligand or highly localized interface perturbation), (ii) extended linear patterns (consistent with larger interfaces, such as DNA or extended substrates), (iii) broader surface-like patterns (consistent with extended protein interfaces), and (iv) oncogenes without a clear geometry of their clusters (Figure 4D). Thus, significantly mutated regions with 3D structural features provided a principled framework for grouping cancer genes into biologically informative groups beyond the traditional oncogene vs. tumor suppressor dichotomy.

Furthermore, structure-based associations primarily affected the spatial distribution of missense mutations, whereas these associations were largely absent for truncating mutations, even when co-occurring within the same cancer genes (Figures S16A–S16F and S18E–S18H). This suggests that the distribution of missense mutations is often driven by the 3D protein structure, whereas truncating mutations are shaped by nonsense-mediated decay.

###### 6. Comparison of pLDDT and ΔΔG related to structural constraints and stability.

AlphaFold structures are annotated with the pLDDT metric^14^ (predicted local distance difference test), which reflects the local confidence of AlphaFold at modeled residue positions. It was frequently associated with missense mutations in our framework (Figures 4B and S12B), and we thus further investigated the underlying biology of this association.

Although low pLDDT scores are frequently observed in intrinsically disordered regions,^98^ they may also arise from other factors, including limited evolutionary signal, low multiple-sequence alignment (MSA) depth, or other sources of modeling uncertainty. Therefore, low pLDDT values should not be interpreted as definitive indicators of intrinsic disorder. To complement this metric, we computed predicted mutation-induced changes in protein stability (ΔΔG) using FoldX^53^ and compared these estimates with pLDDT scores.

Across residues, these metrics revealed a robust correlation, particularly in genes with truncating mutations depleted in their N- and C-terminal loops (e.g., *CCND1/2/3*, *CDKN2A*, *PTEN*, *PPM1D*, *STK11*, *TP53*, and *VHL*) (Figure S17A). Both pLDDT scores and ΔΔG scores were significantly lower in the mutated regions of *CCND1/2/3* and *PPM1D*, as well as in regions lacking truncating mutations in *CDKN2A*, *PTEN*, *STK11*, *TP53*, and *VHL* (Figures S17B and S17C). In other genes, ΔΔG and pLDDT scores were generally correlated, but not fully equivalent (Figures S17D and S17E). These results indicate that while pLDDT can serve as a proxy for stability in N- and C-terminal loops, ΔΔG provides a more direct measure of mutation-induced protein stability impacts.

#### Benchmarking significant regions against independent methods

To provide complementary views of oncogenic mutations and assess the robustness of benchmarking results across different curation strategies, we used three independently curated reference sets: the COSMIC Cancer Mutation Census,^48^ CIViC,^49^ and ClinVar^50^ (cancer-specific somatic mutations only). Because expert-curated knowledge bases are continuously evolving, absence from COSMIC, CIViC, or ClinVar does not imply that a mutation is non-oncogenic. Instead, these resources serve as a proxy for the “ground truth,” and benchmarking assesses whether a method prioritizes the same signals that expert curators deem relevant to tumorigenesis. Benchmarking against these resources further assesses a signal that is specific to cancer – whereas mutations with effects outside cancer (e.g., those altering the function of a protein in a way that is not advantageous to cancer) are not captured by these resources.

We compared our framework with driver identification methods^54–56^ (OncodriveCLUSTL, ActiveDriver, HotSpot3D) and pathogenicity scores^15,51–53^ (AlphaMissense, CADD, phyloP, FoldX). HotSpot3D and OncodriveCLUSTL are designed to detect hotspots or clusters of oncogenic mutations, whereas ActiveDriver focuses on oncogenic mutations enriched in functional contexts (e.g., sites related to post-translational modification or protein interaction). By contrast, pathogenicity scores prioritize mutations that are important for the protein function in general, regardless of a specific disease context. Thus, high pathogenicity scores of mutations do not necessarily imply selection for tumorigenesis.

##### Characterizing different target signals of methods

We used two representative genes, *KRAS* and *TP53*, to illustrate how differences in the design of methods affect their enrichment of oncogenic mutations. *KRAS* is dominated by a few oncogenic hotspots, and significant regions from our framework closely tracked these established hotspots (Figures 5B and 5C). By contrast, OncodriveCLUSTL missed less recurrent hotspots, and HotSpot3D and ActiveDriver extended into residues not clearly supported by positive selection. Besides canonical hotspots, our framework identified a significant region near L23, where the mutational pattern was subtler but still reached statistical significance (Figure 5B). This region has been implicated in KRAS-RAF1 interactions through the RAF1 cysteine-rich domain^42^ and also emerged as tumorigenic in cell-line mutagenesis screening data (Figure S10G), indicating an additional biological signal detected by our framework.

In *TP53*, oncogenic mutations were distributed across multiple segments of the DNA-binding and tetramerization domains, rather than being restricted to a few hotspots. Our framework tracked these annotated subregions closely (Figures 5B and 5C). By contrast, HotSpot3D broadened significance to residues not labeled as oncogenic in COSMIC, OncodriveCLUSTL did not recover all oncogenic segments, and ActiveDriver extended into less oncogenic regions (Figure 5B).

To generalize these findings across cancer genes, we performed benchmarking analyses for each gene (Figures 5A and S20A–S20C). Performance varied substantially across genes (distribution of AUROC). Some genes favored structure-aware clustering approaches (Hotspot3D), whereas others were better captured by functional-site-centric models (ActiveDriver), indicating that oncogenic mutations follow different patterns across cancer genes. Across different genes, our framework consistently ranked among the top-performing methods (Figures 5A and S20A–S20C). It was typically followed by HotSpot3D and AlphaMissense, although the relative ordering varied across genes, depending on whether performance was assessed at residue- or genomic-position resolution (Figures 5A and S20A–S20C).

These examples illustrate that differences between methods are not only quantitative but also reflect the distinct biological signals that each tool is designed to detect (e.g., general pathogenicity prediction, evolutionary constraints, clustering of somatic events, or tumor-specific selection).

##### Systematic ranking of methods reflects differences in their biological design

For systematic benchmarking, we ranked genomic positions or amino acid residues according to the pathogenicity score or significance value assigned by each method (Figures 5A and S20A–S20C). We compared these rankings against COSMIC, CIViC, and ClinVar using ROC curves. We performed the analyses both at the resolution of genomic positions and residues to maximize interpretability, while enabling a fair comparison of methods that do not provide scores at single genomic positions (ActiveDriver, FoldX) (Figures 5A and S20A–S20C).

Consistent with their different designs, recurrence-based driver identification methods generally exhibited greater enrichment for oncogenic mutations in COSMIC, CIViC, and ClinVar than general-purpose pathogenicity scores, which do not directly evaluate tumor-specific signals (Figures 5A and S20A–S20C). Furthermore, HotSpot3D (which incorporates 3D structural information) ranked higher than OncodriveCLUSTL and ActiveDriver (Figures 5A and S20A–S20C). Likewise, pathogenicity scores that use structural features (AlphaMissense, FoldX) ranked higher than phyloP and CADD, highlighting the added value of structural information for resolving tumorigenic signals. At the same time, FoldX-derived ΔΔG alone exhibited lower enrichment for oncogenic mutations than AlphaMissense, highlighting the added value of combining proteomic with complementary signals for detecting tumorigenic mutations (Figures 5A and S20A–S20C).

##### Shapes of ROC curves reveal differences in sensitivity to focal versus diffuse oncogenic signals

Beyond total AUROC, the shape of the ROC curves provided additional insight into the types of signals detected by each method (Figures 5A and S20A–S20C). For example, HotSpot3D showed a more front-loaded profile, with rapid early enrichment followed by a flatter trajectory, suggesting strong prioritization of sharply localized hotspots of oncogenic mutations but incomplete recovery of more broadly distributed oncogenic mutations (Figures 5A and S20A–S20C). By contrast, AlphaMissense showed weaker enrichment among the top-ranked residues but improved more gradually later in the ranking, consistent with lower selectivity for narrow clusters of oncogenic mutations but broader coverage of less recurrent oncogenic mutations (Figures 5A and S20A–S20C). Our framework combined strong early enrichment with sustained recovery across the full ranking, indicating that it detected both compact, high-confidence clusters of oncogenic mutations as well as broader patterns of less recurrent oncogenic mutations (Figures 5A and S20A–S20C). These patterns suggest that, by integrating genomic and structural signals, our framework achieves a favorable balance between prioritizing highly recurrent tumorigenic mutations within clusters early and recovering oncogenic mutations that are distributed more broadly.

#### Association of significant regions with clinical outcomes and treatment response

To link significant regions in cancer genes to clinically interpretable patient outcomes, we assessed whether mutations in significant vs. non-significant regions were linked with differences in treatment response and clinical outcomes. To this end, we analyzed two independent cohorts with matched genomic and clinical outcome data.

##### Clinical associations in the Hartwig Medical Foundation cohort

The Hartwig Medical Foundation (HMF) dataset^2,77^ contains therapy response (duration of therapy) and genomic data from 2,188 cancer patients. Therapies in this dataset range from targeted agents (e.g., EGFR inhibitors) to broader systemic regimens (e.g., chemotherapy, hormonal therapies) (Figure S24C). To ensure robust statistical power and minimize spurious associations, we restricted analyses to nine therapies with sufficient numbers of patients with both mutation and treatment-duration data (alkylating agents, anthracyclines, folate antagonists, platinum-based therapies, pyrimidine antagonists, taxanes, aromatase inhibitors, selective ER modulators, and EGFR inhibitors). We used Cox regression to compare treatment efficacy between mutated vs. non-mutated patients.

In aggregate, mutations in significant regions showed stronger associations with clinical outcomes than those in non-significant regions of the same genes (*p* = 0.059), supporting the clinical relevance of significant regions identified by our framework (Figure S24D). Importantly, this difference was not driven by a single treatment but was consistent across the nine therapies examined (median absolute log hazard ratios for mutations in significant vs. non-significant regions across genes), including alkylating agents (0.211), anthracyclines (0.094), folate antagonists (0.095), platinum-based therapies (0.063), pyrimidine antagonists (0.151), taxanes (0.055), aromatase inhibitors (0.081), selective estrogen receptor modulators (0.159) and EGFR inhibitors (0.732) – even though differences did not reach statistical significance for individual therapies, because their sample sizes were smaller than that of the aggregate cohort (Figure S24E).

##### Clinical associations in the GENIE Biopharma Collaborative cohort

The GENIE Biopharma Collaborative (BPC) cohort^12,13^ contains detailed treatment-response information for patients with breast cancer (*n* = 716), lung cancer (*n* = 1,125), and colorectal cancer (*n* = 965). This cohort also includes a broad set of clinical covariates, which we incorporated into our models to ensure that observed associations were specifically related to mutational patterns. For each cancer type, we compared progression-free survival after treatment initiation between mutated vs. non-mutated patients, while adjusting for subtype, tumor stage, metastatic burden, sub-histology, and other variables known to influence treatment response (Cox proportional hazards model).

We first focused on the outcomes of patients harboring significant mutations in cancer genes (i.e., mutations with high scores in our framework) and compared them with the outcomes of patients without mutations in the same gene. In several cancer genes, significant mutations were associated with known clinical response patterns (Figures 6F, S25A, and S25B): In breast cancer, significant *TP53* mutations were associated with worse outcomes following treatment with pegylated liposomal doxorubicin, an anthracycline chemotherapy used in breast cancer. In addition, significant *ESR1* mutations were associated with differences in progression-free survival with leuprolide, a gonadotropin-releasing hormone agonist that suppresses estrogen production.

In colorectal cancer, patients harboring significant *KRAS* or *FBXW7* mutations showed worse progression-free survival following treatment with fluorouracil and leucovorin (Figures 6F, S25A, and S25B). In non-small cell lung cancer, significant *KRAS* mutations were associated with poorer outcomes after carboplatin and paclitaxel treatment, indicating that *KRAS*-mutant lung cancers responded less well to taxane- and platinum-based chemotherapies (Figures 6F, S25A, and S25B).

We then performed the same analyses for patients harboring non-significant mutations in the same cancer genes (i.e., those with low significance scores in our framework) (Figures 6F, S25A, and S25B). In contrast to significant mutations, these patients generally did not show distinct response patterns and resembled patients without mutations in the corresponding genes. These differences indicate that significant mutations identified by our framework align with known response patterns and resistance phenotypes associated with these cancer genes, whereas the effects are less prominent for non-significant mutations. Treatment response depends on the specific location of mutations within cancer genes, and our framework can prioritize mutations that are particularly informative for predicting therapy response and clinical outcomes, with potential implications for stratifying patients into clinically meaningful subgroups.

## Supplementary Material

1

2

3

4

5

6

7

8

9

10

11

SUPPLEMENTAL INFORMATION

Supplemental information can be found online at https://doi.org/10.1016/j.ccell.2026.07.017.

## Figures and Tables

**Figure 1.**
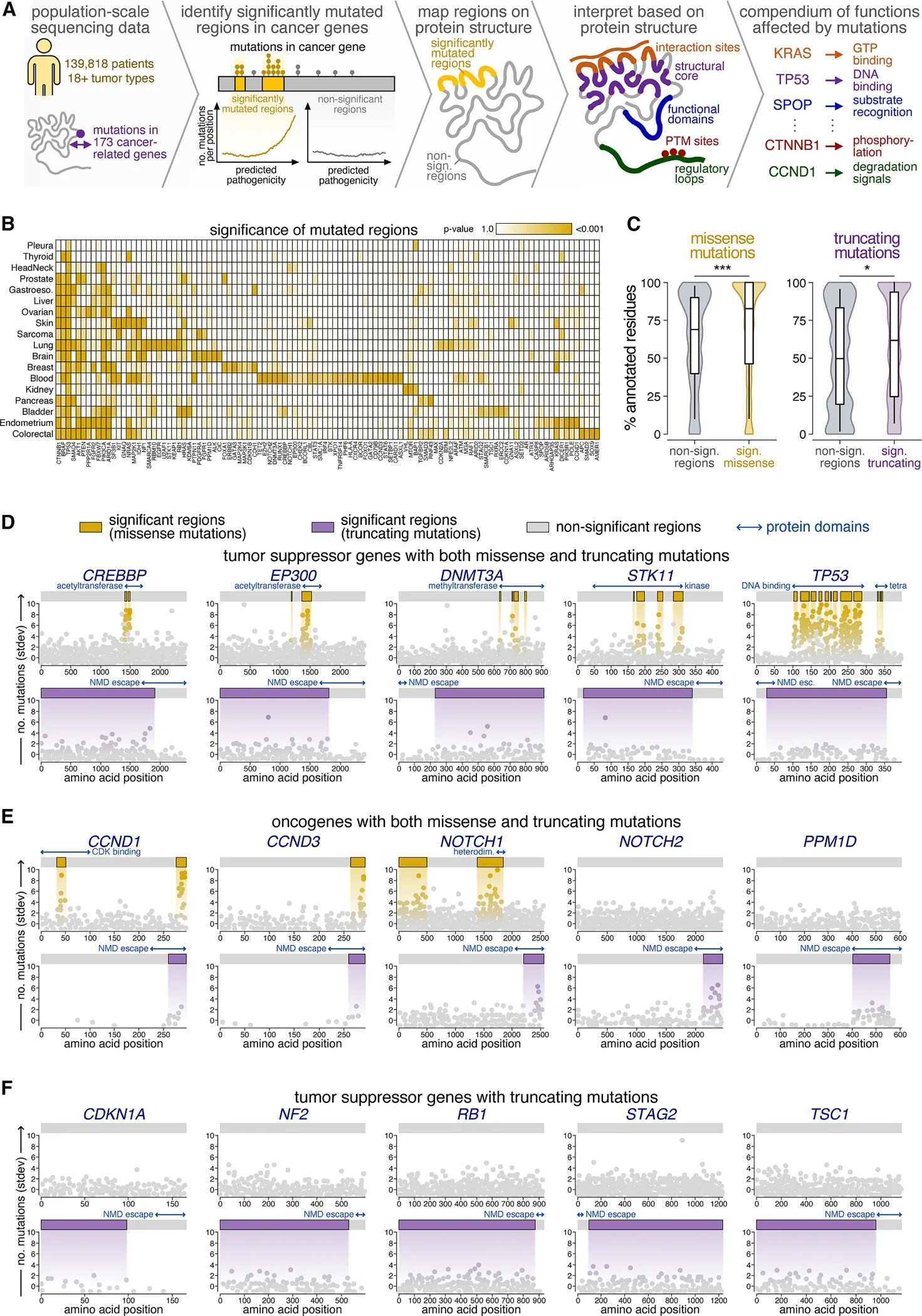
Proteo-genomics-guided dissection of somatic mutations (A) We integrated protein structures with sequencing data (139,818 patients) to identify significantly mutated regions in 173 cancer-related genes across 18+ tumor types and link them to tumorigenic functions. (B) Heatmaps visualize whether genes (columns) harbored a significant excess of mutations in significant regions across 18 tumor types spanning 90% of our cohort (rows). (C) Violin plots compare the fraction of functionally annotated residues (*y* axis) in significant (yellow: missense; purple: truncating mutations) vs. non-significant regions (gray) across genes with higher normalized mutation counts in significant vs. non-significant regions. The median (horizontal lines), interquartile range (boxes), 10^th^/90^th^ percentiles (whiskers), and significant differences (asterisks; *p < 0.05, ***p < 0.001; two-sided Wilcoxon rank-sum test) are annotated. (D–F) We plotted the position of significant regions (*x* axis; yellow: missense; purple: truncating mutations) and the number of mutations per position (*y* axis), normalized as standard deviations from the mean (*Z* scores). Double-headed arrows annotate functional domains and N- or C-terminal NMD-escape regions that show significant associations with mutation patterns. NMD, nonsense-mediated decay. See also Figures S1–S8 and Tables S1, S2, S3, and S4.

**Figure 2.**
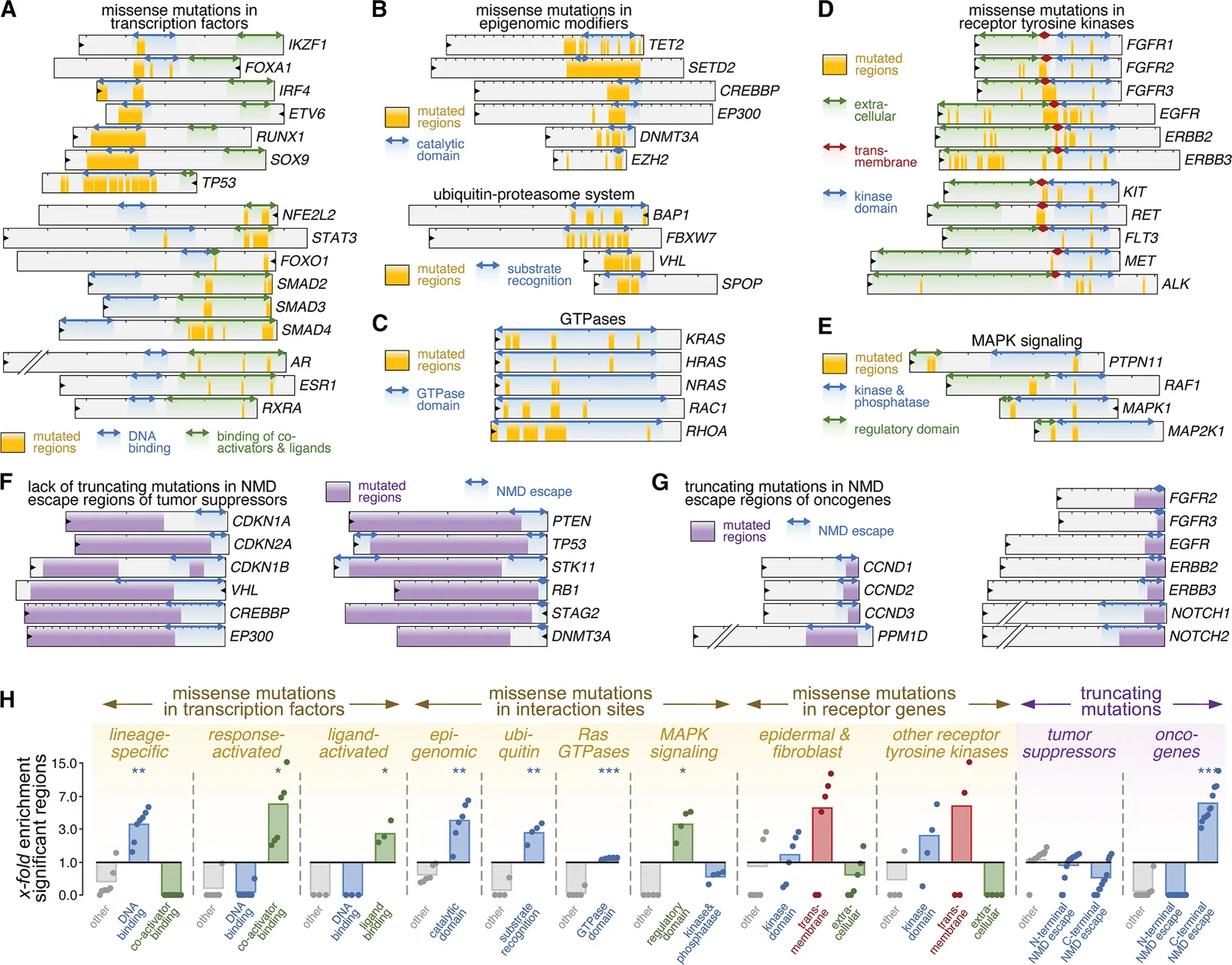
Overlap of significant regions and functional domains (A–G) We visualized the overlap between significant regions (yellow: missense; purple: truncating mutations), functional domains, and regions predicted to escape nonsense-mediated decay (NMD) (double-headed arrows). Functionally related genes were plotted with the same amino acid scale (100 amino acids per tick). Protein direction is indicated (arrows pointing from N to C termini). Genes were horizontally positioned to align their significantly mutated regions and examine whether they overlapped with similar domains. (H) We quantified the overlap between significant regions (yellow: missense; purple: truncating mutations) and annotated functional features for genes in (A)–(G). Enrichment >1.0 indicates greater overlap of the feature with significant vs. non-significant regions. We also quantified enrichment outside annotated features (“other,” gray). Bar graphs show the mean enrichment for each feature (*y* axis; log-transformed scale) and values for individual genes (dots). Asterisks indicate whether the enrichment differed significantly from 1.0 (one-sample, two-tailed Student’s *t* test; *p < 0.05, **p < 0.01, and ***p < 0.001). See also Figures S9 and S10.

**Figure 3.**
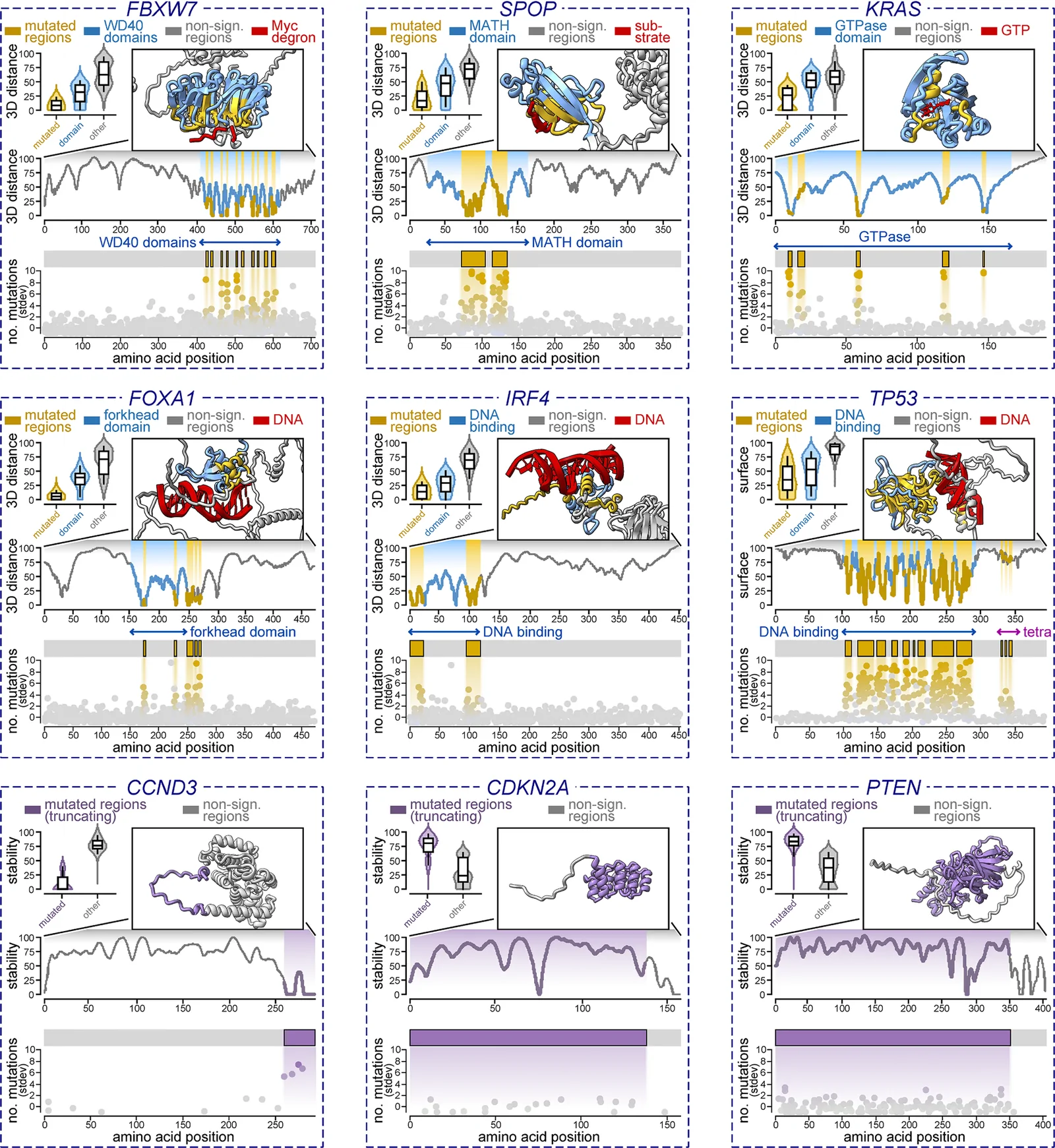
Significant regions align with structural constraints and functional domains Top left: violin plots show the distribution of structural features (% of max.) in significant regions (yellow, missense; purple, truncating mutations), non-significant parts of domains (blue), and the rest of each gene (gray), marking the median (horizontal lines), interquartile range (boxes), and 10^th^/90^th^ percentiles (vertical lines). Top right: structures of significant regions (yellow: missense; purple: truncating mutations), non-significant parts of domains (blue), the rest of each gene (gray), and annotated ligands (red). Middle/bottom row: we plotted structural features (*y* axis, % of max.) and the number of mutations (*y* axis, *Z* scores) against their position (*x* axis). See also Figure S11.

**Figure 4.**
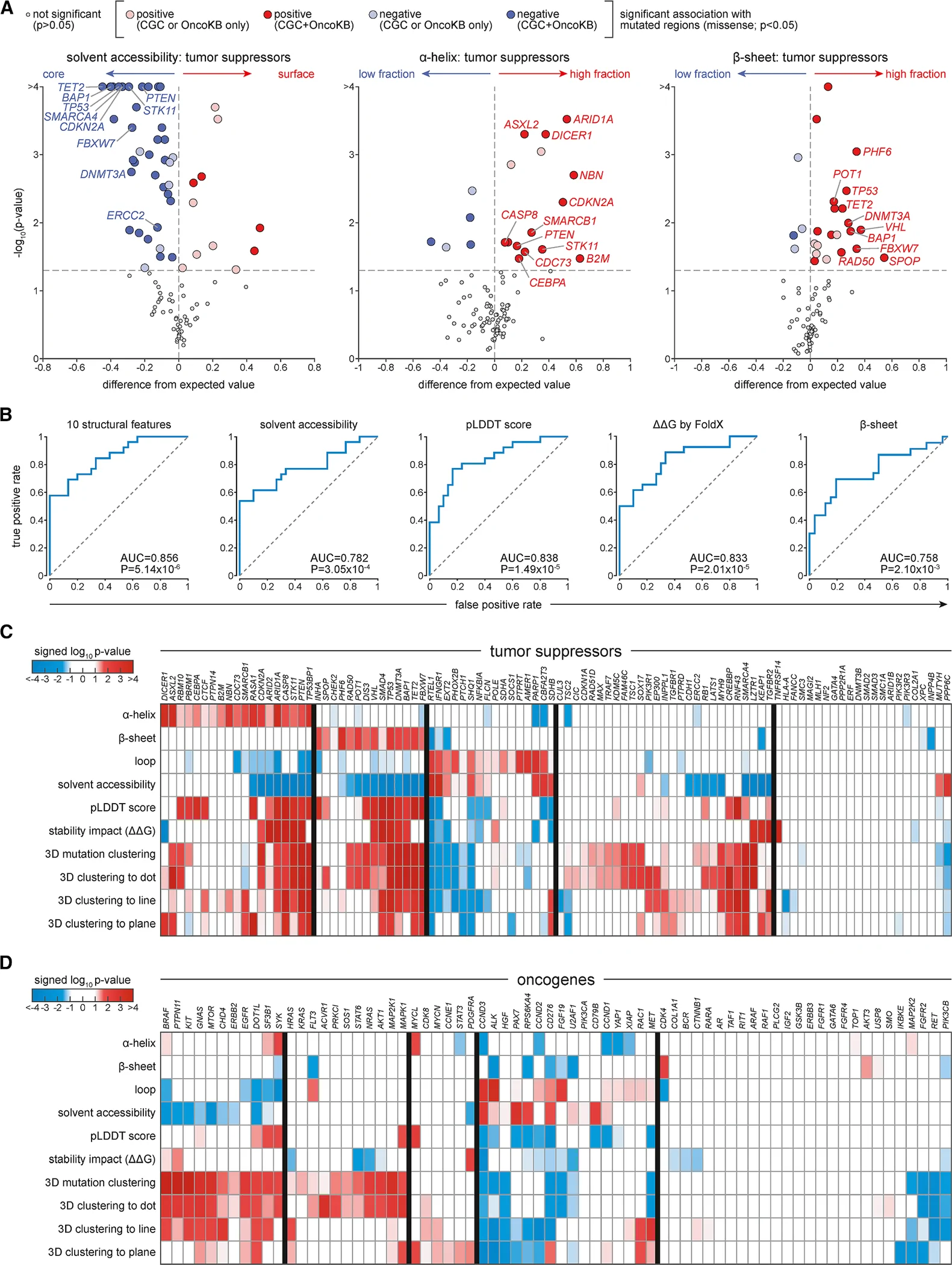
Significant regions distinguish modes of cancer gene (in)activation (A) We calculated the mean solvent accessibility of residues (left) and fraction of residues located in α-helices (middle) or β-sheets (right) within significant regions. We randomly shuffled regions of the same length and repeated these calculations. Volcano plots show the resulting one-sided permutation significance (*y* axis) and deviation from the permutation-based expectation (*x* axis) for each gene (dots). Genes in the Cancer Gene Census (CGC) or OncoKB are highlighted (darker shades). (B) Receiver operating characteristic (ROC) curves quantify the degree to which missense mutations in tumor suppressors were associated with the indicated structural features relative to oncogenes. Area under the curve (AUC) and *p* values (two-sided Wilcoxon rank-sum test) are annotated for each ROC curve. (C and D) Tumor suppressors (C) and oncogenes (D) were grouped by the association of their significant regions and structural features (rows). Heatmaps show signed −log_10_ *p* values from permutation testing for positive/negative associations (red/blue). See also Figure S12–S19 and Table S5.

**Figure 5.**
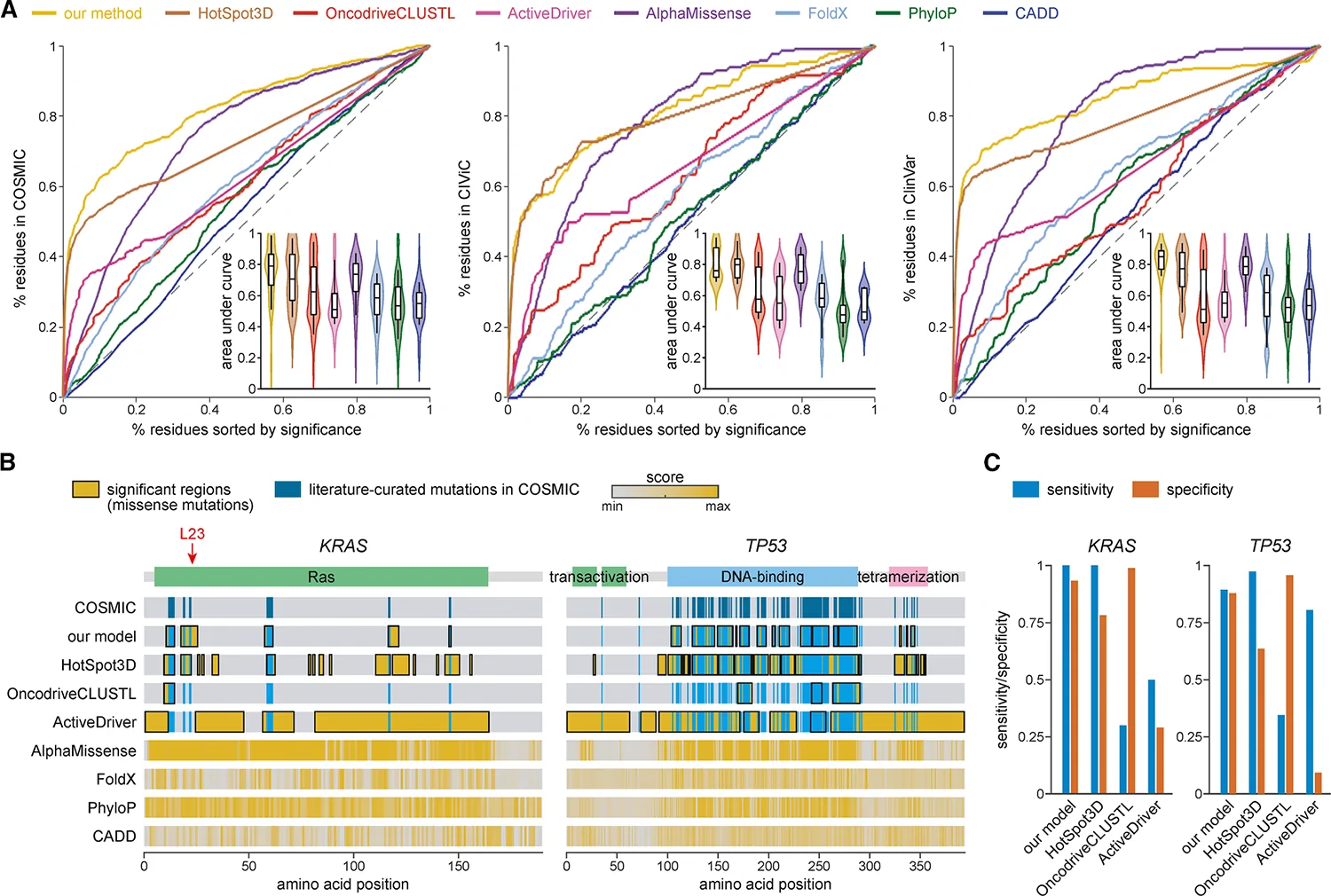
Significant regions are enriched for literature-curated oncogenic mutations (A) ROC curves quantify the enrichment of oncogenic mutations in significant regions with COSMIC (left), CIViC (middle), and ClinVar (right) as references (*y* axis). ROC curves are further displayed for driver identification methods (HotSpot3D, OncodriveCLUSTL, and ActiveDriver) and pathogenicity scores (AlphaMissense, FoldX, PhyloP, and CADD). Violin plots (insets) show the area under the ROC curve across genes, indicating their median (horizontal lines), interquartile range (boxes), and 10^th^/90^th^ percentiles (vertical lines). (B) We compared oncogenic mutations (COSMIC; blue) vs. significant regions from our model, results from driver identification methods (HotSpot3D, OncodriveCLUSTL, and ActiveDriver), and pathogenicity scores (AlphaMissense, FoldX, PhyloP, and CADD) (orange; one score/method per row). (C) Bar graphs display the sensitivity and specificity (*y* axis) of each method for oncogenic mutations in *KRAS* and *TP53*. See also Figure S20 and Table S6.

**Figure 6.**
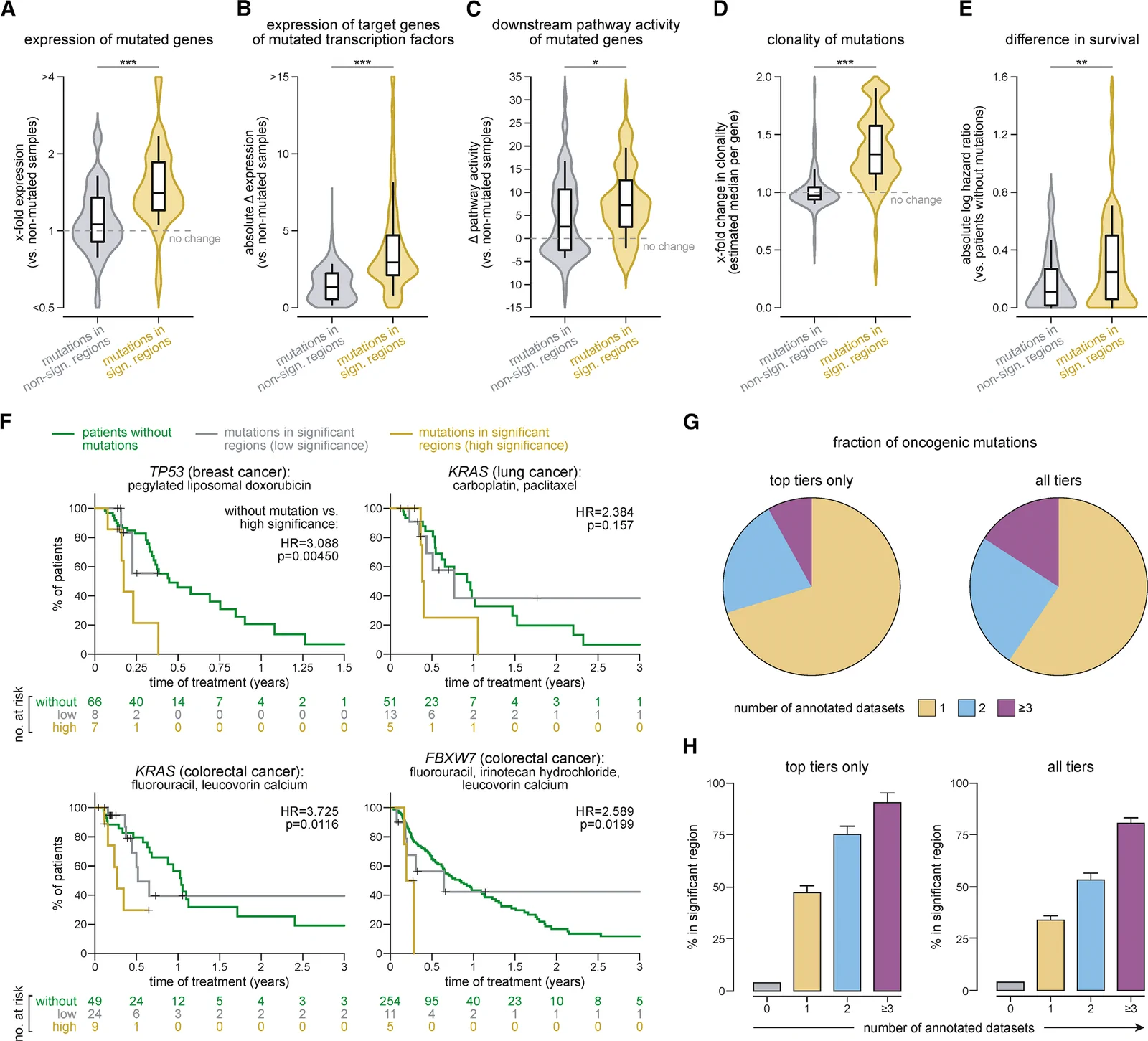
Significant regions localize mutations with biological or clinical relevance (A–E) We compared mutations in significant vs. non-significant regions based on (A) the expression of mutated genes relative to non-mutated samples, (B) differential activity of mutated vs. non-mutated transcription factors (target-gene expression), (C) downstream signaling of mutated vs. non-mutated samples (expression signatures), (D) relative clonality of mutations, and (E) survival of patients with vs. without mutations in regions/genes (hazard ratios; Cox regression). Violin plots show these metrics for mutations in significant (yellow) vs. non-significant (gray) regions, indicating medians (horizontal lines), interquartile ranges (boxes), 10^th^/90^th^ percentiles (whiskers), and baseline of non-mutated samples (dashed horizontal lines). Asterisks denote significant differences (*p < 0.05, **p < 0.01, and ***p < 0.001; two-sided Wilcoxon rank-sum test). (F) Kaplan-Meier curves show progression-free survival after initiation of the indicated therapies in patients without missense mutations in the indicated genes (green) and patients with missense mutations in significant regions, with mutation-level significance above (yellow; high significance) or below (gray; low significance) the median mutation-level significance in that gene in our framework. Ticks mark censored events (omitted for patients without mutations for clarity). Numbers of patients at risk are shown at the bottom. *p* values compare patients with high-significance mutations (yellow) vs. patients without mutations in the indicated genes (green) with two-sided log rank tests adjusted by the Benjamini-Hochberg procedure. Hazard ratios (HRs) were estimated with Cox proportional hazards models. Comparisons between patients with low-significance mutations (gray) vs. without mutations (green) were non-significant for all displayed genes and therapies. (G and H) We compared annotations of oncogenic mutations in ClinVar, CKB, COSMIC, OncoKB, and CIViC. (G) Pie charts summarize the fraction of oncogenic annotations that are supported by 1 (orange), 2 (blue), or ≥ 3 databases (purple). (H) Bar graphs show the fraction of mutations annotated in none (gray), 1 (orange), 2 (blue), or ≥ 3 databases (purple) that fall within significant regions—for top-tier (left) and all annotations (right). Whiskers indicate 95% confidence intervals (Wilson method). See also Figure S21–S26 and Tables S7, S8, S9, and S10.

**KEY RESOURCES TABLE:** 

REAGENT or RESOURCE	SOURCE	IDENTIFIER
Deposited data		
AACR Project GENIE (v15.0)	Pugh et al.^12,13^	https://doi.org/10.7303/syn53210170
gnomAD (v4)	Chen et al.^68,69^	https://gnomad.broadinstitute.org/downloads
ExAC (final release)	Lek et al.^70^	https://gnomad.broadinstitute.org/downloads
UCSC chain files	UCSC Genome Browser^71^	https://hgdownload.soe.ucsc.edu/goldenPath/hg38/liftOver/
AlphaMissense scores	Cheng et al.^15^	https://console.cloud.google.com/storage/browser/dm_alphamissense
AlphaFold2 protein structures	Jumper et al.^14,72^	https://ftp.ebi.ac.uk/pub/databases/alphafold/latest/UP000005640_9606_HUMAN_v4.tar
PDB protein structures (integration into website)	PDBe-KB consortium^73^	https://www.ebi.ac.uk/pdbe/pdbe-kb/
PDB protein structures (modeling of ligands)	Berman et al.^74^	https://www.rcsb.org
InterPro	Blum et al.^75^	https://www.ebi.ac.uk/interpro/download/
Mutagenesis screening data (*KRAS*)	Kwon et al.^39^	https://doi.org/10.1101/2024.10.22.618529
Mutagenesis screening data (*POT1*)	Martin et al.^37^	https://doi.org/10.1101/gad.352492.124
Mutagenesis screening data (*PPM1D*)	Kahn et al.^38^	https://doi.org/10.1182/blood-2018-05-850339
Mutagenesis screening data (*TP53*)	Giacomelli et al.^60^	https://doi.org/10.1038/s41588-018-0204-y
Mutagenesis screening data (*VHL*)	Buckley et al.^61^	https://doi.org/10.1038/s41588-024-01800-z
Mutagenesis screening data (*PTEN)*	Mighell et al.^62^	https://doi.org/10.1016/j.ajhg.2018.03.018
COSMIC Mutation Census (v100)	Sondka et al.^48^	https://cancer.sanger.ac.uk/cmc/home
ClinVar database (GRCh37)	Landrum et al.^50^	https://ftp.ncbi.nlm.nih.gov/pub/clinvar/vcf_GRCh37/
CiVIC database (v5.1.0)	Griffith et al.^49^	https://civicdb.org/releases/main
Cancer Knowledgebase (CKB)	Patterson et al.^59^	https://ckb.genomenon.com/gene/grid
OncoKB database	Chakravarty et al.^58^	https://www.oncokb.org
CADD scores (v1.6)	Rentzsch et al.^51^	https://cadd.gs.washington.edu/download
PhyloP 100-way	Pollard et al.^52^	dbNSFP v4^76^
TCGA somatic mutations & RNA-seq data	GDC Data Portal	https://portal.gdc.cancer.gov/
HMF somatic mutations & RNA-seq data	Priestley et al.^2,77^	Data request DR-391
CPTAC somatic mutations & proteomics data	Geffen et al.^78,79^	https://pdc.cancer.gov/pdc/cptac-pancancer
TF link	Liska et al.^80^	https://tflink.net/download/
MSigDB signatures	Subramanian et al.^81^	https://www.gsea-msigdb.org/gsea/msigdb/human/collections.jsp
PTMSigDB signatures	Krug et al.^82^	https://github.com/nicolerg/ssGSEA2/tree/master/db/ptmsigdb
AACR GENIE Biopharma Collaborative	AACR Project GENIE Consortium^12^	https://genie.cbioportal.org/; https://www.synapse.org/Synapse:syn27056700
TrainHMM	This paper	https://zenodo.org/records/21430016
Coordinates of significant regions in cancer genes	This paper	https://zenodo.org/records/21430016
Software and algorithms		
ChimeraX (v1.8)	Goddard et al.^83^	https://www.cgl.ucsf.edu/chimerax
FoldX Suite (v5.1)	Delgado et al.^53^	https://foldxsuite.crg.eu/
DSSP (v0.9.9)	Carter et al.^84,85^	https://ssbio.readthedocs.io/en/latest/instructions/dssp.html
Biopython (v1.81)	Cock et al.^86^	https://pypi.org/project/biopython/
MATLAB (R2024a)	MathWorks	https://www.mathworks.com/products/matlab.html
lassoglm	MATLAB Statistics and Machine Learning Toolbox (v24.1)	https://www.mathworks.com/help/stats/lassoglm.html
NetStart2.0	Nielsen et al.^87^	https://services.healthtech.dtu.dk/services/NetStart-2.0/
TRISTAN (v1.0.0)	Clauwaert et al.^88^	https://github.com/TRISTAN-ORF/TRISTAN
HotSpot3D (v0.6.0)	Niu et al.^54^	https://github.com/ding-lab/hotspot3d
OncodriveCLUSTL	Arnedo-Pac et al.^55^	https://bbglab.irbbarcelona.org/oncodriveclustl/analysis
ActiveDriver (v1.0.0)	Reimand et al.^56^	https://cran.r-project.org/web/packages/ActiveDriver/index.html
ssGSEA2 (v1.0.0)	Krug et al.^82^	https://github.com/nicolerg/ssGSEA2
glmnet (v4.1.8)	Friedman et al.^89^	https://glmnet.stanford.edu/
commons-math (v3.6.1)	Apache Commons Mathematics Library	https://commons.apache.org/proper/commons-math/
numpy (v2.0.0)	NumPy Python Library	https://numpy.org
scikit-learn (v1.5.1)	Scikit Learn Python Library	https://scikit-learn.org/stable/
survminer (v0.5.0)	SurvMiner R package	https://github.com/kassambara/survminer
survival (v3.7.0)	Survival R package	https://cran.r-project.org/web/packages/survival/
CUDA Toolkit (v12.5.40)	NVIDIA	https://developer.nvidia.com/cuda-downloads
Drivers for CUDA	NVIDIA	https://www.nvidia.com/Download/index.aspx
JCuda (v10.1.0)	JCuda Java Library	http://www.jcuda.org/downloads/downloads.html
Other		
GeForce RTX3090, RTX A5000	NVIDIA	RTX3090, A5000
A10 and A100 Tensor Core GPU	NVIDIA	Tensor A10, A100
Interactive website for visualizing functional maps	This paper	https://oncofold.org/

## Highlights

- A proteo-genomics framework maps significantly mutated regions in 173 cancer genes
- Regions align with functional domains, structural constraints, and mechanisms
- Functionally related cancer genes show similar patterns of significant regions
- Regions prioritize sparsely annotated mutations for clinical interpretation

## Notations

ℓ: total number of substitution positions in the gene
s: number of states in the HMM
*s* ^(*k*)^: number of states in solution k of an HMM
τ: index at which counts $n_{i}$ are capped during the training of the HMM
γ: probability offset of the baseline state
ν: variance of the log-normal component in the Poisson-lognormal distribution
$e_{i}$: predicted effect of the mutation in position i based on AlphaMissense (score within [0,1])
$m_{i}$: background mutability of position i, depending on its sequence context
$n_{i}$: observed number of mutations in position i
${\hat{\omega }}_{i}$: most probable state of position i
$p_{i,j}$: probability of observing count $n_{i,j}$ under a Poisson-lognormal distribution with $\theta_{i,j}$
$\theta_{i,j}$: expected number of mutations at position i in state j
$\omega_{i,j}$: probability that position i is assigned to state j
$\vartheta_{i,j}$: summarized weights of position i in summarized state j (based on state similarity)
$\phi_{i,j}$: summarized weights of position i in summarized state j (position similarity)
${\tilde{e}}_{i},{\tilde{m}}_{i},{\tilde{n}}_{i},{\tilde{\omega }}_{i,j}$: variables sorted by the predicted effect $e_{i}$ instead of the genomic index
$f_{j}\left(e_{i}\right)$: state-specific mapping function, linking protein effect scores to mutation rates
$\alpha_{i,j}$: probability of state j in position i, after observing states in positions 1,…,i-1
$\beta_{i,j}$: probability of state j in position i, after observing states in positions i+1,…,ℓ
$\Omega_{i,j}$: cumulative weight percentile of position i in state j
$\pi_{j}$: prior probability of state j
A: s×s transition matrix from state j to $j^{\prime }$
ℳ: positions of missense mutations in {1,…,ℓ}
𝒩: positions of nonsense mutations in {1,…,ℓ}
L: log likelihood of an HMM
$L_{s,\gamma ,\tau ,a,b}$: likelihoods of solutions with parameters s,γ,τ in iteration a with initialization b
$L_{s,\gamma ,\tau }$: set of likelihoods of HMMs trained with parameters s,γ,τ
${\tilde{L}}_{s,\gamma ,\tau }$: highest partition of likelihoods within $L_{s,\gamma ,\tau }$
$L_{\text{opt}}$: optimized solutions after iterative selection (second best solution: $L_{\text{opt}}^{\left(2\right)}$, etc)
$dist\left(L,L^{\prime }\right)$: distance (number of standard deviations) between sets of likelihoods L and $L^{\prime }$
$P\left(Y_{\text{Poisson}}=n|\lambda \right)$: point probability of a Poisson distribution with rate λ
$P\left(Y_{\text{PLN}}=n|\theta ,\nu \right)$: point probability of a Poisson-lognormal distribution with mean θ and ν
$f_{\text{norm}}(\lambda |\theta ,\nu )$: probability density function of a normal distribution with mean θ and variance ν
$f_{\log\text{-norm}}(\lambda |\theta ,\nu )$: probability density function of a lognormal distribution, logλ normally distributed
$f_{\exp}(d|\lambda )$: probability density function of the exponential distribution
