# Supplementary material for: Proteo-genomics-guided interpretation of somatic mutations in cancer genomes
Source: Cancer Cell. Author manuscript; Available in PMC 2026 Oct 18. (PMC13676781; doi:10.1016/j.ccell.2026.07.017)
Supplement: Supplementary file 1 [file NIHMS2201278-supplement-1.pdf]

SUPPLEMENTARY FIGURES AND LEGENDS

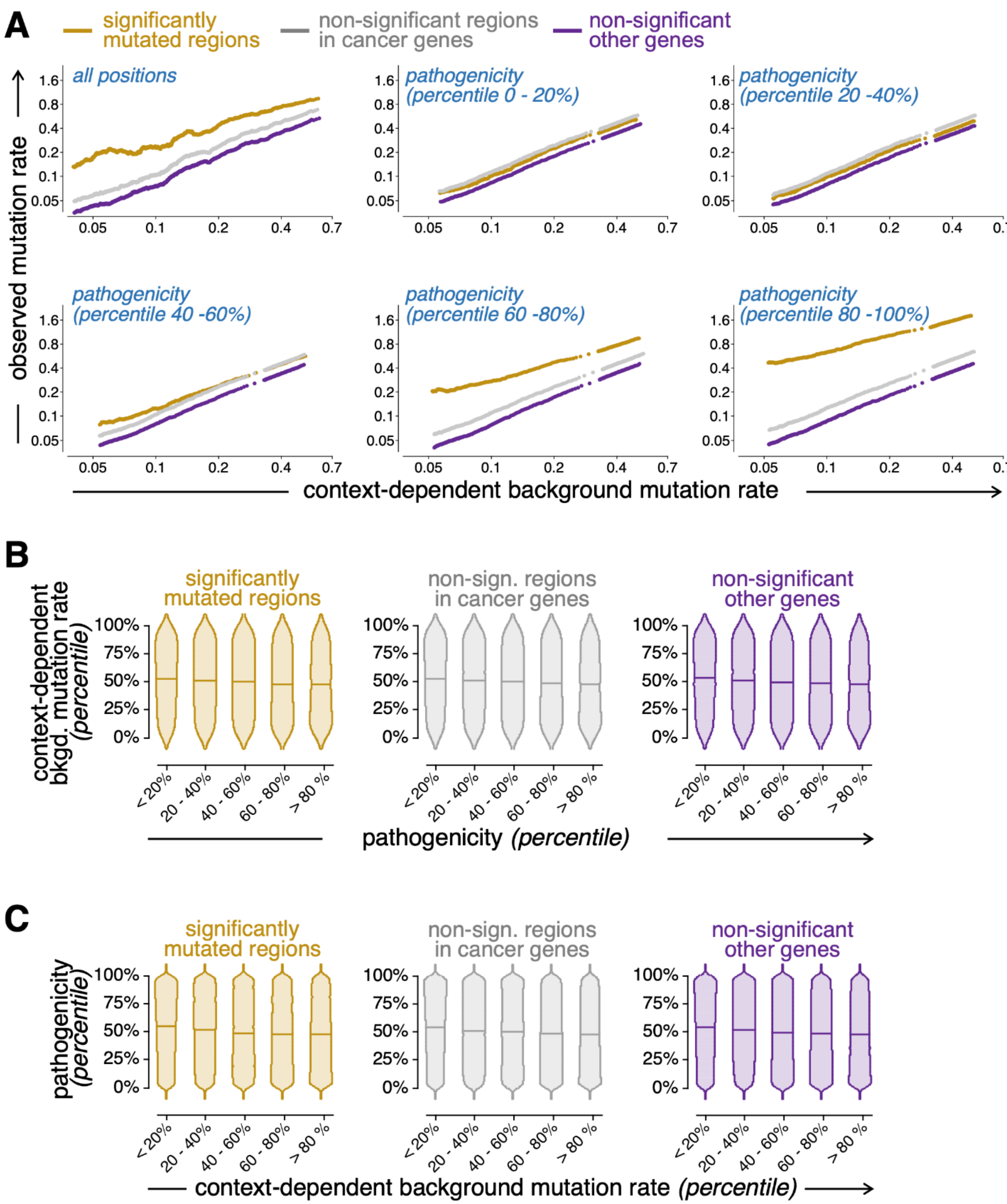

Figure S1. Mutation rates differ between genomic sequences, unrelated to biological and structural effects, related to Figure 1.

(A) To systematically characterize the spatial distribution of mutations in cancer genes, we analyzed mutation data of 139,818 tumor patients (see details of the cohort in STAR Methods). For each genomic position, we counted the number of mutations (y-axis) and determined the background mutation rate of its surrounding genomic

sequence context. We then plotted the average number of mutations per position (y-axis) as a function of its context-dependent background mutation rate (x-axis), using a sliding-window approach (10% width). They exhibited a linear relationship, both in significantly (yellow) and non-significantly mutated regions (gray) of cancer genes. We observed a similar relationship in non-significantly mutated genes (purple). We repeated the same analyses for positions with different effects on protein structures (20% percentile bins; light blue labels) and found a consistent, linear relationship between the number of mutations per position (y-axis) and their background mutation rates (x-axis).

(B, C) We examined whether pathogenicity of mutations depended on their surrounding genomic context. (B) We grouped mutations based on their pathogenicity scores (x-axis; 20% percentile bins) and visualized the distribution of the context-dependent background mutation rate within each group as a violin plot (y-axis, percentile). (C) Vice versa, we grouped mutations based on their context-dependent background mutation rates (20% percentile bins) and visualized the distribution of their pathogenicity scores as a violin plot (y-axis, percentile). Violin plots are shown separately for significantly (yellow) and non-significantly (gray) mutated regions in cancer genes, as well as for non-significantly mutated genes (purple). Horizontal lines within each violin plot indicate the median of each distribution. Distributions were across percentile bins, indicating that pathogenicity and genomic contexts of mutations are largely independent from each other.

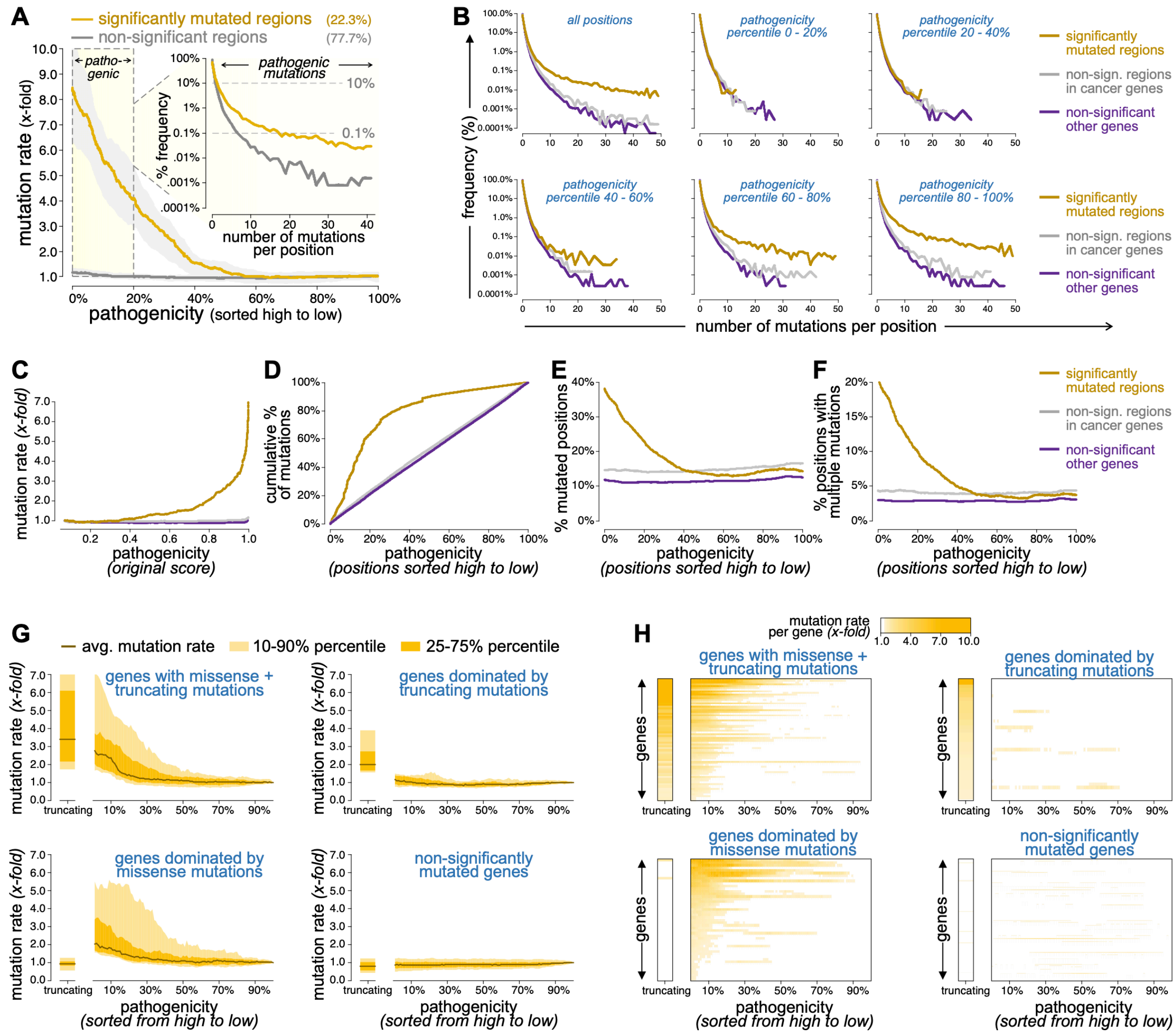

Figure S2. Spatial distributions of mutations in significant vs. non-significant regions, related to Figure 1. (A) We plotted mutation rates (y-axis; x-fold change to baseline) against the pathogenicity of mutations (x-axis). Gray shadings show the variation of these analyses across cancer genes. Differences between significant (yellow) vs. non-significant regions (gray) manifested primarily in positions with strong pathogenicity (dashed box). Histograms (insets) display the distribution (y-axis) of mutation counts (x-axis) of these positions, with differences between significant (yellow) vs. non-significant positions (gray) manifesting in long distribution tails.

(B) We counted the number of mutations per position across 139,818 tumor patients. Histograms visualize the frequency (y-axis) of each mutation count (x-axis) in significant (yellow) vs. non-significant (gray) regions of cancer genes – either for all positions combined (top left) or separately for positions with different pathogenicity scores (20% percentile bins, light blue labels). As a reference, we included mutation counts of non-cancer genes (purple). These analyses revealed that differences in mutation counts of significant vs. non-significant regions manifest in their long distribution tails, requiring accurate modeling of ultra-rare events. These differences were particularly prominent in those positions with substantial pathogenicity.

(C) To ensure that the patterns shown in (A) were not driven by differences in the pathogenicity of significant vs. non-significant regions, we repeated the same analyses using the original pathogenicity score (x-axis) instead of percentiles. We plotted mutation rates (y-axis; fold change relative to baseline) against their original pathogenicity scores (x-axis), using a sliding window approach (10% width). The results were similar to those in (A), confirming

that our observations reflect differences in the spatial distribution of mutations in significant regions, rather than differences in their pathogenicity.

(D) We sorted positions based on their pathogenicity scores (percentile, x-axis) and plotted the cumulative fraction of mutations falling into these positions (ROC curve). In significant regions (yellow), mutations accumulated in positions with pathogenicity. In non-significant regions of the same genes (gray), pathogenicity had limited influence on the distribution of mutations (close match to the diagonal).

(E-F) To ensure that the differences in (A) were not driven by a few positions with high mutation counts, we plotted the pathogenicity scores (x-axis) against the fraction of mutated positions (y-axis, E) and the fraction of positions with multiple mutations (y-axis, F), using a sliding window approach (10% width). We observed similar differences as in Figure S2A, confirming that pathogenicity affected the overall spatial distribution of mutations in significant regions, rather than a few hotspot positions. In non-significant regions of the same genes, mutations were distributed independently of their pathogenicity.

(G-H) We noticed that the relationship between mutation rates and pathogenicity differed substantially across individual genes. We thus examined the mutation rate of each cancer gene (y-axis; x-fold change to baseline) as a function of the pathogenicity score (x-axis). This analysis distinguished four main clusters: (i) cancer genes with both missense and truncating mutations (e.g., *TP53*, *PTEN*; top left), (ii) cancer genes with truncating mutations only (e.g., *RB1*, *APC*; top right), (iii) cancer genes with missense mutations only (e.g., *CBL*, *SPOP*; bottom left), and (iv) non-significantly mutated genes with missense and truncating mutation rates similar to baseline (bottom right). (G) Black lines indicate the average mutation rate in each cluster (y-axis), as a function of the pathogenicity score (x-axis). Orange boxes span the interquartile range and yellow boxes span the 10-90% percentile range across genes. (H) We visualized the mutation rate of individual genes in each cluster (rows), as a function of the pathogenicity score (x-axis). Colors indicate the x-fold change of the mutation rate, relative to the baseline mutation rate of its positions without substantial pathogenicity (white: no change; dark yellow: 10-fold change).

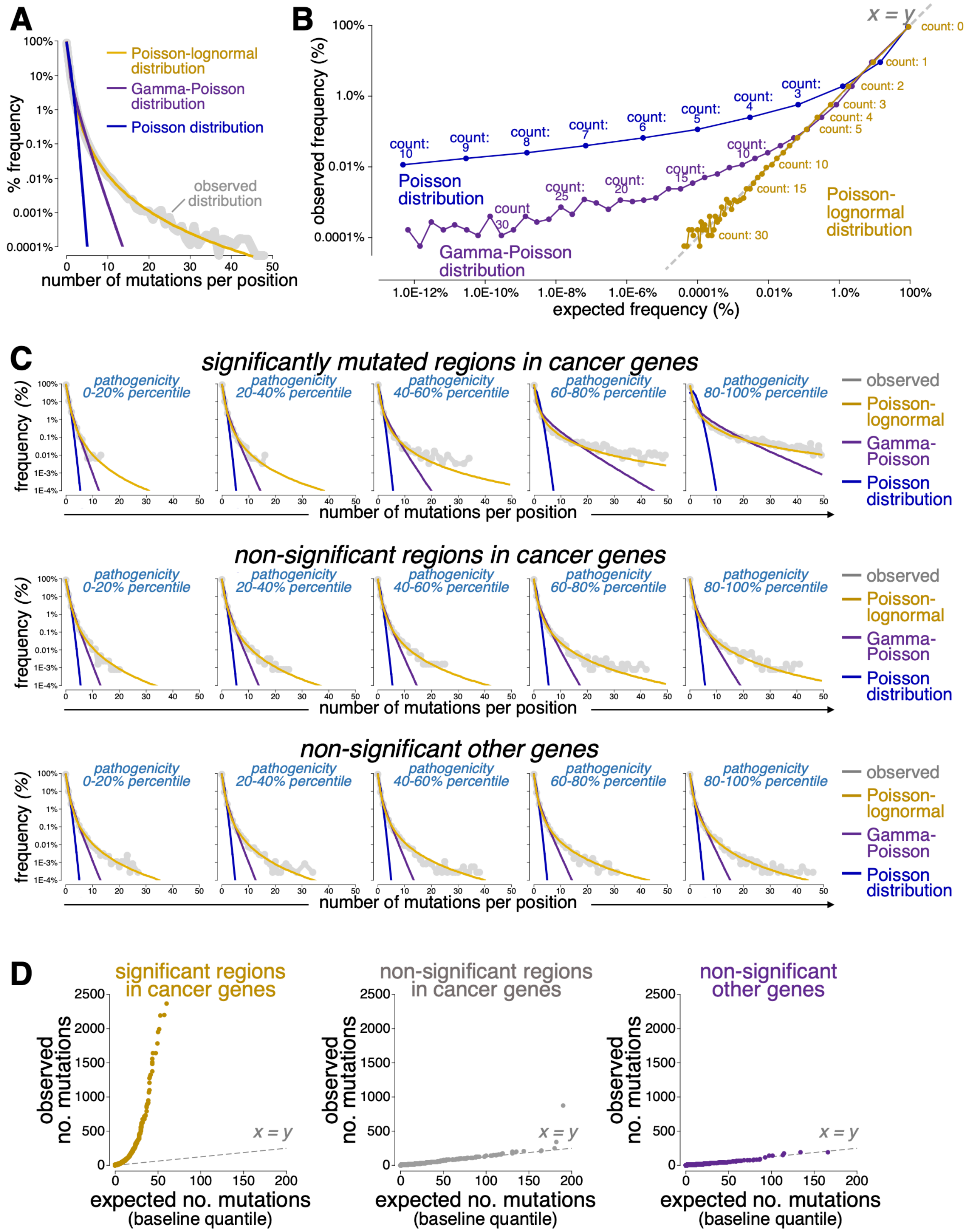

Figure S3. Statistical modeling of fine-resolution mutation data at a population scale , related to Figure 1.
(A) Given the population-scale of our analysis, we employed a Poisson-lognormal distribution (yellow) to model mutation events in our cohort (gray). For reference, we included Poisson (blue) and Gamma-Poisson (purple)

distributions, which have been the standard models of many cancer genomics studies. The Poisson-lognormal model allows for a larger variance, which is critical to accurately model mutation counts in population-scale datasets and capture the long distribution tail related to significant regions in cancer genes (see statistical details in the STAR Methods).

(B) For each mutation count, we plotted its observed frequency (i.e., the fraction of positions with this mutation count; x-axis) against the expected frequency (i.e., the point probability of the fitted distribution model; y-axis). For the Poisson-lognormal model (orange), we observed a close match between its expected (x-axis) and observed (y-axis) frequencies (diagonal dashed line). Other distribution models (purple, blue) deviated from the diagonal, signifying that these models underestimate the variance in the data.

(C) Histograms visualize the frequency (y-axis) of each mutation count (x-axis) in the observed data (gray) and each distribution model. We performed these analyses separately for positions with different pathogenicity scores (20% percentile bins, left to right), and depending on their location in significantly (top) and non-significantly mutated regions of cancer genes (middle). For reference, we also analyzed mutation counts in non-significantly mutated genes (bottom). These analyses show that the Poisson-lognormal distribution accurately fits, both in significantly and non-significantly mutated regions of cancer genes, while other distribution models tend to underestimate the variance of mutation counts.

(D) Q-Q plots visualize the fit of the observed data to the background distribution of the baseline state of our model (expected vs. observed quantiles). The diagonal (dashed gray line) reflects a perfect fit between the observed and expected quantiles. We performed this analysis separately for significant (yellow, left) and non-significant regions in cancer genes (gray, middle), as well as non-significantly mutated genes (purple, right). Our analyses show that the baseline distribution accurately fits the observed mutation counts in non-significant regions in cancer genes and non-significantly mutated genes. By contrast, significantly mutated regions in cancer genes deviate from the baseline distribution, indicating that additional biological factors shape their mutation patterns.

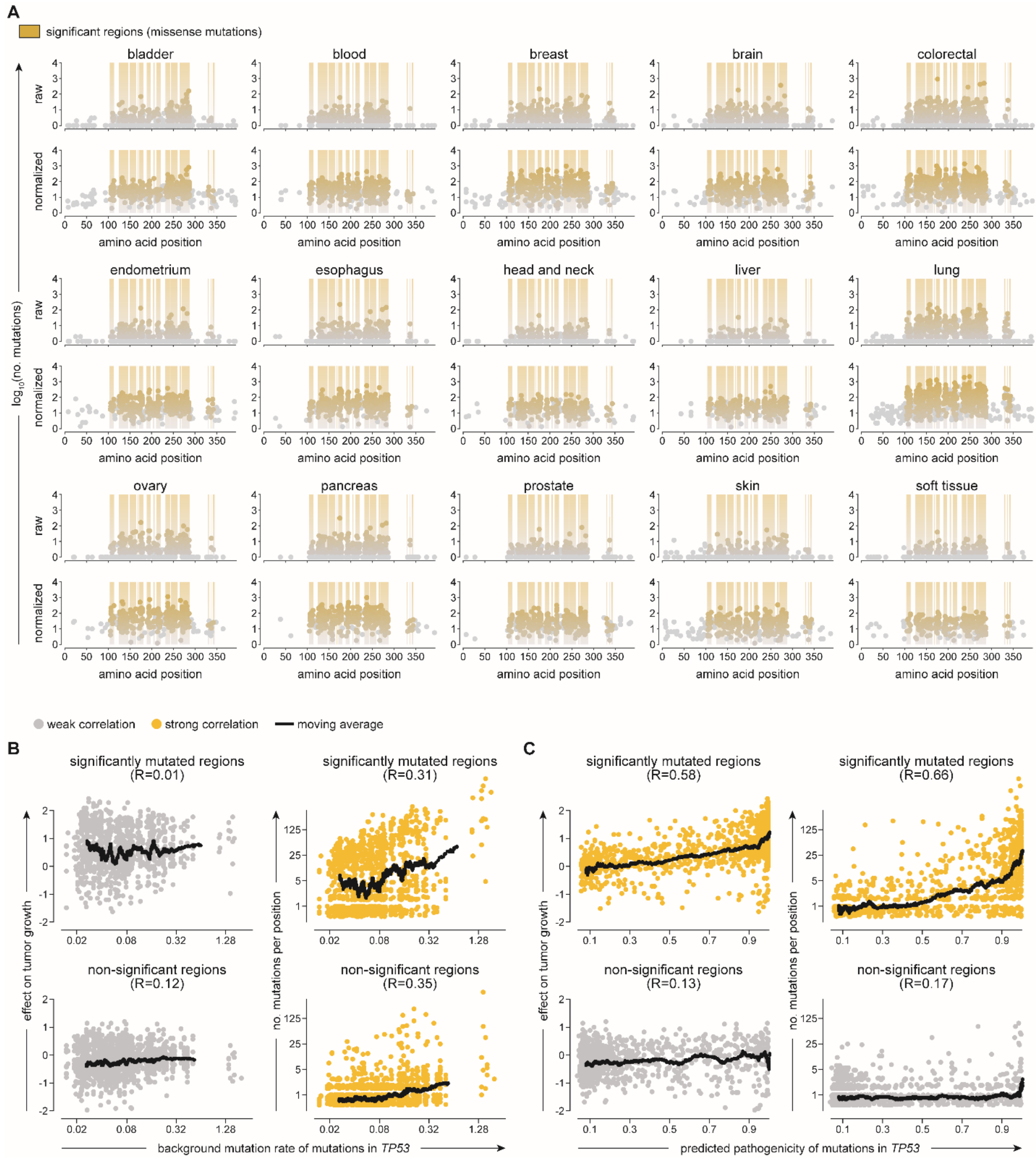

Figure S4. Experimental validation of the background model in *TP53*, related to Figure 1.

(A) To demonstrate that pseudo-hotspots of *TP53* observed in raw mutation counts largely disappear after correcting for covariates, we plotted the number of mutations (log-scale) before (raw, top) and after (normalized, bottom) background mutation rate correction in the indicated cancer type (y-axis) against their positions along the gene (x-axis) for each cancer type. We included only cancer types with more than 500 mutations across the gene.

(B) For each *TP53* mutation, we compared the background mutation rate predicted by our model with the experimentally measured effects on tumor cell growth in mutagenesis data (z-score; left) and the observed number of mutations per position (right). Orange dots indicate relationships with strong correlations, gray dots indicate weak correlations, and black lines show moving averages. Spearman's rank correlation coefficients (R)

are annotated for each plot. Background mutation rates correlated with the raw number of mutations per position, but not with experimentally measured effects on tumor cell growth. Thus, raw mutation counts do not provide a direct readout of tumorigenic selection unless they are adjusted for local background mutation rates.

(C) For each *TP53* mutation, we compared predicted pathogenicity (AlphaMissense) with the experimentally measured effect on tumor cell growth (z-score; left) and with the observed number of mutations per position (right). In significantly mutated regions, predicted pathogenicity correlated strongly with functional effects and mutation counts. In contrast, mutations in non-significant regions showed only weak correlations, suggesting that many mutations in these regions have limited effects on tumor cell growth, even when predicted to be pathogenic. This difference corroborates the ability of our model to identify regions in which recurrent mutations are more likely to reflect tumorigenic selection rather than background mutational processes.

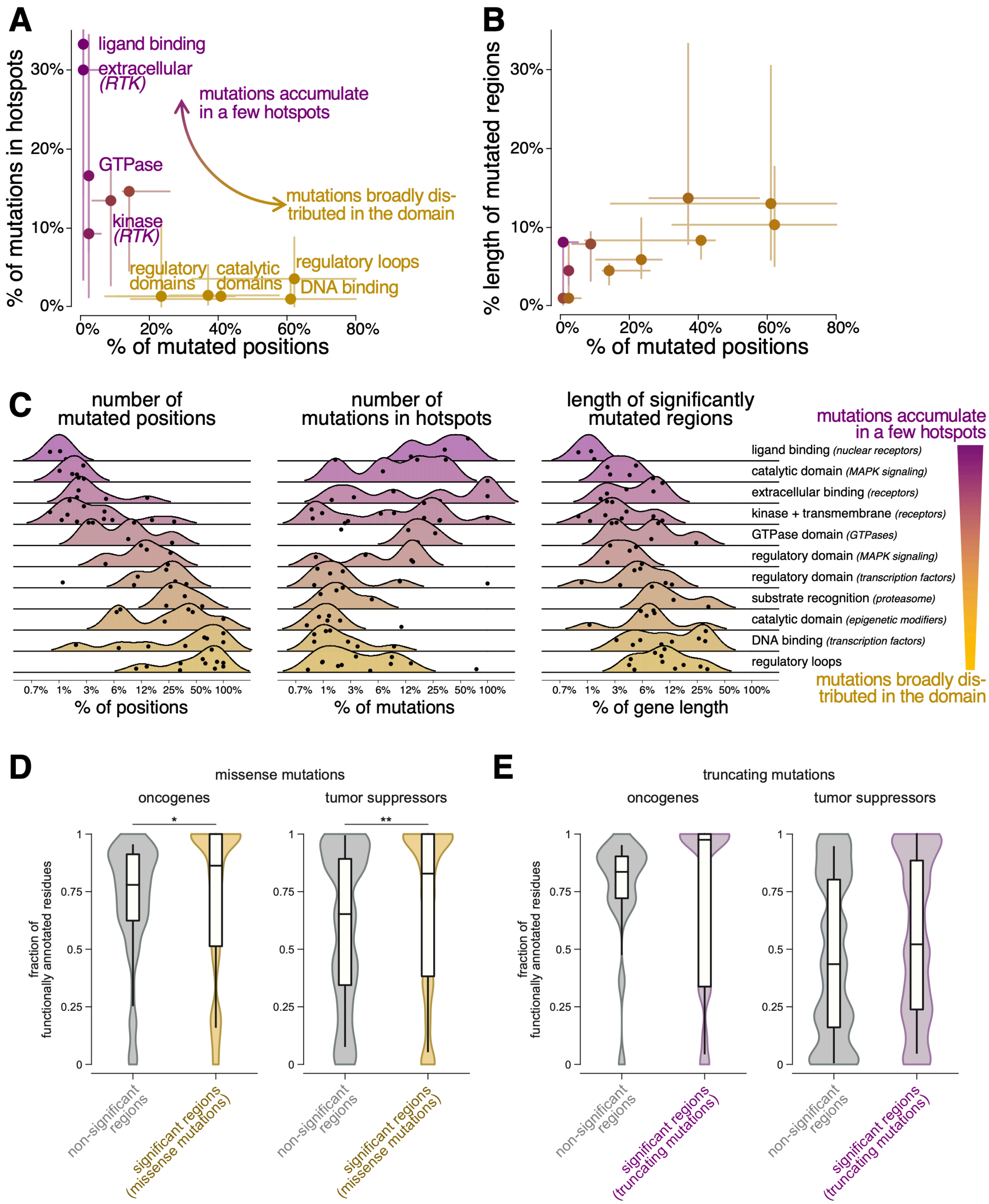

Figure S5. Lengths of significant regions and enrichment of functionally annotated residues, related to Figure 1.

(A-C) We sorted cancer genes into functional groups (same as those shown in Figures 1D-1F) and characterized the mutation patterns in each group based on (i) the fraction of mutated positions in each gene, (ii) the fraction of mutations falling into hotspot positions, and (iii) the fraction of the gene covered by significantly mutated regions

(% of gene length). (A, B) For each functional group, we plotted the fraction of mutated positions (x-axis) against the fraction of mutations falling into hotspots (y-axis, A) and the fraction covered by significantly mutated regions (y-axis, B). Dots indicate the median of each group, and lines span the 10/90% percentile range. (C) Density plots visualize the distribution of the fraction of mutated positions (left), the fraction of mutations in hotspots (middle), and the relative length of significantly mutated regions (right) in each functional group (rows). Dots display values of individual genes in each functional group.

These analyses show that shorter significant regions are typically associated with changes to specific interaction sites (e.g., binding of GTP or small ligands), where mutations accumulate in a few hotspots. Longer significant regions, by contrast, are linked to larger molecular interactions (e.g., DNA binding, substrate recognition, catalytic activity), where mutations are distributed more broadly, resulting in higher fractions of mutated positions and smaller fractions of hotspots.

(D-E) We compared the fraction of functionally annotated residues in significantly mutated regions [orange and purple for missense (D) and truncating (E) mutations, respectively] vs. non-significant regions (gray). We included only genes for which the average normalized mutation count in significant regions was greater than that in non-significant regions. We classified genes as oncogenes or tumor suppressors based on the Cancer Gene Census and OncoKB databases. Violin plots visualize the distribution of fractions, horizontal lines indicate the median, boxes span the interquartile range, and whiskers extend to the 10/90th percentiles of each distribution. Statistically significant differences are denoted with asterisks (*P < 0.05, **P < 0.01; two-sided Wilcoxon rank-sum test).

These results indicate that residues within significantly mutated regions are enriched for functional annotations compared to non-significant regions, supporting the biological relevance of the identified signals.

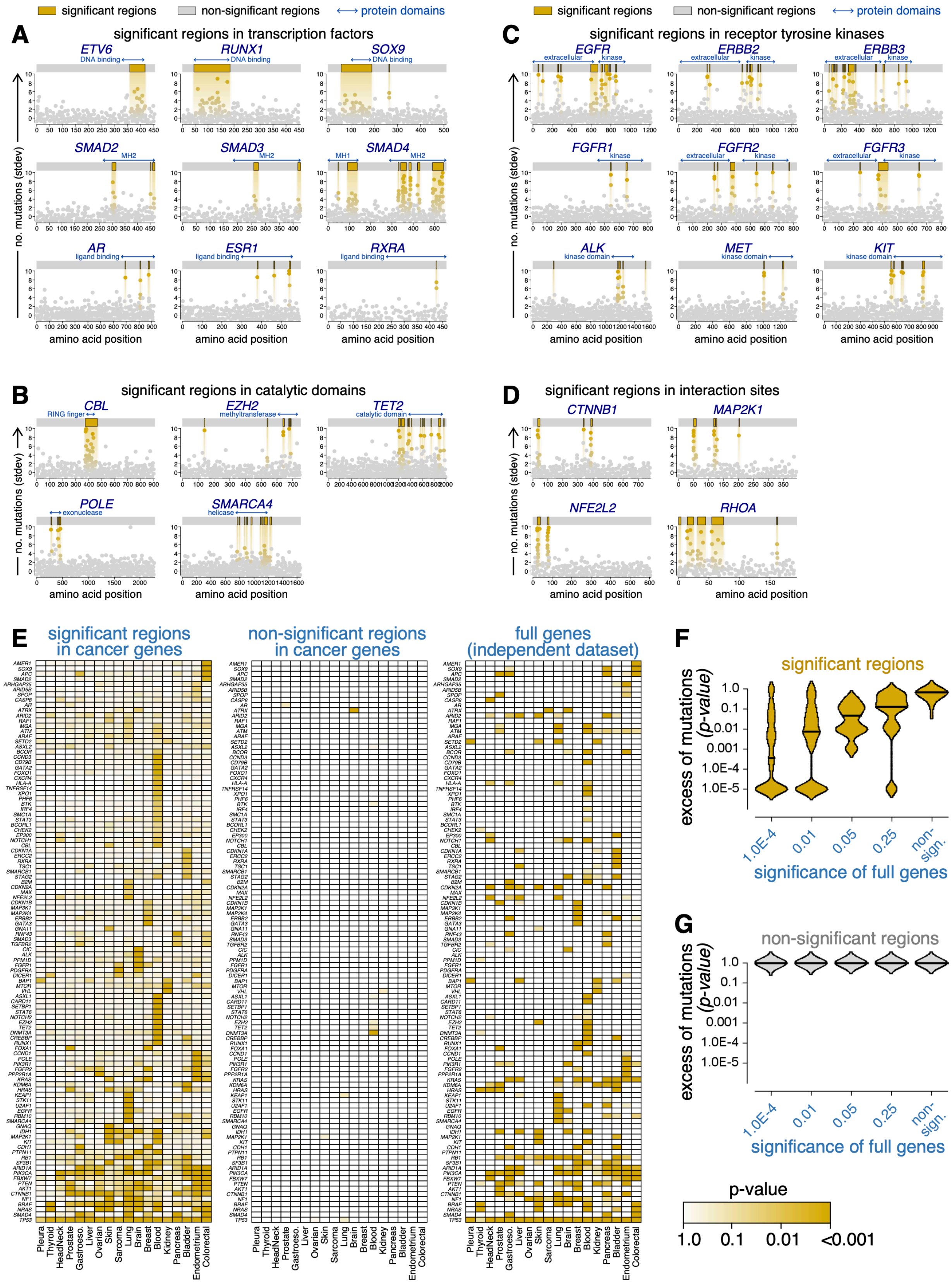

significant regions
non-significant regions
protein domains
A
significant regions in transcription factors
ETV6
RUNX1
SOX9
DNA binding
SMAD2
SMAD3
SMAD4
MH1
MH2
AR
ESR1
RXRA
ligand binding
no. mutations (stdev)
amino acid position
B
significant regions in catalytic domains
CBL
EZH2
TET2
RING finger
methyltransferase
catalytic domain
POLE
SMARCA4
exonuclease
helicase
C
significant regions in receptor tyrosine kinases
EGFR
ERBB2
ERBB3
extracellular
kinase
FGFR1
FGFR2
FGFR3
ALK
MET
KIT
kinase domain
D
significant regions in interaction sites
CTNNB1
MAP2K1
NFE2L2
RHOA
E
significant regions in cancer genes
non-significant regions in cancer genes
full genes (independent dataset)
Pleura
Thyroid
HeadNeck
Prostate
Gastroeso.
Liver
Ovarian
Skin
Sarcoma
Lung
Brain
Breast
Blood
Kidney
Pancreas
Bladder
Endometrium
Colorectal
F
significant regions
excess of mutations (p-value)
1.0
0.1
0.01
0.001
1.0E-4
1.0E-5
significance of full genes
1.0E-4
0.01
0.05
0.25
non-sign.
G
non-significant regions
p-value
1.0
0.1
0.01
<0.001

Figure S6. Significant regions localize to functional domains and are mutated in specific cancer types, related to Figure 1.

(A-D) To visualize the spatial distribution of mutations in cancer genes, we plotted the number of mutations per genomic position (y-axis) against their location within each gene (x-axis). To account for the background fluctuation in mutation rates, we transformed mutation counts into standard deviations from the mean (z-scores). Mutations within significant regions are colored in orange, those in non-significant regions are colored in gray. At the top of each plot, we annotated the location of relevant subdomains (blue double-headed arrows). These analyses highlight the overlap of significant regions and functional domains in cancer genes, offering insights into their roles in tumorigenesis.

(E) For each gene (row) and cancer type (column), we quantified whether their significant regions contained more mutations than by chance (left) (white: non-significant; brown: significant excess of mutations). We repeated the same analyses for non-significantly mutated regions in the same cancer genes (middle). As a reference, we used data from a previous pan-cancer study that evaluates in which cancer type each gene has mutational significance (entire gene in aggregate; right).

(F, G) Violin plots show the distribution of mutational significance values (y-axis) in significant (F, yellow) and non-significant regions (G, gray) of cancer genes, depending on the mutational significance of their full cancer gene in a previous pan-cancer study (x-axis). Vertical lines indicate the median of each distribution. These analyses demonstrate that many significant regions harbor an increased number of mutations only in specific cancer types (e.g., the forkhead domain of *FOXA1* is mutated primarily in breast and prostate cancer). In the remaining 79%, cancer genes do not harbor more mutations than expected by chance.

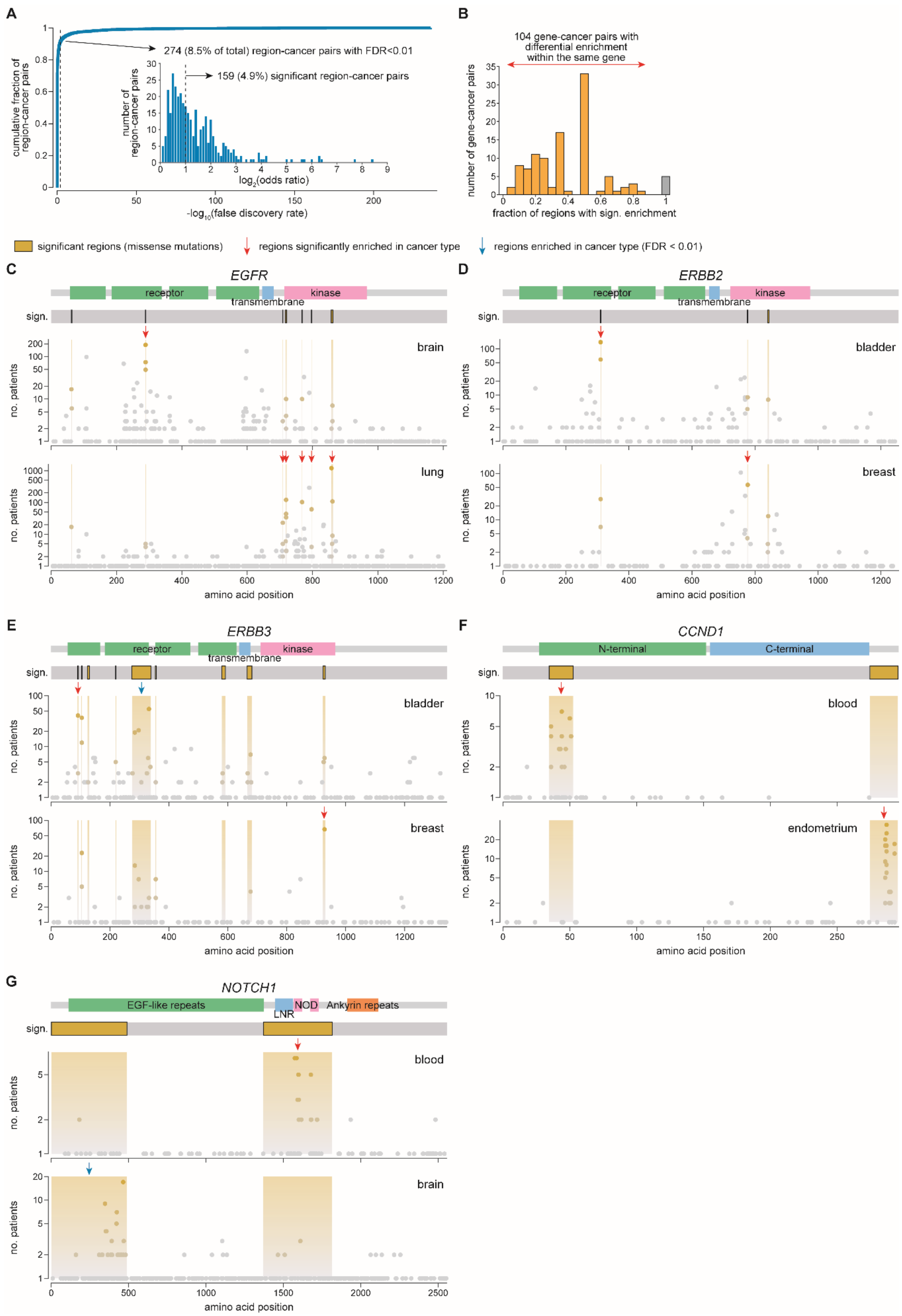

Figure S7. Cancer type-specific patterns of significant regions, related to Figure 1.

(A) For each gene, we assessed whether missense mutations localized to different regions across cancer types. For this, we performed a one-sided Fisher's exact test, testing for cancer type-specific enrichment of individual significantly mutated regions for genes harboring multiple significant regions in the overall cohort. We then

adjusted p-values for multiple comparisons using the Benjamini-Hochberg method to obtain false discovery rates (FDR; x-axis). We identified 274 region-cancer pairs with FDR < 0.01. To reduce spurious associations, we applied an additional filter requiring an odds ratio > 2 (inset x-axis), resulting in 159 significant pairs, representing 4.9% of all region-cancer pairs tested.

(B) We next assessed whether significantly mutated regions within the same gene showed differing cancer type-specific distributions. For each gene-cancer pair, we calculated the fraction of significant regions showing significant cancer type enrichment (i.e., significant region-cancer pairs) among the total number of significant regions. We identified 104 gene-cancer pairs with a fraction <1 (orange), indicating heterogeneity across regions within the same gene.

(C-G) For representative cancer genes with heterogeneity across regions within the same gene (*EGFR*, *ERBB2/3*, *CCND1*, and *NOTCH1*), we plotted the number of patients with mutations (y-axis) in the indicated cancer type (upper right of each plot) against their position along the gene (x-axis). Red arrows above significantly mutated regions indicate regions significantly enriched in a specific cancer type (FDR < 0.01 and odds ratio > 2), whereas blue arrows indicate regions with FDR < 0.01 but odds ratio ≤ 2.

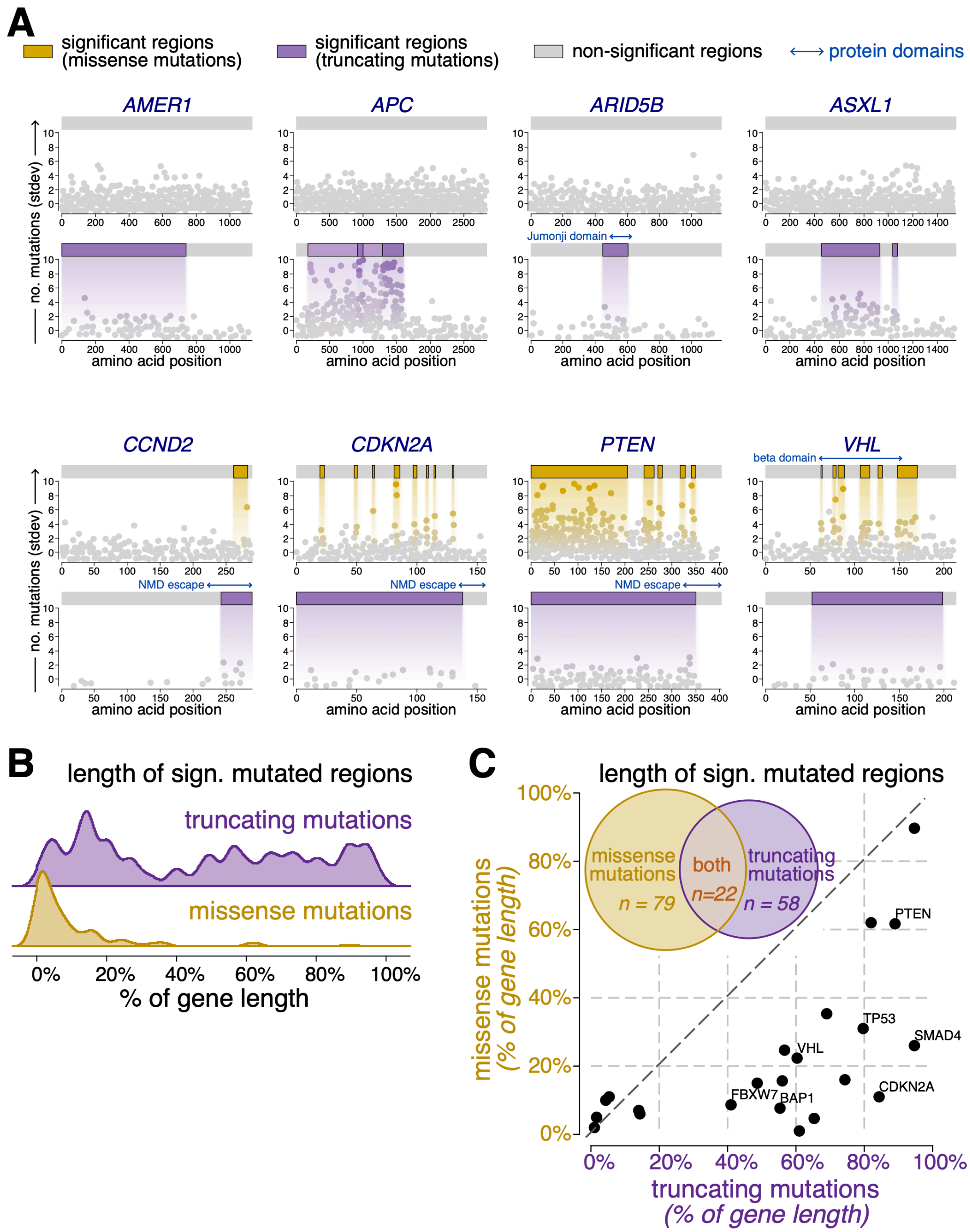

Figure S8. Missense and truncating mutations follow different distribution patterns, related to Figure 1. (A) To compare the distribution of missense (yellow) and truncating (purple) mutations, we plotted the number of mutations per genomic position (y-axis) against their location within each gene (x-axis). To account for background fluctuations in mutation rates, we transformed mutation counts into standard deviations from the mean (z-scores). Mutations in significant regions are colored in orange and purple, those in non-significant regions are colored in gray. At the top, we annotated the locations of relevant functional subdomains and regions associated with nonsense-mediated decay (both indicated by blue double-headed arrows). These analyses showed that missense and truncating mutations often target different regions, even when co-occurring in the same gene.

(B) For each gene, we determined the fraction covered by significantly mutated regions (% of gene length, x-axis). We plotted the distribution of these lengths for truncating (purple, top) and missense (yellow, bottom) mutations as a density plot. These analyses revealed that truncating mutations generally targeted broader regions than missense mutations.

(C) For cancer genes with both truncating and missense mutations, we analyzed the length of significant regions for truncating (x-axis) vs. missense mutations (y-axis). The Venn diagram (inset) visualizes the number of tumor suppressor genes with significant regions related to missense mutations (yellow), truncating mutations (purple), or both (brown).

These analyses revealed that significantly mutated regions exhibit a wide range of lengths, both in oncogenes and tumor suppressor genes. Truncating mutations generally target broader regions than missense mutations and follow an independent distribution pattern, even when co-occurring in the same gene.

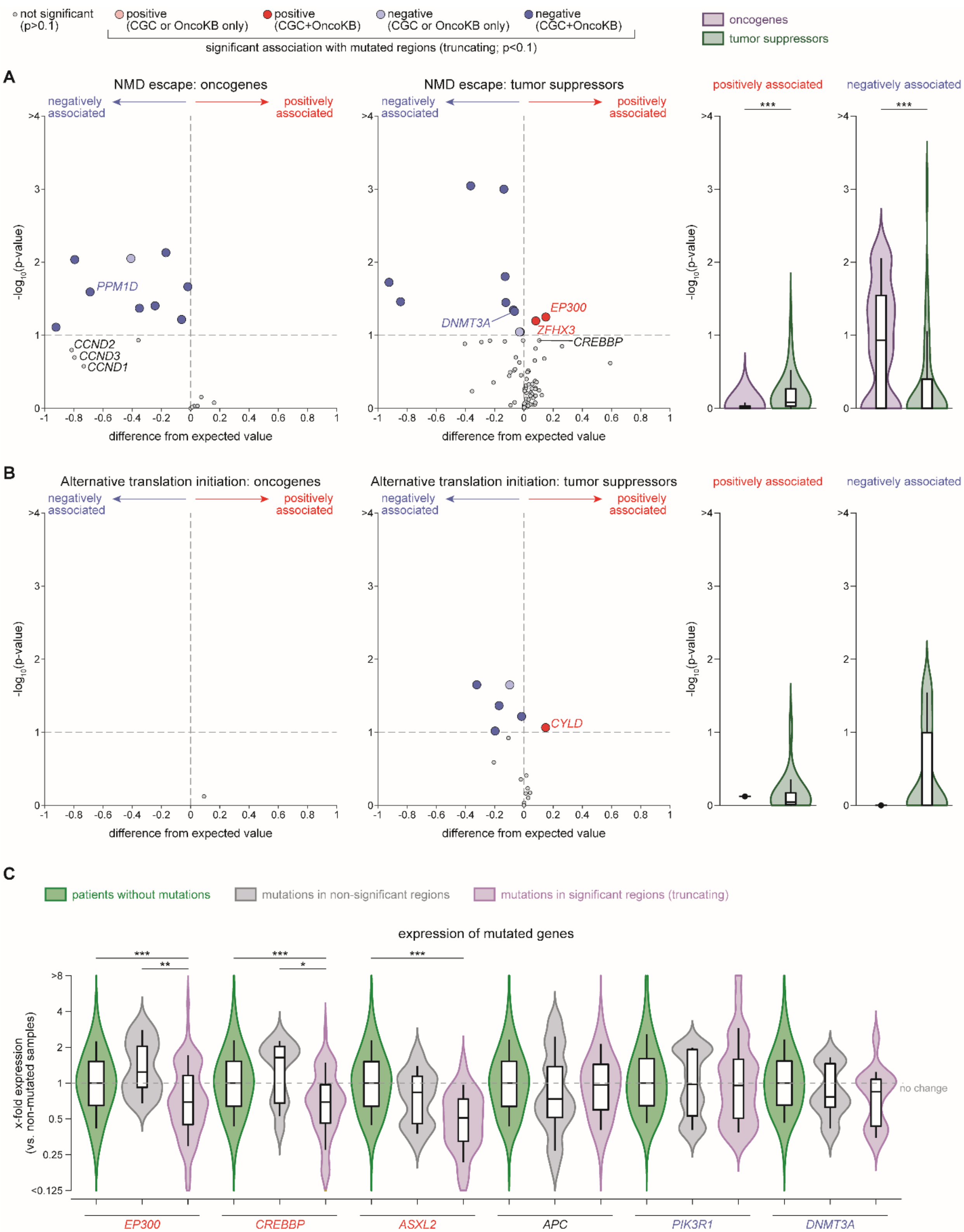

Figure S9. Enrichment of NMD- and ATI-associated regions in significant regions of truncating mutations, related to Figure 2.

(A-B) We quantified the depletion of truncating mutations within significantly mutated regions in C-terminal NMD-escape regions (A) and regions between conventional and alternative translation initiation sites (ATI regions) (B)

using a random permutation test. Enrichment analysis for ATI regions was restricted to genes with predicted alternative start codons. For each gene, we randomly selected regions of the same length as the significantly mutated regions 10,000 times and calculated the weighted fraction of overlap with non-NMD escape regions and non-ATI regions. A one-sided p-value was estimated from the resulting empirical distribution. To reduce spurious associations, we restricted the analysis to the genes for which the average normalized mutation count in significant regions was greater than that in non-significant regions. Volcano plots display the $-\log_{10}$(p-value) from the permutation test (y-axis) versus the deviation from expected values estimated by random permutation (x-axis). Genes positively or negatively associated with C-terminal NMD or ATI (p-value < 0.1) are highlighted in red or blue, respectively. Among these, dark or light colors denote oncogenes/tumor suppressors annotated in both the Cancer Gene Census (CGC) and OncoKB (CGC+OncoKB) or in either database alone (CGC or OncoKB only), respectively. Genes annotated in both databases with a weighted fraction of overlap > 0.25, as well as those discussed in the main text, are labeled. Violin plots show the distribution of $-\log_{10}$(p-value) for each gene, marking the median (horizontal lines), interquartile range (boxes), and 10/90% percentiles (vertical lines) of each distribution. For groups with a single observation (oncogenes in ATI), the individual data point is shown. Statistically significant differences between oncogenes and tumor suppressors are denoted with asterisks (***P < 0.001; two-sided Wilcoxon rank-sum test).

(C) We compared the expression levels of tumor suppressors positively (red; *CREBBP*, *EP300*, and *ASXL2*) or negatively (blue; *PIK3R1* and *DNMT3A*) associated with C-terminal NMD (enrichment p-value < 0.25) across samples without mutations (green) and samples with truncating mutations in significantly mutated regions (purple) or non-significant regions (gray). Genes lacking matched expression and mutation data from at least three independent patients in either the significant or non-significant regions were excluded from the analysis. *APC* was included as an example of a gene with a long last exon, despite showing relatively weaker association with NMD. Violin plots visualize the relative expression of each group (relative to the median expression in non-mutated samples), horizontal lines indicate the median, boxes span the interquartile range, and whiskers extend to the $10/90^{th}$ percentiles of each distribution. Dashed horizontal lines mark the baseline of non-mutated samples for each metric. Statistically significant differences are denoted with asterisks (*P < 0.05, **P < 0.01, ***P < 0.001; Kruskal-Wallis test with Tukey's posthoc correction). These analyses suggested that truncating mutations in NMD-associated regions were associated with lower expression levels, whereas those outside these regions did not exhibit this effect.

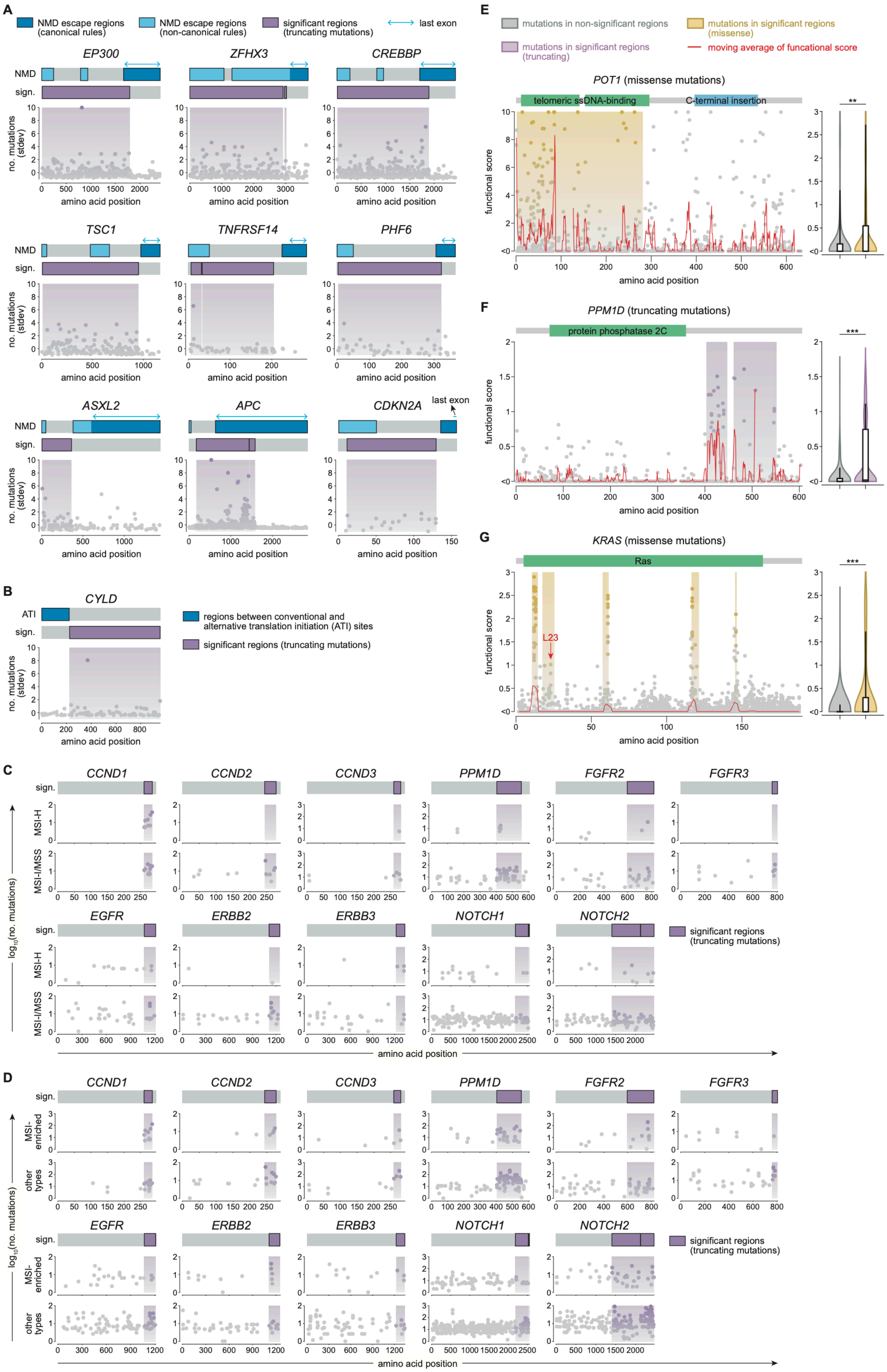
A
NMD escape regions (canonical rules)
NMD escape regions (non-canonical rules)
significant regions (truncating mutations)
last exon
EP300
ZFHX3
CREBBP
TSC1
TNFRSF14
PHF6
ASXL2
APC
CDKN2A
NMD
sign.
no. mutations (stdev)
amino acid position
B
CYLD
ATI
regions between conventional and alternative translation initiation (ATI) sites
significant regions (truncating mutations)
C
CCND1
CCND2
CCND3
PPM1D
FGFR2
FGFR3
EGFR
ERBB2
ERBB3
NOTCH1
NOTCH2
MSI-H
MSI-L/MSS
log10(no. mutations)
D
MSI-enriched
other types
E
mutations in non-significant regions
mutations in significant regions (missense)
mutations in significant regions (truncating)
moving average of funcational score
POT1 (missense mutations)
telomeric ssDNA-binding
C-terminal insertion
functional score
F
PPM1D (truncating mutations)
protein phosphatase 2C
G
KRAS (missense mutations)
Ras
L23

Figure S10. Significant regions overlap with NMD- and ATI-associated regions and support mechanistic inference outside canonical cancer genes and domains, related to Figure 2.

(A-B) For each cancer gene positively associated with C-terminal NMD or ATI (enrichment p-value < 0.25), we plotted the number of truncating mutations (y-axis) against their position along the gene (x-axis). *APC* and *CDKN2A* were included as examples of genes with long and short last exons, respectively, although they showed relatively weaker associations with NMD. To account for the fluctuation in background mutation rates, we normalized mutation counts as standard deviations from the mean (z-scores). At the top of each plot, we annotated the location of NMD escape regions defined by canonical rules (the last exon and penultimate exon rules; dark blue) and non-canonical rules (the coding start site and large exon rules; light blue) (A), as well as regions between conventional and alternative translation initiation sites (blue) (B).

(C-D) We assessed whether C-terminal truncating mutations in oncogenes were related to microsatellite instability (MSI). (C) Spatial distributions of truncating mutations (y-axis) are plotted against amino acid positions (x-axis) for samples annotated as MSI-high (MSI-H) or MSI-intermediate or microsatellite-stable (MSI-I/MSS) (rows). (D) Samples were grouped into tumor types in which MSI is common ("MSI-enriched" / top row: colorectal, endometrial, esophageal, gastric cancer) vs. tumor types in which MSI is less common ("other types" / bottom row). Spatial distributions of truncating mutations (y-axis) are plotted against amino acid positions (x-axis), stratified by tumor-type context (rows). Significantly mutated regions (truncating mutations) are colored in purple. Overall, we observed no consistent differences in the spatial patterns of truncating mutations in samples and tumor types with different MSI annotations.

(E-G) Mutagenesis screens offer a scalable way to measure the functional impact of specific mutations in cancer genes. We compiled experimental mutagenesis data for *POT1* (E), *PPM1D* (F), and *KRAS* (G) (see STAR Methods). For each mutation in these experiments (dots), we plotted its functional scores from the experiment (y-axis) against its genomic position (x-axis). To compare experimental data with the predictions of our computational model (orange and purple shade for missense and truncating mutations, respectively), we assessed whether the computational predictions of our model matched the regions with a high density of functional mutations from the experimental readout, relative to mutations in the rest of the same gene. Red lines represent the moving average of functional scores (centered window, size = 5). The red arrow in the *KRAS* panel indicates KRAS L23, which was identified as part of a significant region and has been reported to mediate KRAS-RAF1 interactions through the RAF1 cysteine-rich domain. Violin plots show the distribution of functional scores, marking the median (horizontal lines), interquartile range (boxes), and 10/90% percentiles (vertical lines) of each distribution. Statistically significant differences between non-significant and significant regions are denoted with asterisks (**P < 0.01, ***P < 0.001; two-sided Wilcoxon rank-sum test). Overall, we observed a close match of experimental readouts and our computational predictions.

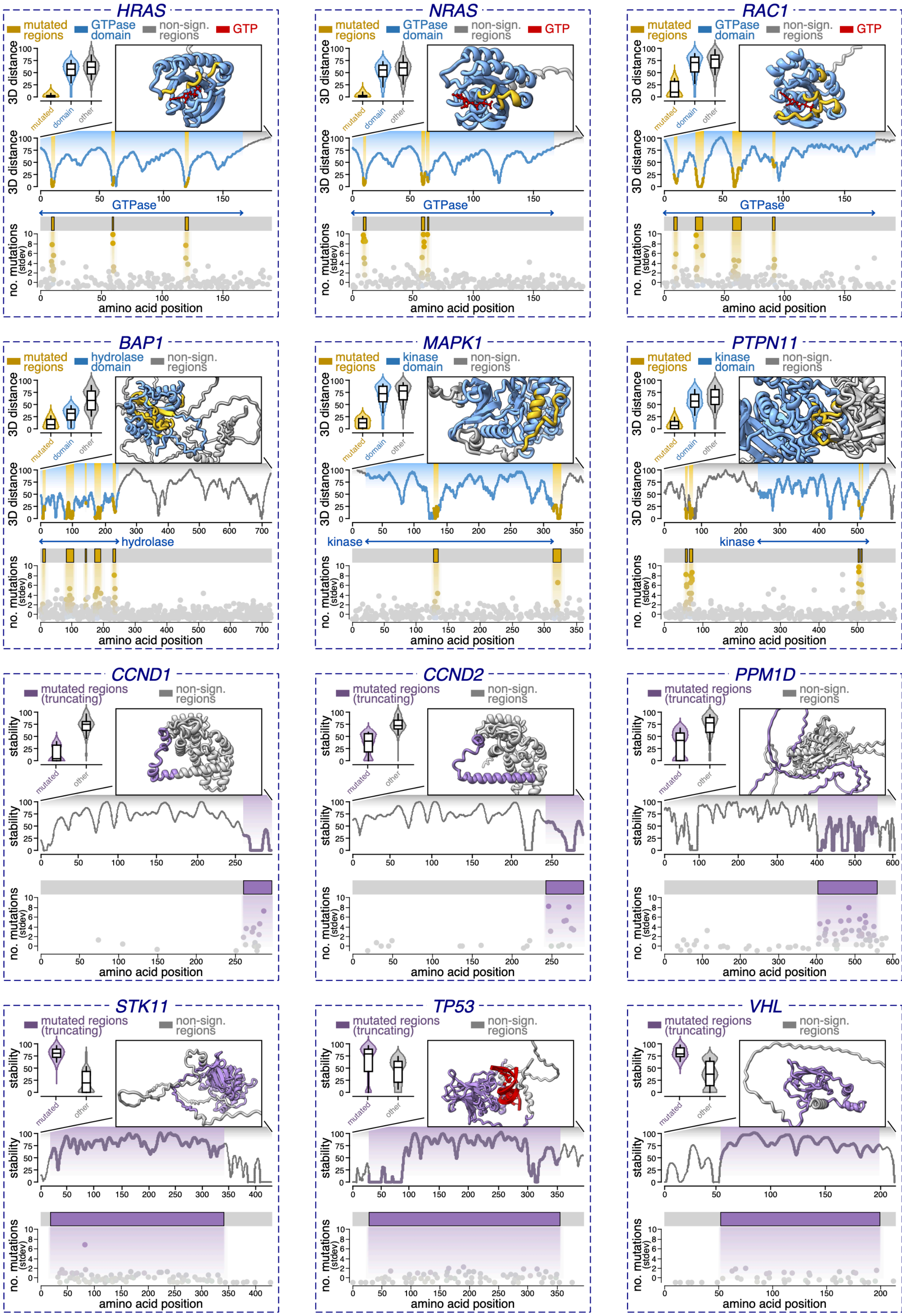
HRAS
mutated regions
GTPase domain
non-sign. regions
GTP
3D distance
mutated
domain
other
GTPase
no. mutations (stdev)
amino acid position
NRAS
mutated regions
GTPase domain
non-sign. regions
GTP
RAC1
mutated regions
GTPase domain
non-sign. regions
GTP
BAP1
mutated regions
hydrolase domain
non-sign. regions
hydrolase
MAPK1
mutated regions
kinase domain
non-sign. regions
kinase
PTPN11
mutated regions
kinase domain
non-sign. regions
kinase
CCND1
mutated regions (truncating)
non-sign. regions
stability
CCND2
mutated regions (truncating)
non-sign. regions
PPM1D
mutated regions (truncating)
non-sign. regions
STK11
mutated regions (truncating)
non-sign. regions
TP53
mutated regions (truncating)
non-sign. regions
VHL
mutated regions (truncating)
non-sign. regions

Figure S11. Proteomics-guided interpretation of missense and truncating mutations in cancer genes, related to Figure 3.
For each cancer gene, we analyzed structural constraints that aligned with the spatial distribution of its significantly mutated regions. We distinguished significant (yellow: missense; purple: truncating mutations) vs. non-significant (blue) regions within functional domains as well as the rest of the protein (gray). *Top left*: Violin plots show the distribution of structural features (% of max.) in significant (missense; purple: truncating mutation) vs. non-significant parts of functional domains (blue) as well as the rest of each cancer gene (gray), marking their median (horizontal lines), interquartile range (boxes), and 10/90% percentiles (vertical lines). *Top right*: We visualized the 3D protein structures of significant (yellow) and nonsignificant parts of functional domains (blue) and the rest of each gene (gray), along with key ligands related to their function (red). Middle row: We plotted structural features (y-axis, % of max.) against their location in each gene (x-axis). Bottom row: We also visualized the number of mutations (y-axis, z-scores) in each position (x-axis) – to examine the match of structural features (middle row) and the spatial distribution of mutations (bottom row).

For missense mutations (rows 1 and 2), these analyses identified several structural features that differ between significant vs. non-significant regions in functional domains (e.g., amino acids directly interacting with GTP vs. the rest of the GTPase domain), allowing deeper insights into their roles in tumorigenesis. For truncating mutations (rows 3 and 4), these analyses revealed that truncating mutations primarily target structured regions in tumor suppressors (row 4) – likely reflecting constraints related to truncating mutations triggering nonsense-mediated decay. Truncating mutations show the opposite pattern in oncogenes (row 3), where they primarily accumulate in unstructured terminal loops, where they do not trigger nonsense-mediated decay and may remove signals marking them for posttranslational degradation.

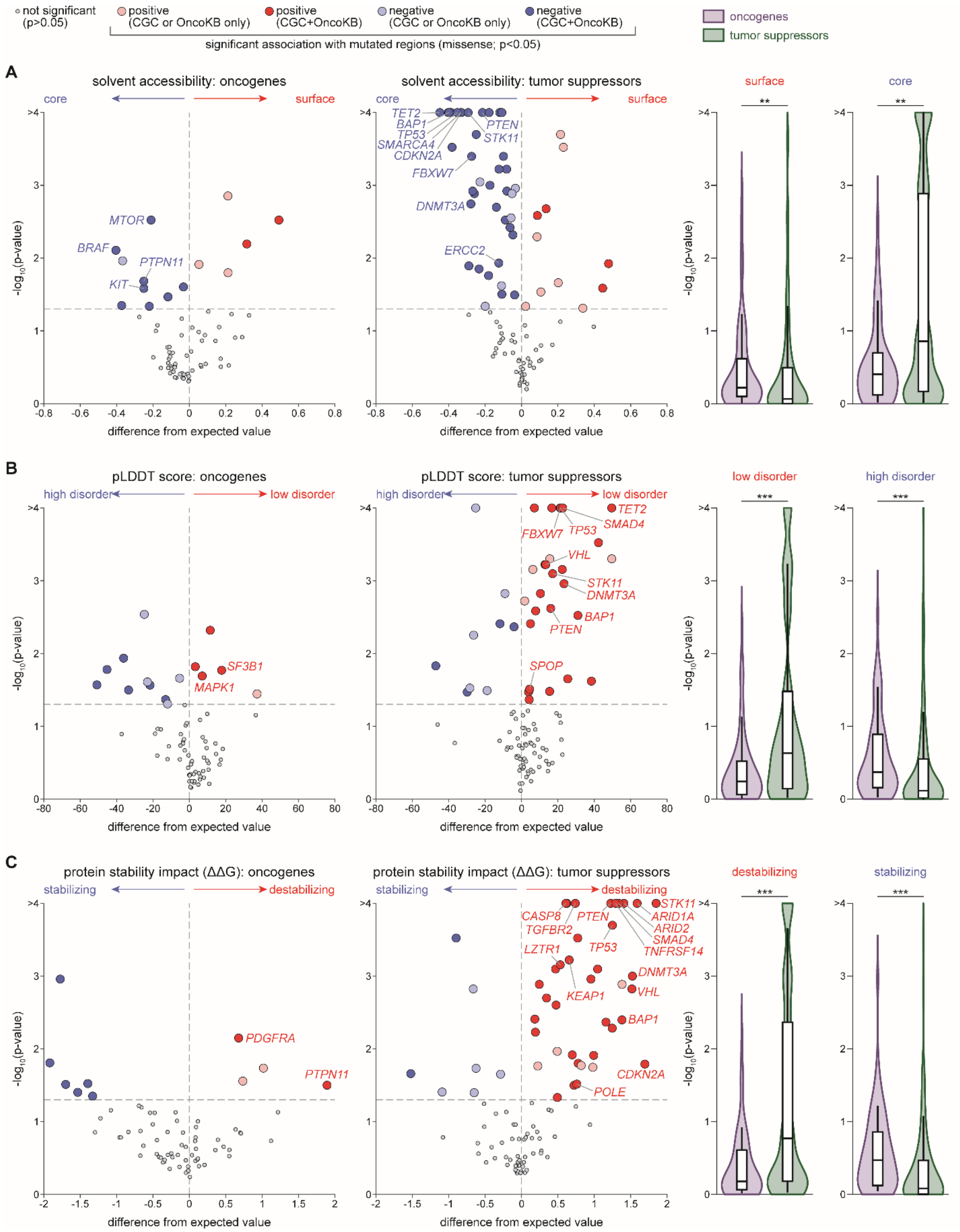

Figure S12. Enrichment of structure-derived metrics in significant regions of missense mutations, related to Figure 4.

(A-C) We evaluated the association between missense mutations within significantly mutated regions and structure-derived metrics, including relative solvent accessibility (A), intrinsically disordered regions inferred from pLDDT score (B), or predicted effects on protein stability (ΔΔG; C) using a random permutation test. Solvent accessible surface area (SASA) was predicted by DSSP and normalized by the theoretical maximum possible SASA for each amino acid, as previously described (Tien et al., PLoS One, 2013), to obtain relative solvent accessibility. pLDDT scores were extracted from PDB files of structures predicted by AlphaFold. The effect on

protein stability was predicted using FoldX. For each gene, we randomly selected regions of the same length as the significantly mutated regions 10,000 times and calculated the weighted average of each structure-derived metric. A one-sided p-value was estimated from the resulting empirical distribution. To reduce spurious associations, we restricted the analysis to the genes that the average normalized mutation count in significant regions was greater than that in non-significant regions. Volcano plots display the $-\log_{10}$(p-value) from the permutation test (y-axis) versus the deviation from expected values estimated by random permutation (x-axis). Genes positively or negatively associated with the structure-derived metrics (p-value < 0.05) are highlighted in red or blue, respectively. Among these, dark or light colors denote oncogenes/tumor suppressors annotated in both the Cancer Gene Census (CGC) and OncoKB (CGC+OncoKB) or in either database alone (CGC or OncoKB only), respectively. Genes annotated in both databases with a weighted average RSA < 0.15, a weighted average pLDDT score > 90, or a weighted average $\Delta\Delta G$ > 2 kcal/mol are labeled. Violin plots show the distribution of $-\log_{10}$(p-value) for each gene, marking the median (horizontal lines), interquartile range (boxes), and 10/90% percentiles (vertical lines) of each distribution. Statistically significant differences between oncogenes and tumor suppressors are denoted with asterisks (**$P < 0.01$, ***$P < 0.001$; two-sided Wilcoxon rank-sum test). For direct comparison with oncogenes, the volcano plot of tumor suppressors for relative solvent accessibility (middle of panel A) is also shown here, although it is presented in Figure 4A.

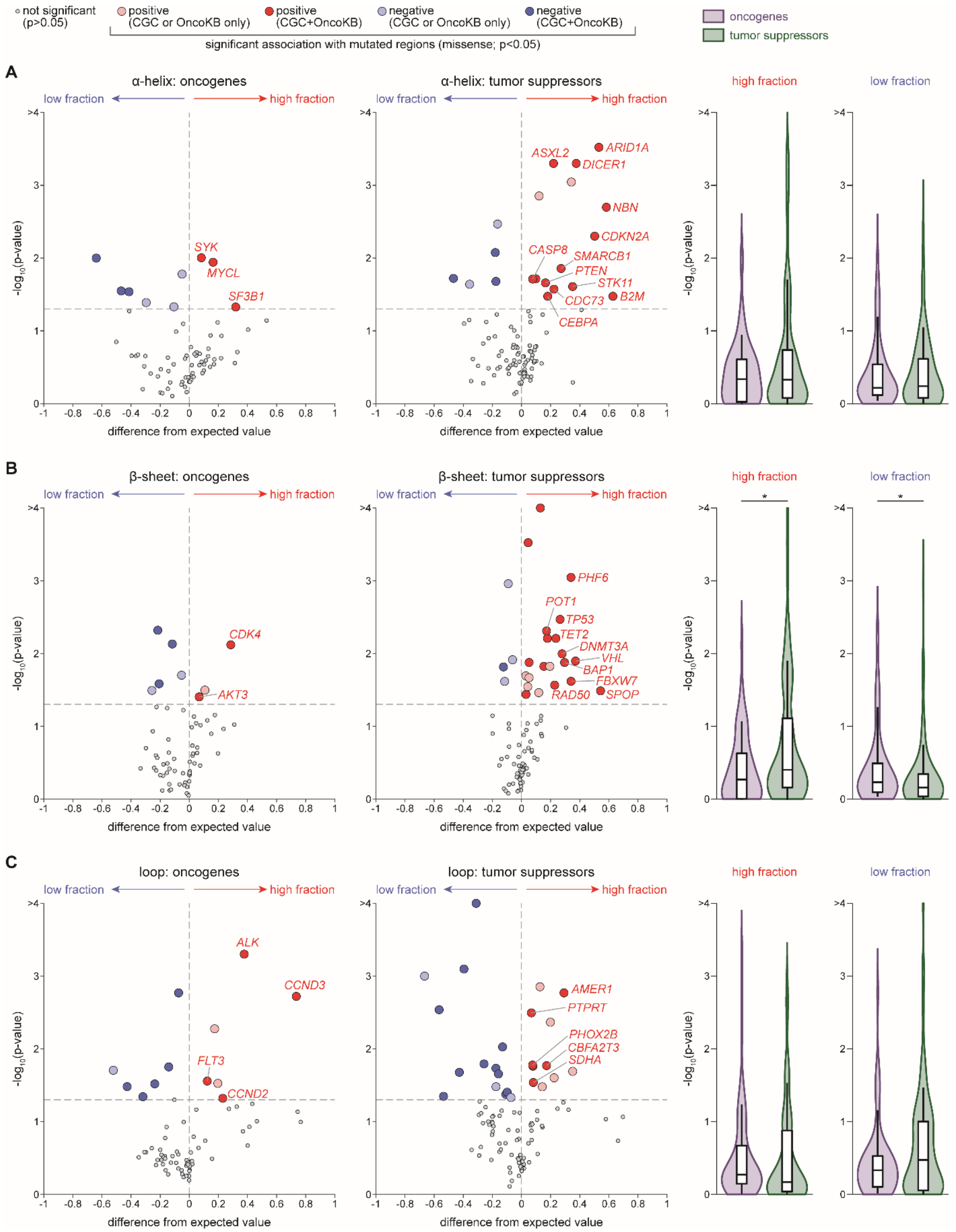

Figure S13. Enrichment of secondary structures in significant regions of missense mutations, related to Figure 4.

(A-C) We quantified the enrichment of missense mutations within significantly mutated regions in α-helix (A), β-sheet (B), or loop (C) secondary structures using a random permutation test. The locations of α-helices, β-sheets, or loops were predicted by DSSP. For each gene, we randomly selected regions of the same length as the significantly mutated regions 10,000 times and calculated the weighted fraction of overlap with secondary structures. A one-sided p-value was estimated from the resulting empirical distribution. To reduce spurious associations, we restricted the analysis to the genes that the average normalized mutation count in significant

regions was greater than that in non-significant regions. Volcano plots display the $-\log_{10}$(p-value) from the permutation test (y-axis) versus the deviation from expected values estimated by random permutation (x-axis). Genes positively or negatively associated with secondary structures (p-value < 0.05) are highlighted in red or blue, respectively. Among these, dark or light colors denote oncogenes/tumor suppressors annotated in both the Cancer Gene Census (CGC) and OncoKB (CGC+OncoKB) or in either database alone (CGC or OncoKB only), respectively. Genes annotated in both databases with a weighted fraction of overlap > 0.25 are labeled. Violin plots show the distribution of $-\log_{10}$(p-value) for each gene, marking the median (horizontal lines), interquartile range (boxes), and 10/90% percentiles (vertical lines) of each distribution. Statistically significant differences between oncogenes and tumor suppressors are denoted with asterisks (*P < 0.05; two-sided Wilcoxon rank-sum test). For direct comparison with oncogenes, volcano plots of tumor suppressors for α-helix and β-sheet (middle of panels A–B) are also shown here, although these plots are presented in Figure 4A.

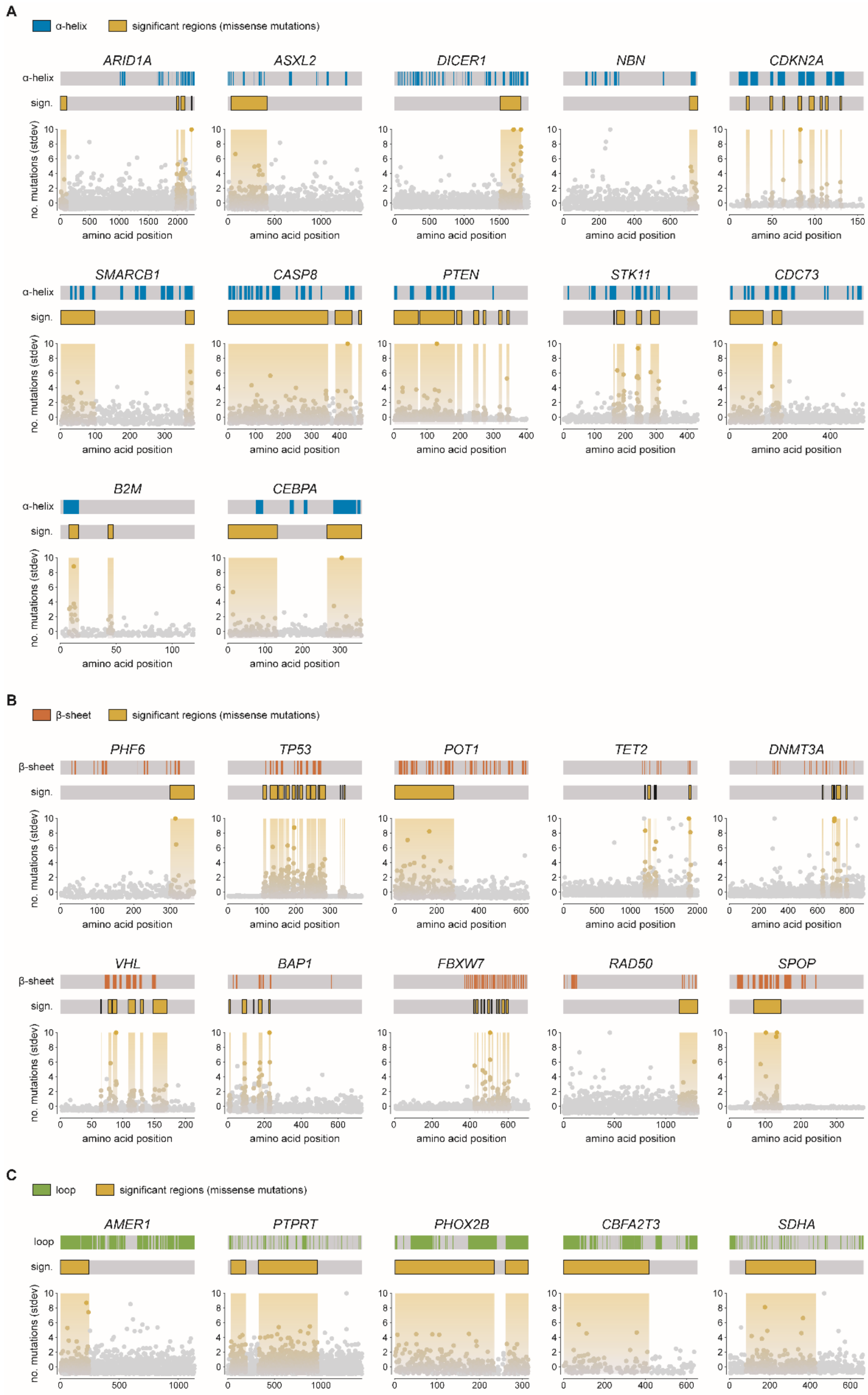

Figure S14. Overlap of secondary structures and significant regions, related to Figure 4.
(A-C) For each cancer gene positively associated with α-helix (A), β-sheet (B), or loop (C) secondary structures (enrichment p-value < 0.05), we plotted the number of missense mutations (y-axis) against their position along the gene (x-axis). To account for the fluctuation in background mutation rates, we normalized mutation counts as standard deviations from the mean (z-scores). At the top of each plot, we annotated the locations of α-helices (blue), β-sheets (orange), or loops (green) as predicted by DSSP.

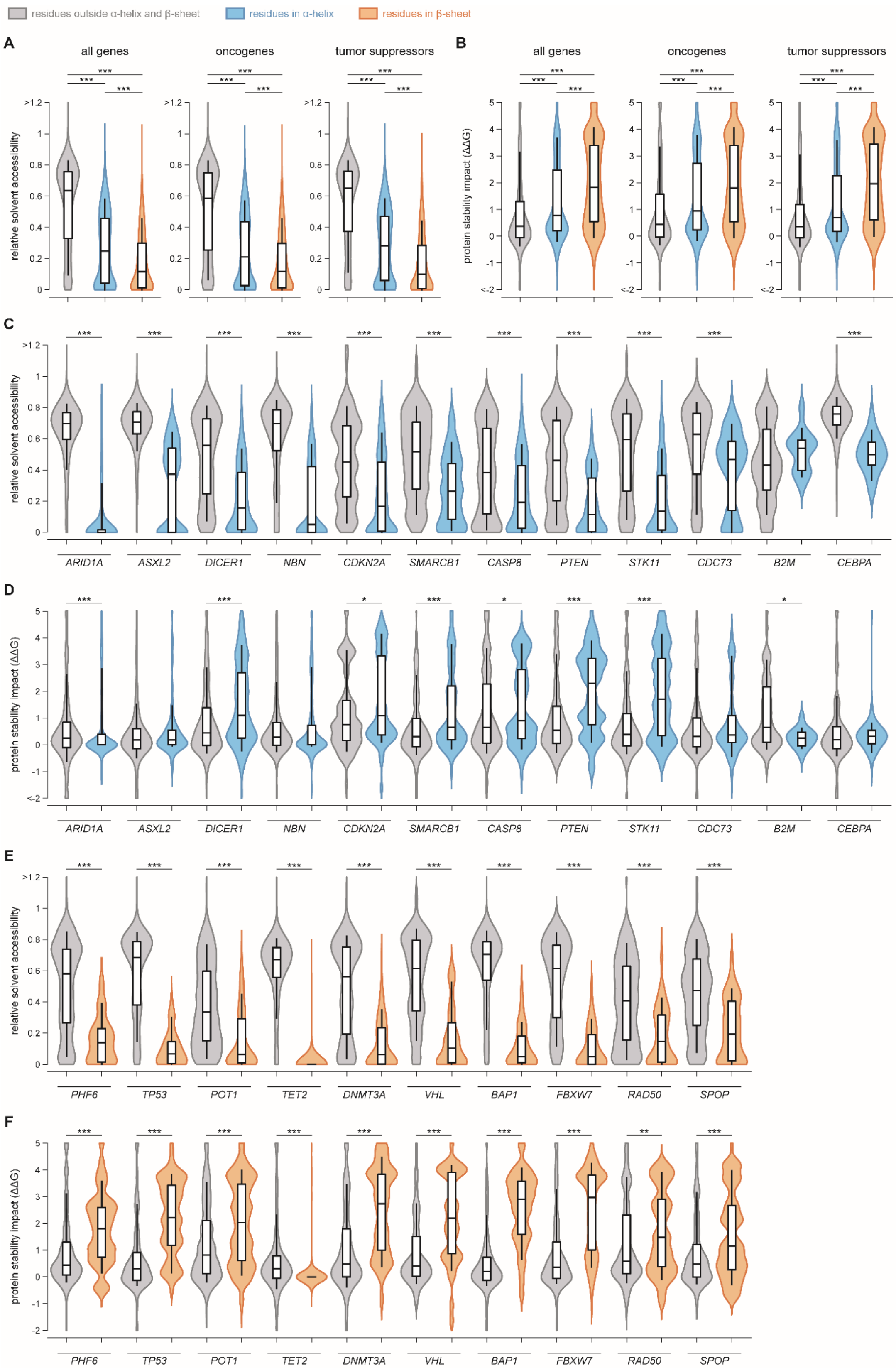

Figure S15. Comparison of solvent accessibility and protein stability impacts across secondary structures, related to Figure 4.

(A-B) We compared relative solvent accessibility (A) and protein stability impacts (ΔΔG; B) among residues located in α-helices (blue), β-sheets (orange), and regions outside α-helices and β-sheets (gray). We included only genes that the average normalized mutation count in significant regions was greater than that in non-significant regions. We classified genes as oncogenes or tumor suppressors based on the Cancer Gene Census and OncoKB databases. Statistically significant differences are denoted with asterisks (***P < 0.001; Kruskal-Wallis test with Tukey's posthoc correction). We annotated the locations of α-helices and β-sheets as predicted by DSSP.

(C-F) For each cancer gene positively associated with α-helices (C-D) or β-sheets (E-F) (enrichment p-value < 0.05), we compared relative solvent accessibility (C, E) and protein stability impacts (ΔΔG; D, F) between residues within α-helices (blue) or β-sheets (orange) and residues outside α-helices and β-sheets (gray). Statistically significant differences are denoted with asterisks (*P < 0.05, **P < 0.01, ***P < 0.001; two-sided Wilcoxon rank-sum test).

Violin plots visualize the distribution of each metric, horizontal lines indicate the median, boxes span the interquartile range, and whiskers extend to the $10/90^{th}$ percentiles of each distribution.

These data suggest that mutations occurring within α-helices or β-sheets often localize to the interior core and residues with greater predicted impacts on protein stability.

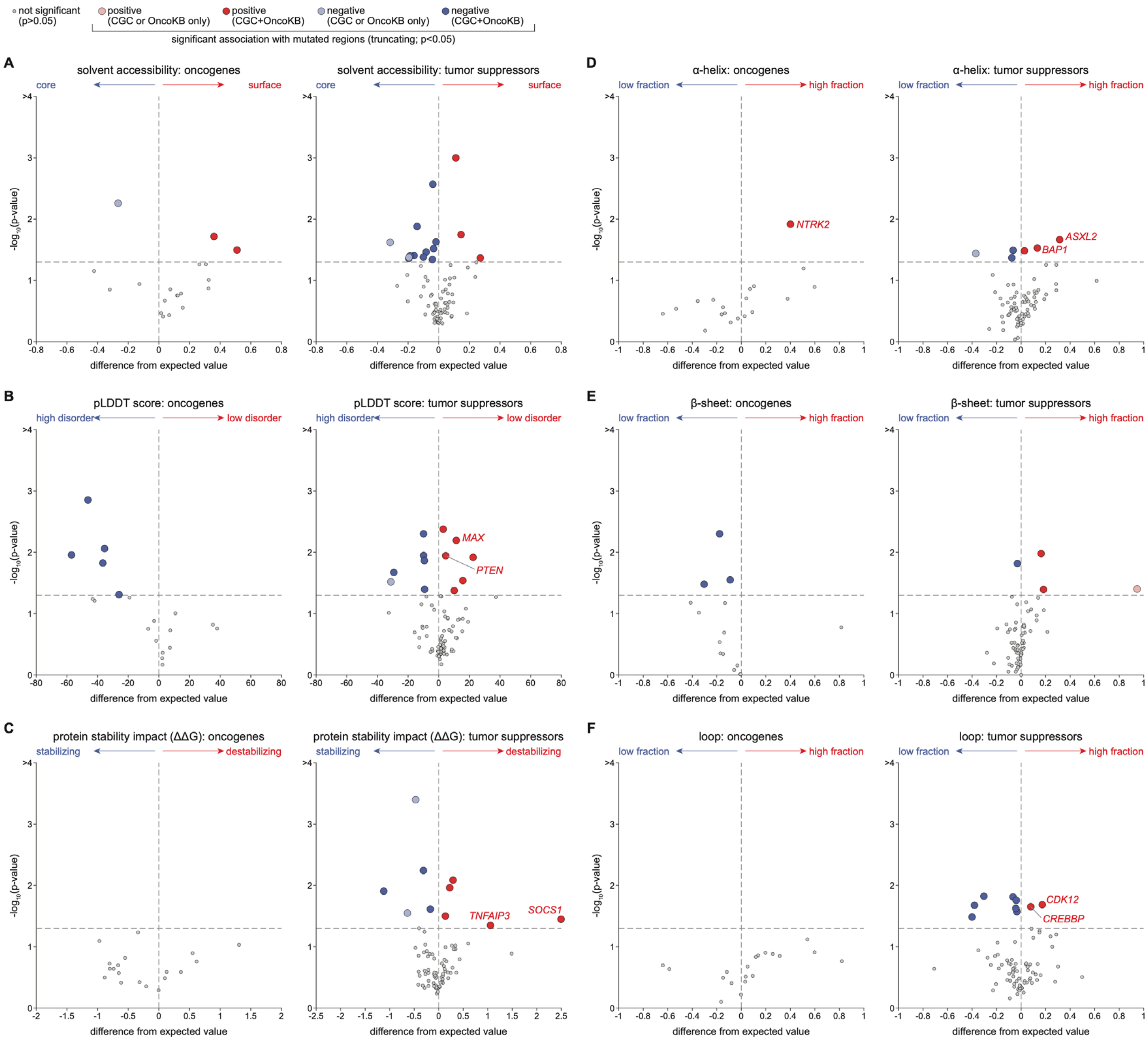

Figure S16. Enrichment of structure-derived metrics and secondary structures in significant regions of truncating mutations, related to Figure 4.

(A-C) We evaluated the association between truncating mutations within significantly mutated regions and structure-derived metrics, including relative solvent accessibility (A), intrinsically disordered regions inferred from pLDDT score (B), or predicted effects on protein stability (ΔΔG; C) using a random permutation test. Solvent accessible surface area (SASA) was predicted by DSSP and normalized by the theoretical maximum possible SASA for each amino acid, as previously described (Tien et al., PLoS One, 2013), to obtain relative solvent accessibility. pLDDT scores were extracted from PDB files of structures predicted by AlphaFold. The effect on protein stability was predicted using FoldX. For each gene, we randomly selected regions of the same length as the significantly mutated regions 10,000 times and calculated the weighted average of each structure-derived metric. A one-sided p-value was estimated from the resulting empirical distribution. To reduce spurious associations, we restricted the analysis to the genes for which the average normalized mutation count in significant regions was greater than that in non-significant regions. Volcano plots display the $-\log_{10}$(p-value) from the permutation test (y-axis) versus the deviation from expected values estimated by random permutation (x-axis). Genes positively or negatively associated with the structure-derived metrics (p-value < 0.05) are highlighted in red or blue, respectively. Among these, dark or light colors denote oncogenes/tumor suppressors annotated in both the Cancer Gene Census (CGC) and OncoKB (CGC+OncoKB) or in either database alone (CGC or OncoKB

only), respectively. Genes annotated in both databases with a weighted average RSA < 0.15, a weighted average pLDDT score > 90, or a weighted average ΔΔG > 2 kcal/mol are labeled.

(D-F) We quantified the enrichment of truncating mutations within significantly mutated regions in α-helix (D), β-sheet (E), or loop (F) secondary structures using a random permutation test. The locations of α-helices, β-sheets, or loops were predicted by DSSP. For each gene, we randomly selected regions of the same length as the significantly mutated regions 10,000 times and calculated the weighted fraction of overlap with secondary structures. A one-sided p-value was estimated from the resulting empirical distribution. To reduce spurious associations, we restricted the analysis to genes for which the average normalized mutation count in significant regions was greater than that in non-significant regions. Volcano plots display the $-\log_{10}$(p-value) from the permutation test (y-axis) versus the deviation from expected values estimated by random permutation (x-axis). Genes positively or negatively associated with secondary structures (p-value < 0.05) are highlighted in red or blue, respectively. Among these, dark or light colors denote oncogenes/tumor suppressors annotated in both the Cancer Gene Census (CGC) and OncoKB (CGC+OncoKB) or in either database alone (CGC or OncoKB only), respectively. Genes annotated in both databases with a weighted fraction of overlap > 0.25 are labeled.

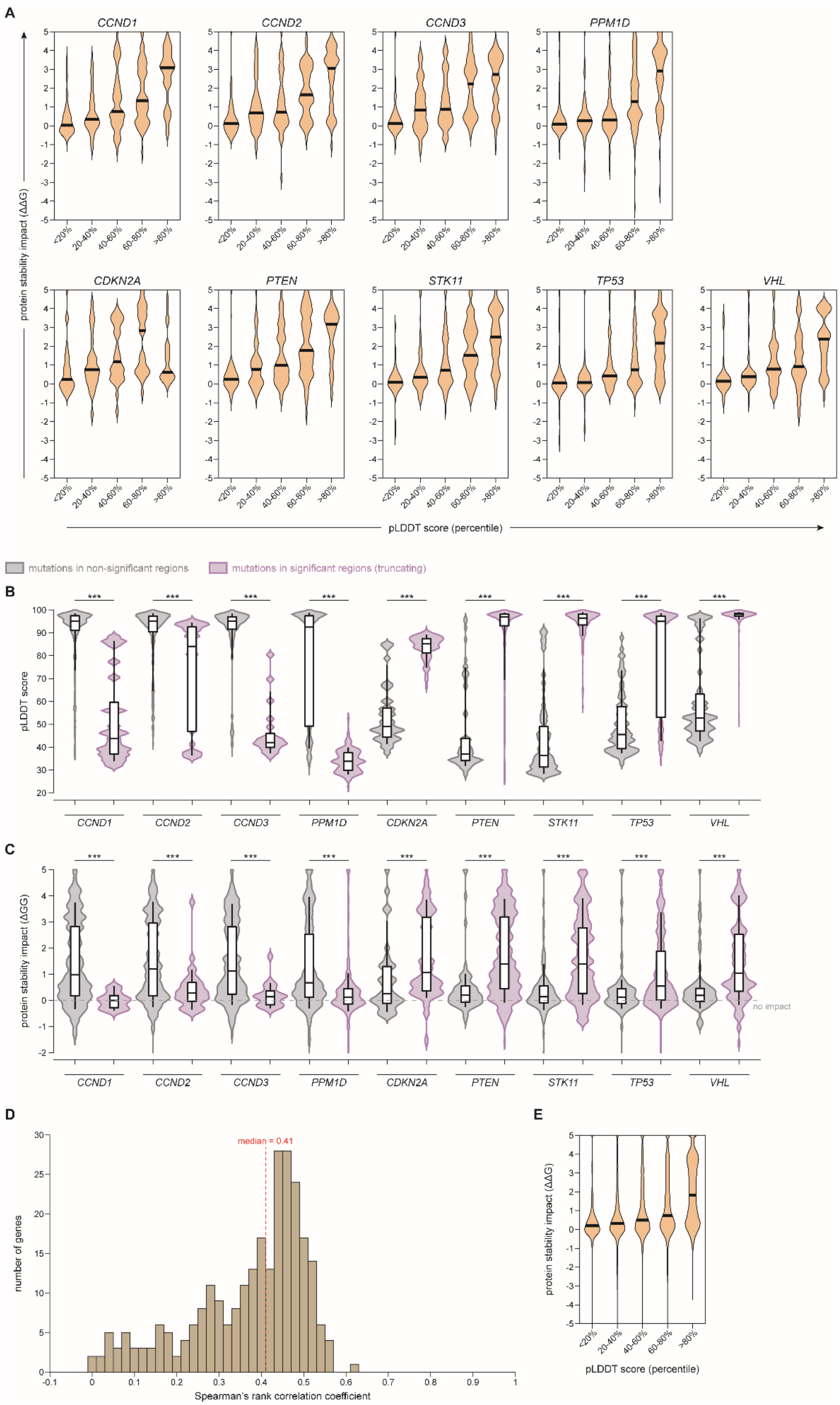
A
CCND1
CCND2
CCND3
PPM1D
CDKN2A
PTEN
STK11
TP53
VHL
protein stability impact (ΔΔG)
<20%
20-40%
40-60%
60-80%
>80%
pLDDT score (percentile)
mutations in non-significant regions
mutations in significant regions (truncating)
B
pLDDT score
***
C
protein stability impact (ΔΔG)
no impact
D
median = 0.41
number of genes
Spearman's rank correlation coefficient
E
protein stability impact (ΔΔG)
pLDDT score (percentile)

Figure S17. Correlation between pLDDT scores and different metrics of protein stability in significant and non-significant regions, related to Figure 4.
(A) We evaluated whether AlphaFold pLDDT score correlates with predicted effects on protein stability in genes with truncating mutations depleted in their N- and C-terminal loops. We grouped residues based on their pLDDT score (20% percentile bins, x-axis) and visualized the distribution of their protein stability impact (ΔΔG) as a violin plot (y-axis). Horizontal lines within each violin plot indicate the median of each distribution.

(B-C) We compared pLDDT score (A) and protein stability impacts (ΔΔG; B) between residues in significantly mutated regions (purple) vs. non-significant regions (gray) of truncating mutations. Violin plots visualize the distribution of pLDDT scores and ΔΔG values, horizontal lines indicate the median, boxes span the interquartile range, and whiskers extend to the 10/90th percentiles of each distribution. Dashed horizontal lines mark the baseline of no change in protein stability. Statistically significant differences are denoted with asterisks (***$P < 0.001$; two-sided Wilcoxon rank-sum test).

(D) We evaluated whether AlphaFold pLDDT score generally correlates with predicted effects on protein stability. Histograms show the distribution of Spearman's rank correlation coefficients between pLDDT scores and protein stability impact across residues from 267 cancer genes.

(E) We grouped residues based on their pLDDT score (20% percentile bins) and visualized the distribution of their protein stability impact (ΔΔG) as a violin plot (y-axis). Horizontal lines within each violin plot indicate the median of each distribution. Overall, the distributions demonstrate a positive trend, suggesting that higher pLDDT scores are generally associated with greater effects on protein stability.

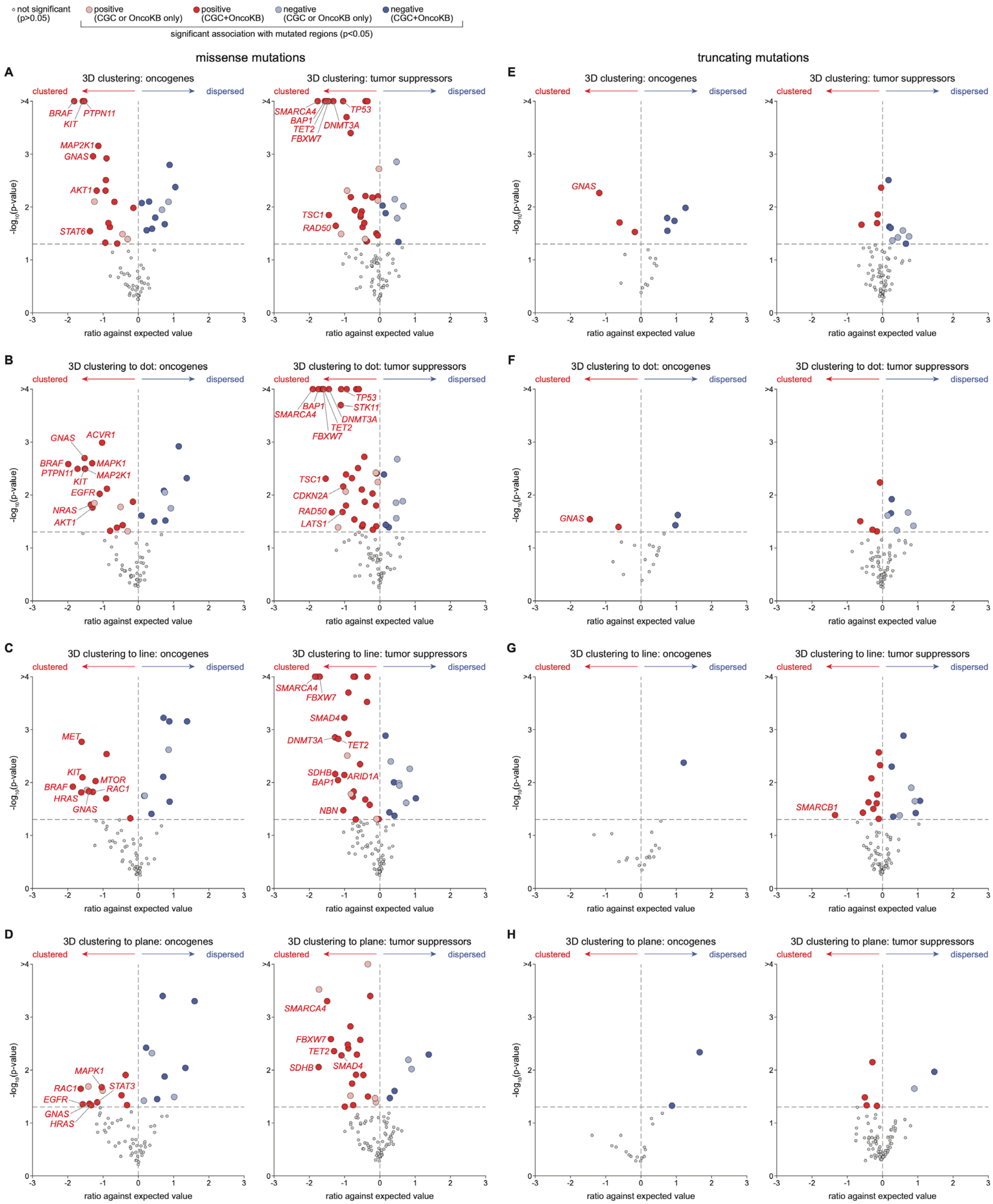

Figure S18. Association of 3D geometry of mutation patterns with significant regions, related to Figure 4. We evaluated whether missense mutations (A-D) and truncating mutations (E-H) within significantly mutated regions exhibit spatial clustering in three-dimensional (3D) space (A, E) and whether they form pocket-like (B, F), line-like (C, G), or plane-like (D, H) clusters using a random permutation test.

For each gene, we randomly selected regions of the same length as the significantly mutated regions 10,000 times and calculated the weighted average of pairwise distances among residues within significant regions (missense mutations: A, truncating mutations: E), as well as distances to a virtual dot, line, or plane (missense mutations: B-D, truncating mutations: F-H). A one-sided p-value was estimated from the resulting empirical distribution. To reduce spurious associations, we restricted the analysis to genes for which the average normalized mutation count in significant regions was greater than that in non-significant regions.

Volcano plots display the $-\log_{10}$(p-value) from the permutation test (y-axis) versus the deviation from expected values estimated by random permutation (x-axis). Genes positively or negatively associated with 3D clustering metrics (p-value < 0.05) are highlighted in red or blue, respectively. Among these, dark or light colors denote oncogenes/tumor suppressors annotated in both the Cancer Gene Census (CGC) and OncoKB (CGC+OncoKB) or in either database alone (CGC or OncoKB only), respectively. Genes annotated in both databases with a ratio against the expected value < 0.5 are labeled.

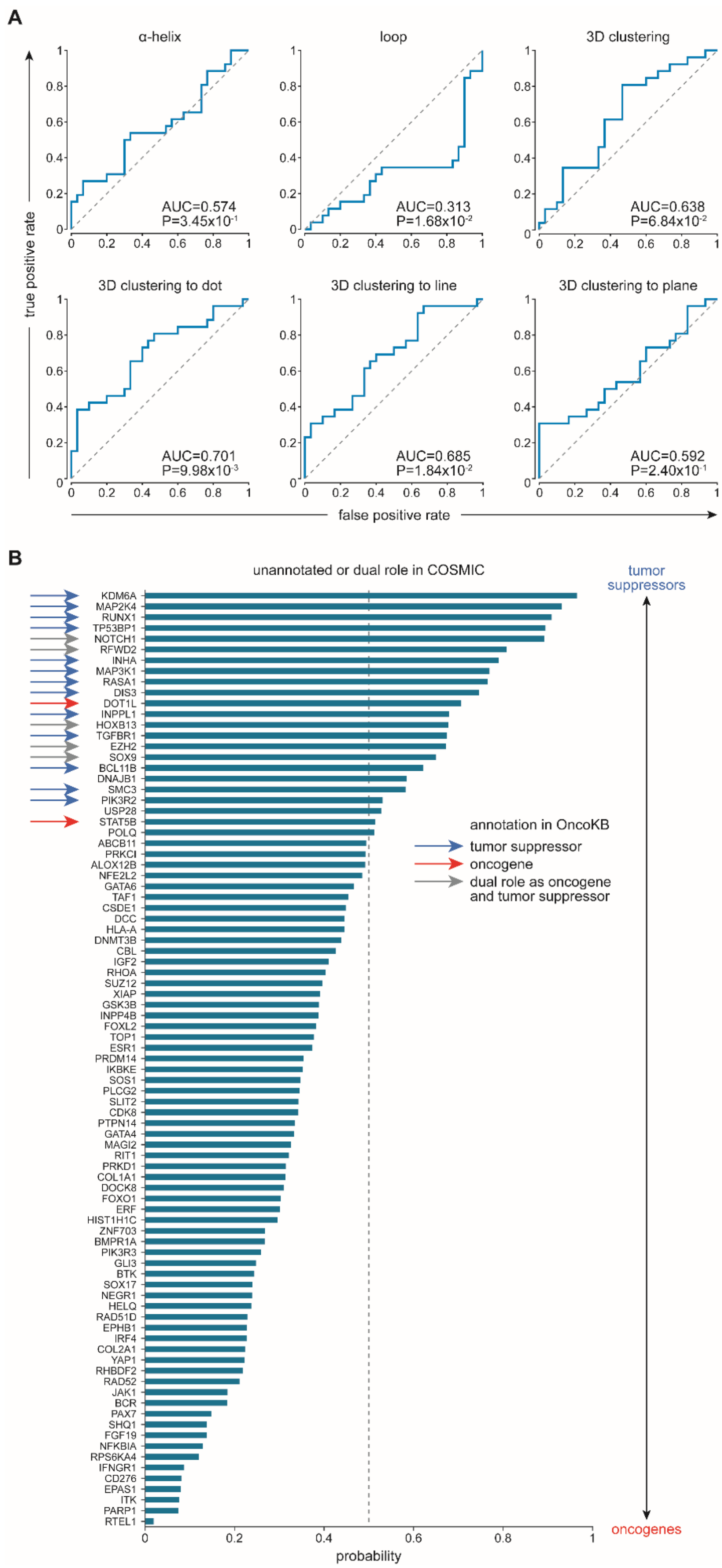

Figure S19. Distinction of oncogenes vs. tumor suppressors based on structural features, related to Figure 4.

(A) We quantified the enrichment of tumor suppressors among cancer genes showing associations between significantly mutated regions and individual structural features, including a high fraction of secondary structure

elements (α-helix and loop) and strong three-dimensional clustering. Cancer genes were ranked using the significance of association derived from permutation tests (See STAR Methods). The area under the curve (AUC) is shown. Statistically significant differences (P) in the significance of association between oncogenes and tumor suppressors were assessed using two-sided Wilcoxon rank-sum tests.

(B) Using annotations from OncoKB, we evaluated the LASSO regression model on genes not annotated as oncogenes or tumor suppressors in the COSMIC Cancer Gene Census, as well as genes annotated as both categories, provided they had a ratio of normalized mutation counts between significant and non-significant regions > 1. We plotted the predicted probability of being a tumor suppressor as a bar plot. Genes annotated as oncogenes, tumor suppressors, and both categories are indicated by red, blue, and gray arrows, respectively.

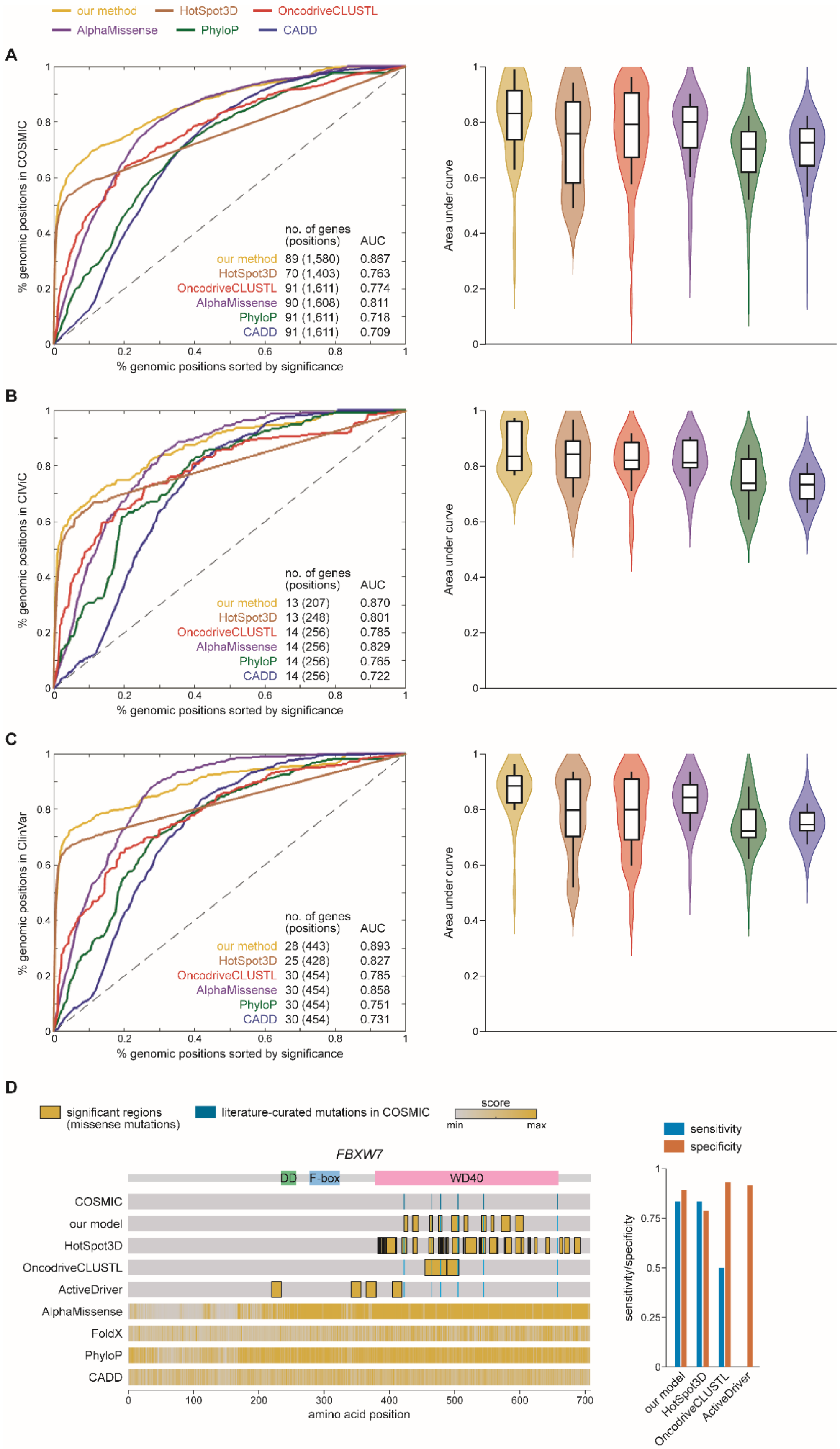
our method
HotSpot3D
OncodriveCLUSTL
AlphaMissense
PhyloP
CADD
A
% genomic positions in COSMIC
% genomic positions sorted by significance
no. of genes (positions)
AUC
our method 89 (1,580) 0.867
HotSpot3D 70 (1,403) 0.763
OncodriveCLUSTL 91 (1,611) 0.774
AlphaMissense 90 (1,608) 0.811
PhyloP 91 (1,611) 0.718
CADD 91 (1,611) 0.709
Area under curve
B
% genomic positions in CIViC
our method 13 (207) 0.870
HotSpot3D 13 (248) 0.801
OncodriveCLUSTL 14 (256) 0.785
AlphaMissense 14 (256) 0.829
PhyloP 14 (256) 0.765
CADD 14 (256) 0.722
C
% genomic positions in ClinVar
our method 28 (443) 0.893
HotSpot3D 25 (428) 0.827
OncodriveCLUSTL 30 (454) 0.785
AlphaMissense 30 (454) 0.858
PhyloP 30 (454) 0.751
CADD 30 (454) 0.731
D
significant regions (missense mutations)
literature-curated mutations in COSMIC
score
min
max
FBXW7
DD
F-box
WD40
COSMIC
our model
HotSpot3D
OncodriveCLUSTL
ActiveDriver
AlphaMissense
FoldX
PhyloP
CADD
amino acid position
sensitivity
specificity
sensitivity/specificity
our model
HotSpot3D
OncodriveCLUSTL
ActiveDriver

Figure S20. Oncogenic mutations accumulate in significant regions and exhibit distinct enrichment patterns across driver discovery methods and pathogenicity scores, related to Figure 5.
(A-C) We quantified the enrichment of genomic positions harboring oncogenic mutations in significant vs. non-significant regions of each cancer gene (ROC curves), using COSMIC (A), CIViC (B), or ClinVar (C) as a reference of literature-curated oncogenic mutations. Genomic positions were ranked using significance scores generated by our method and by three existing methods (HosSpot3D, OncodriveCLUSTL, and ActiveDriver). We further sorted genomic positions based on predicted pathogenicity (AlphaMissense; purple), effects on protein structure (FoldX; pink), evolutionary conservation (PhyloP; green), and deleteriousness (CADD; blue). Deviations of the ROC curves toward the top left indicate stronger enrichment of oncogenic mutations. The number of genes and genomic positions annotated in the oncogenic mutation databases (COSMIC, CIViC, and ClinVar) included in the ROC curves, as well as the area under curve (AUC) for each method, are provided. Violin plots (insets) show the distribution of the area under the ROC curve for each gene, marking the median (horizontal lines), interquartile range (boxes), and 10/90% percentiles (vertical lines) of each distribution.

(D) We compared the overlap between amino acid positions harboring oncogenic mutations curated in COSMIC and significantly mutated regions identified by our method and existing methods (HotSpot3D, OncodriveCLUSTL, and ActiveDriver) in *FBXW7*. The color bar represents the gradient of indicated scores (AlphaMissense, FoldX, PhyloP, and CADD) assigned to individual amino acid positions. Sensitivity and specificity for capturing oncogenic mutations by each method are shown as a bar graph on the right.

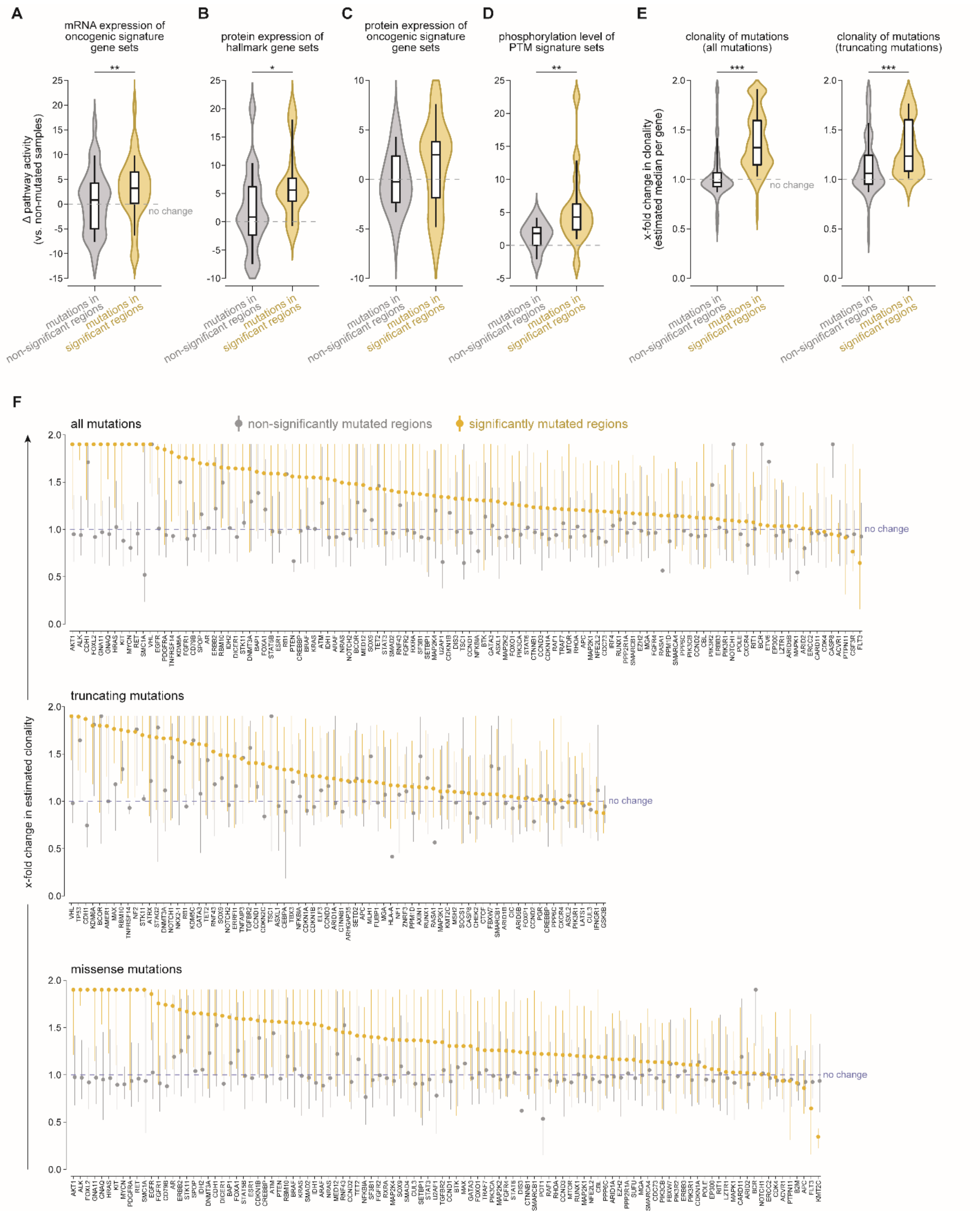

Figure S21. Downstream signaling changes and clonality of mutations in significant vs. non-significant regions, related to Figure 6.

(A-D) To cross-validate the impact of mutations on downstream signaling activity, we evaluated pathway activity signatures on mRNA and protein expression, and phosphorylation level data.

(A) We compared the signaling activity of mutated vs. non-mutated samples (y-axis) using oncogenic gene signatures in MSigDB on mRNA expression data. Differences between mutated vs. non-mutated samples were quantified based on signatures related to their gene activity (gene set enrichment analysis), yielding a normalized enrichment score for each mutated gene and region.

(B-D) To validate these differences on an independent datatype, we used proteomics and phosphoproteomics data in CPTAC. We applied hallmark (B) and oncogenic (C) signatures to proteomics data (MSigDB) and post-translational modification (PTM) signatures to phospho-proteomics data (PTMSigDB) (D). We quantified differences between mutated vs. non-mutated samples using a gene set enrichment analysis.

(E) We estimated the relative clonality of each mutation by comparing the number of mutated vs. non-mutated reads, relative to the read counts of other mutations in the same genome.

Violin plots show the distribution of these enrichment metrics for mutations in significant (orange) vs. non-significant (gray) regions. Horizontal lines mark the median, boxes indicate the interquartile range, and whiskers extend to the 10/90th percentiles. Dashed lines represent the baseline of each metric based on non-mutated samples. Statistically significant differences are indicated with asterisks (*$P < 0.05$, **$P < 0.01$; two-sided Wilcoxon rank-sum test).

(F) To confirm that these differences were consistent across genes, we plotted the relative clonality separately for each gene. Dots represent the median relative clonality of mutations in significant (orange) vs. non-significant (gray) regions for each gene, and vertical lines span their interquartile range. Dashed horizontal lines marks the normalized baseline. Plots are shown for all mutations (top), truncating mutations only (middle), and missense mutations only (bottom).

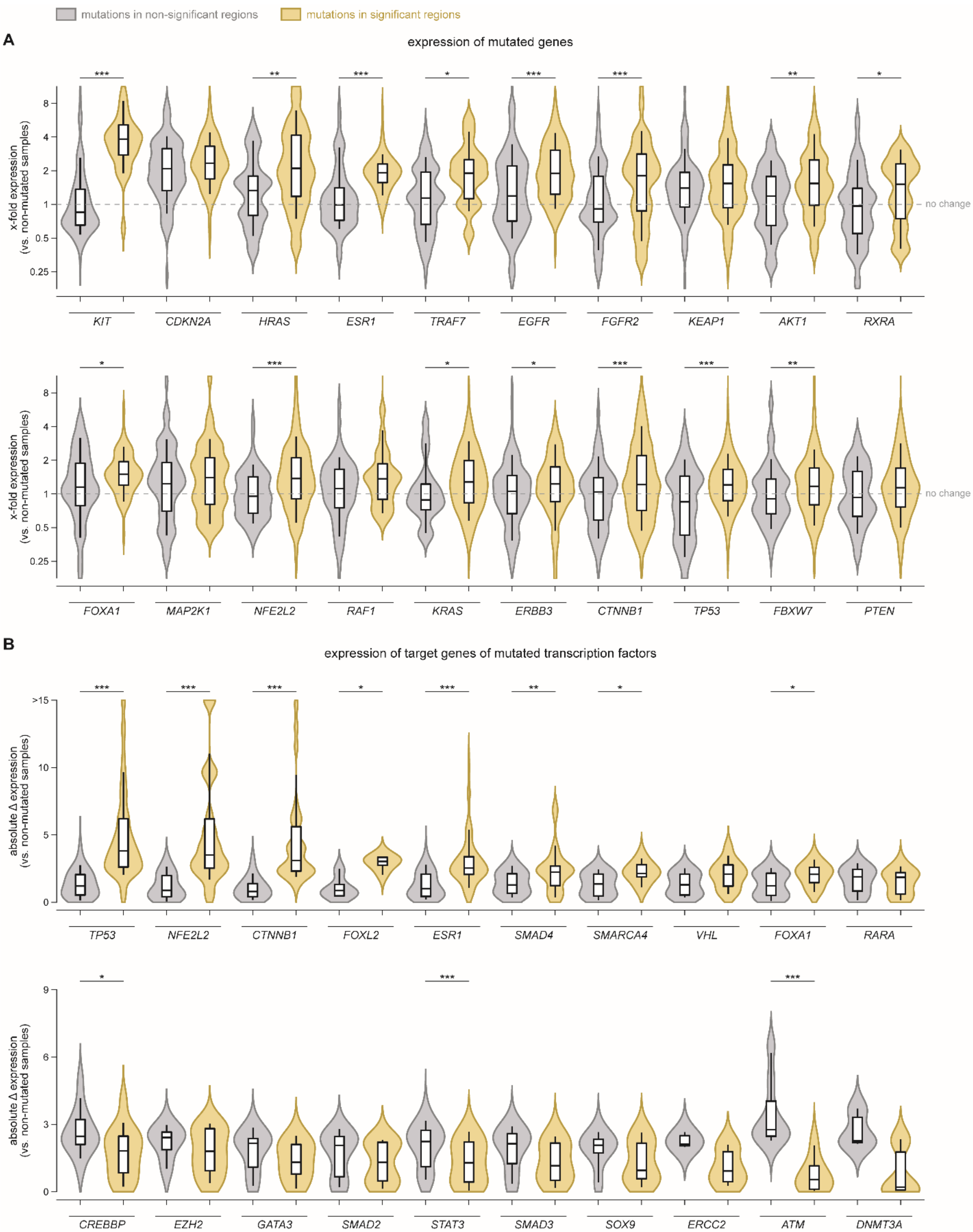

Figure S22. Consistency of expression changes across cancer genes, related to Figure 6.
We combined multiple cancer genes to compare the effect of mutations in significant vs. non-significant regions (see Figure 6). To assess the robustness of these metrics, we plotted our analyses separately for individual genes.

(A) We compared the expression of mutated genes with their level in non-mutated samples (ratio of expression in the mutated sample to the median expression in non-mutated samples). (B) We evaluated the transcriptional activity of mutated transcription factors relative to their activity in non-mutated samples (rank-sum z-statistic based on the expression of their target genes).

Violin plots visualize the distribution of these metrics for mutations in significant (orange) vs. nonsignificant (gray) regions, separately for 20 representative genes. Horizontal lines indicate their median, boxes span the interquartile range, and vertical lines extend to the 10/90th percentiles. Dashed horizontal lines mark the baseline of each metric based on non-mutated samples. Statistically significant differences are annotated with asterisks (*P < 0.05, **P < 0.01, ***P < 0.001; two-sided Wilcoxon rank-sum tests).

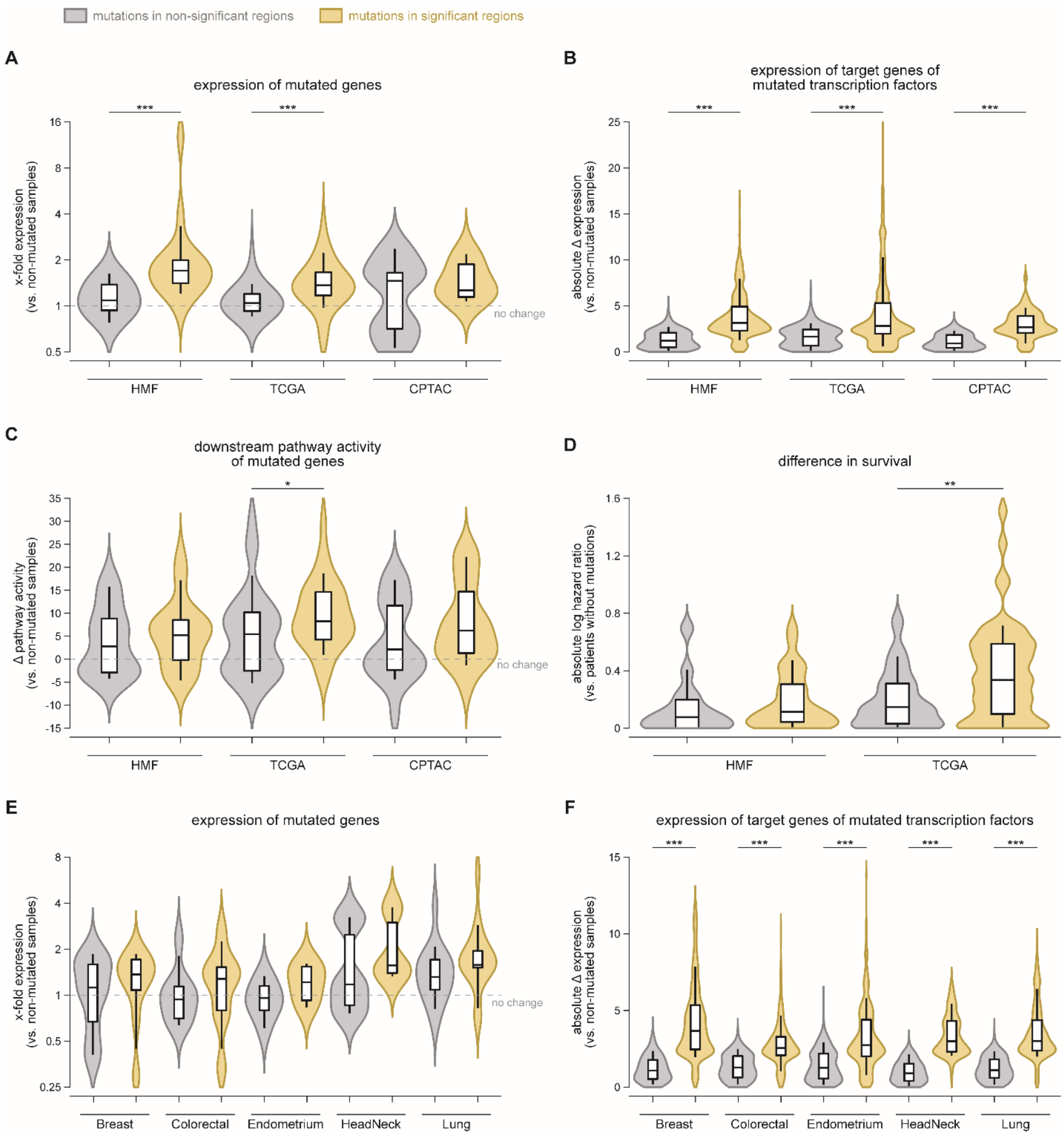

Figure S23. Consistency of mutation metrics across independent datasets and cancer types, related to Figure 6.

We combined three datasets encompassing multiple cancer types to compare the effect of mutations in significant vs. non-significant regions in cancer genes (see Figure 6).

(A-D) We plotted the following metrics separately for each dataset to confirm their consistency across datasets: (A) The expression of mutated genes relative to that in non-mutated samples (ratio of median expression in the mutated sample to its median expression in non-mutated samples). (B) The activity of mutated transcription factors vs. their activity in non-mutated samples (rank-sum z-statistics of target gene expression in mutated vs. non-mutated samples). (C) Downstream signaling activity of mutated genes vs. those in non-mutated samples (gene set enrichment analysis of signatures associated with gene activity). (D) Differences in overall survival of

mutated vs. nonmutated patients, adjusting for tumor type and other patient-specific variables (absolute hazard ratio). CPTAC was excluded from this analysis due to insufficient survival data.

(E-F) We plotted the following metrics separately for individual cancer types to confirm their consistency across cancer types: (E) The expression of mutated genes relative to their level in nonmutated samples (ratio of expression in mutated sample to median expression of the same gene in non-mutated samples). (F) The activity of mutated transcription factors relative to their activity in non-mutated samples (rank-sum z-statistic based on their target gene expression).

Violin plots show the distribution of these metrics in significant (orange) vs. non-significant (gray) regions of cancer genes, separately for independent datasets (A-D) or individual cancer types (E-F). Horizontal lines represent the median, boxes span the interquartile range, and whiskers extend to the 10/90th percentiles. Dashed horizontal lines mark the baseline of each metric based on nonmutated samples. Statistically significant differences are indicated by asterisks (*P < 0.05, **P < 0.01, ***P < 0.001; Wilcoxon rank-sum test).

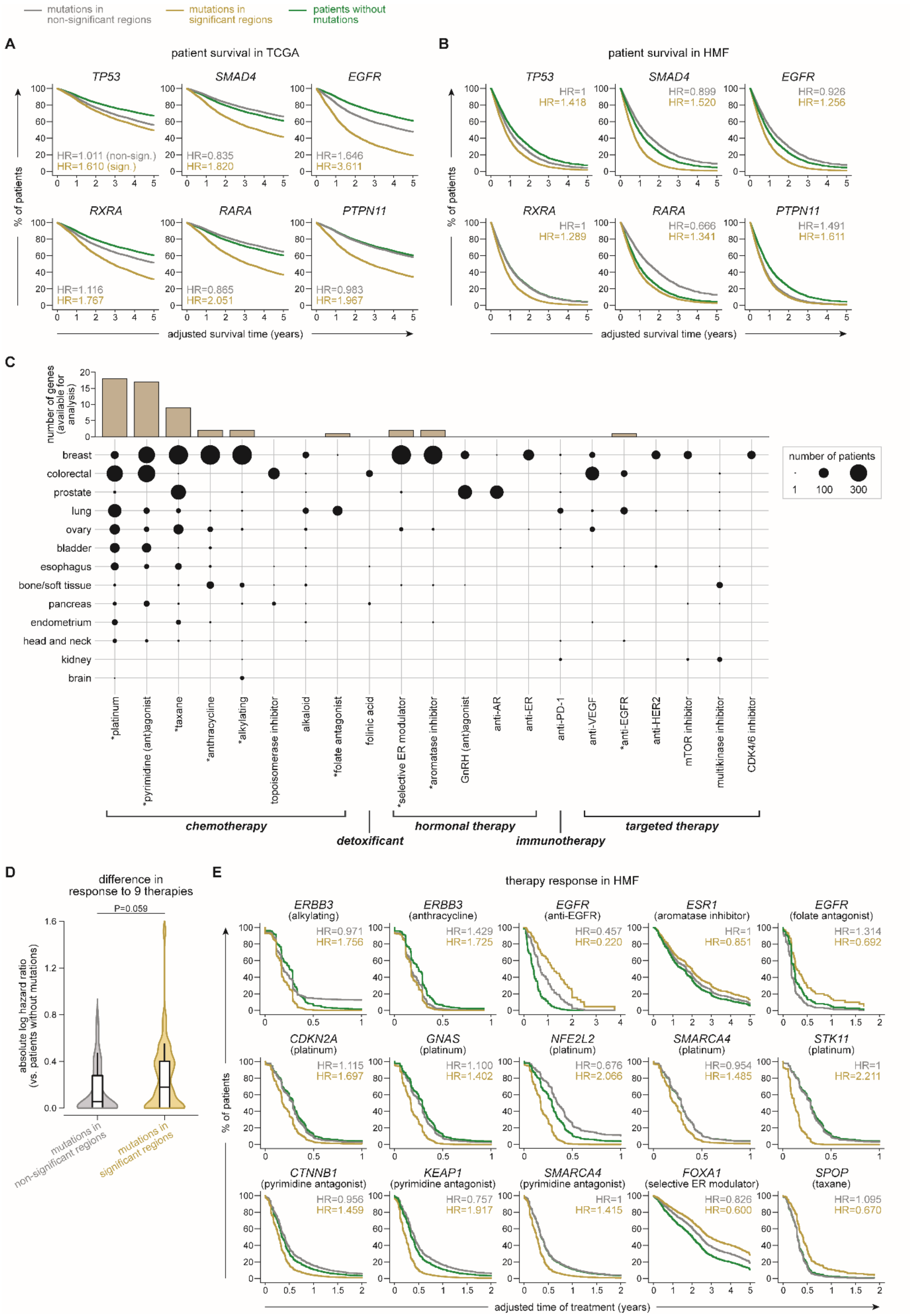
mutations in non-significant regions
mutations in significant regions
patients without mutations
A
patient survival in TCGA
B
patient survival in HMF
% of patients
adjusted survival time (years)
C
number of genes (available for analysis)
number of patients
D
difference in response to 9 therapies
P=0.059
absolute log hazard ratio (vs. patients without mutations)
E
therapy response in HMF
adjusted time of treatment (years)
chemotherapy
detoxificant
hormonal therapy
immunotherapy
targeted therapy

Figure S24. Clinical outcomes associated with mutations in significant regions, related to Figure 6.
(A-B) Kaplan-Meier curves illustrate differences in overall survival among patients in TCGA (A) and HMF (B) with mutations in significant (orange) vs. non-significant (gray) regions (exemplarily shown for *TP53*, *SMAD4*, *EGFR*, *RXRA*, *RARA*, and *PTPN11*). As a reference, we plotted the survival of patients without mutations in these genes (green). Hazard ratios from Cox regression model for non-significant (gray) and significant (orange) regions are shown. Survival times were adjusted for differences between cancer types. see STAR Methods.

(C) To visualize the therapeutic landscape of the HMF dataset, we collected pre-biopsy treatment information for patients in the HMF dataset across 21 therapies (y-axis) and 13 cancer types (x-axis). We plotted the number of patients receiving each therapy for each cancer type. We excluded therapies for which treatment duration data were available for fewer than 50 patients. Therapies were grouped into five categories (chemotherapy, detoxificant, hormonal therapy, immunotherapy, and targeted therapy) and sorted within each category by the total number of treated patients across all cancer types. Cancer types were ordered by the total number of patients across all therapies. Dot size is proportional to the number of patients (see legend). The bar graph (top) shows the number of genes with annotated treatment data for at least five mutations in both significant and non-significant regions for each therapy. To minimize spurious associations, only these genes were included in subsequent analyses. Of the 21 therapies, we finally used nine therapies (indicated by asterisks) for which at least one gene was available for analysis.

(D) We compared the time until the treatment was discontinued (e.g., due to inefficacy or disease progression) between patients harboring mutations in significant (orange) vs. non-significant (gray) regions in cancer genes across nine therapies. We estimated hazard ratios comparing patients with and without mutations using Cox regression model (see STAR Methods). Violin plots show the distribution of absolute log hazard ratios for mutations in significant (orange) vs. non-significant (gray) regions. Horizontal lines indicate their median, boxes span interquartile ranges, and whiskers extend to their 10/90% percentiles. Statistical difference was evaluated by two-sided Wilcoxon rank-sum test ($P = 0.059$).

(E) Kaplan-Meier curves illustrate differences in the treatment response between patients with mutations in significant (orange) vs. non-significant (gray) regions. As a reference, we plotted the survival of patients without mutations in these genes (green). Hazard ratios from Cox regression model for non-significant (gray) and significant (orange) regions are shown. Gene-therapy pairs with larger absolute log hazard ratios for significant regions are shown across nine individual therapies. Survival times were adjusted for differences across cancer types. see STAR Methods.

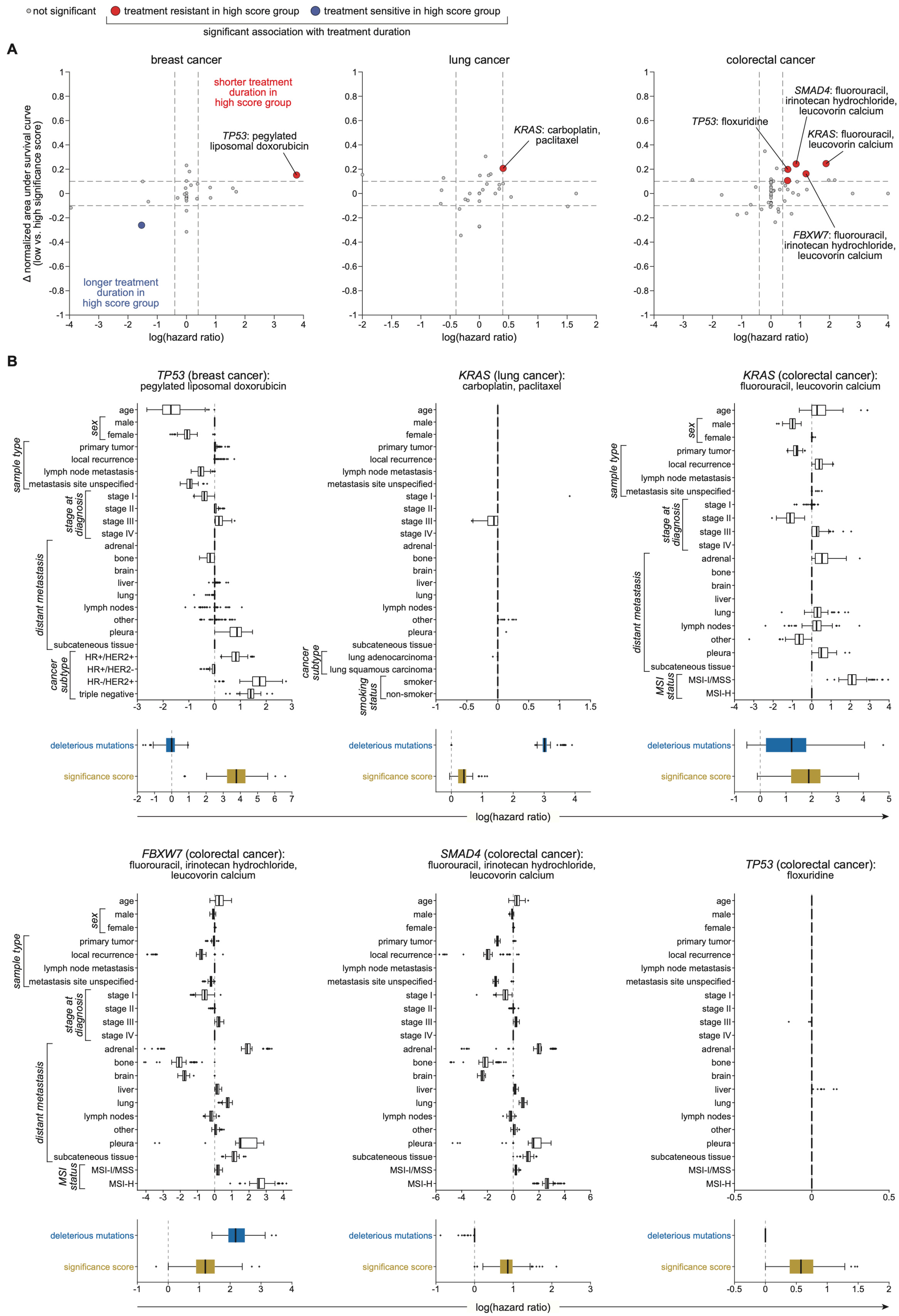

not significant
treatment resistant in high score group
treatment sensitive in high score group
significant association with treatment duration
A
breast cancer
lung cancer
colorectal cancer
shorter treatment duration in high score group
longer treatment duration in high score group
TP53: pegylated liposomal doxorubicin
KRAS: carboplatin, paclitaxel
SMAD4: fluorouracil, irinotecan hydrochloride, leucovorin calcium
TP53: floxuridine
KRAS: fluorouracil, leucovorin calcium
FBXW7: fluorouracil, irinotecan hydrochloride, leucovorin calcium
Δ normalized area under survival curve (low vs. high significance score)
log(hazard ratio)
B
TP53 (breast cancer): pegylated liposomal doxorubicin
KRAS (lung cancer): carboplatin, paclitaxel
KRAS (colorectal cancer): fluorouracil, leucovorin calcium
FBXW7 (colorectal cancer): fluorouracil, irinotecan hydrochloride, leucovorin calcium
SMAD4 (colorectal cancer): fluorouracil, irinotecan hydrochloride, leucovorin calcium
TP53 (colorectal cancer): floxuridine
age
sex
male
female
sample type
primary tumor
local recurrence
lymph node metastasis
metastasis site unspecified
stage at diagnosis
stage I
stage II
stage III
stage IV
distant metastasis
adrenal
bone
brain
liver
lung
lymph nodes
other
pleura
subcateneous tissue
cancer subtype
HR+/HER2+
HR+/HER2-
HR-/HER2+
triple negative
lung adenocarcinoma
lung squamous carcinoma
smoking status
smoker
non-smoker
MSI status
MSI-I/MSS
MSI-H
deleterious mutations
significance score
log(hazard ratio)

Figure S25. Associations between therapy response and mutations in significant regions of cancer genes in breast cancer, related to Figure 6.

We compared progression-free survival among patients with mutations in significant regions, stratified by high vs. low significance, in breast, lung, and colorectal cancer within the GENIE Biopharma Collaborative cohorts. High- and low-significance groups were defined using the 50th percentile of significance scores assigned to mutations in significant regions by our framework for each gene. To evaluate associations between mutations and progression-free survival while adjusting for covariates, we first built a baseline Cox regression model to predict progression-free survival using clinical covariates, including, sex, sample type, stage at diagnosis, and distant metastasis status across all cancer types, with additional covariates included where available: cancer subtype for breast cancer, cancer subtype and smoking status for lung cancer, and microsatellite instability (MSI) status for colorectal cancer. We then incorporated deleterious mutations (e.g., nonsense and frameshift mutations) and significance scores for missense mutations into this baseline model and assessed whether they improved predictive performance. To prevent overfitting, we applied LASSO regularization with 10-fold cross-validation and 100 subsampling iterations. To reduce spurious associations, we included only treatments with data from at least 50 patients and gene-treatment pairs with annotated progression-free survival data for at least 10 mutations in significant regions, including at least 3 mutations in both high- and low-significance groups. We also required at least half of the events to be non-censored in each mutation-score group. In addition, we restricted the analysis to the genes for which the average normalized mutation count in significant regions was greater than that in non-significant regions.

(A) Scatter plots display the log(hazard ratio) from the regression model for significance scores (x-axis) versus the difference in normalized area under the Kaplan-Meier curves (i.e., restricted mean survival time) between low and high significance groups (y-axis) for the indicated cancer types. Gene-treatment pairs (dots) with significant positive or negative associations [|log(hazard ratio)|>0.4, |∆normalized AUC (low - high)|>0.1, and |∆normalized AUC (no mutation - high)|>0.05] are highlighted in red or blue, respectively.

(B) Box plots show the distribution of hazard ratios related to progression-free survival for each predictor (x-axis, log scale) across subsampling iterations for the indicated genes, therapies, and cancer types. Boxes span the interquartile range, whiskers extend to 1.5x the interquartile range, and vertical lines indicate the median. HR, hormone receptor (ESR1); HER2, human epidermal growth factor receptor 2 (ERBB2).

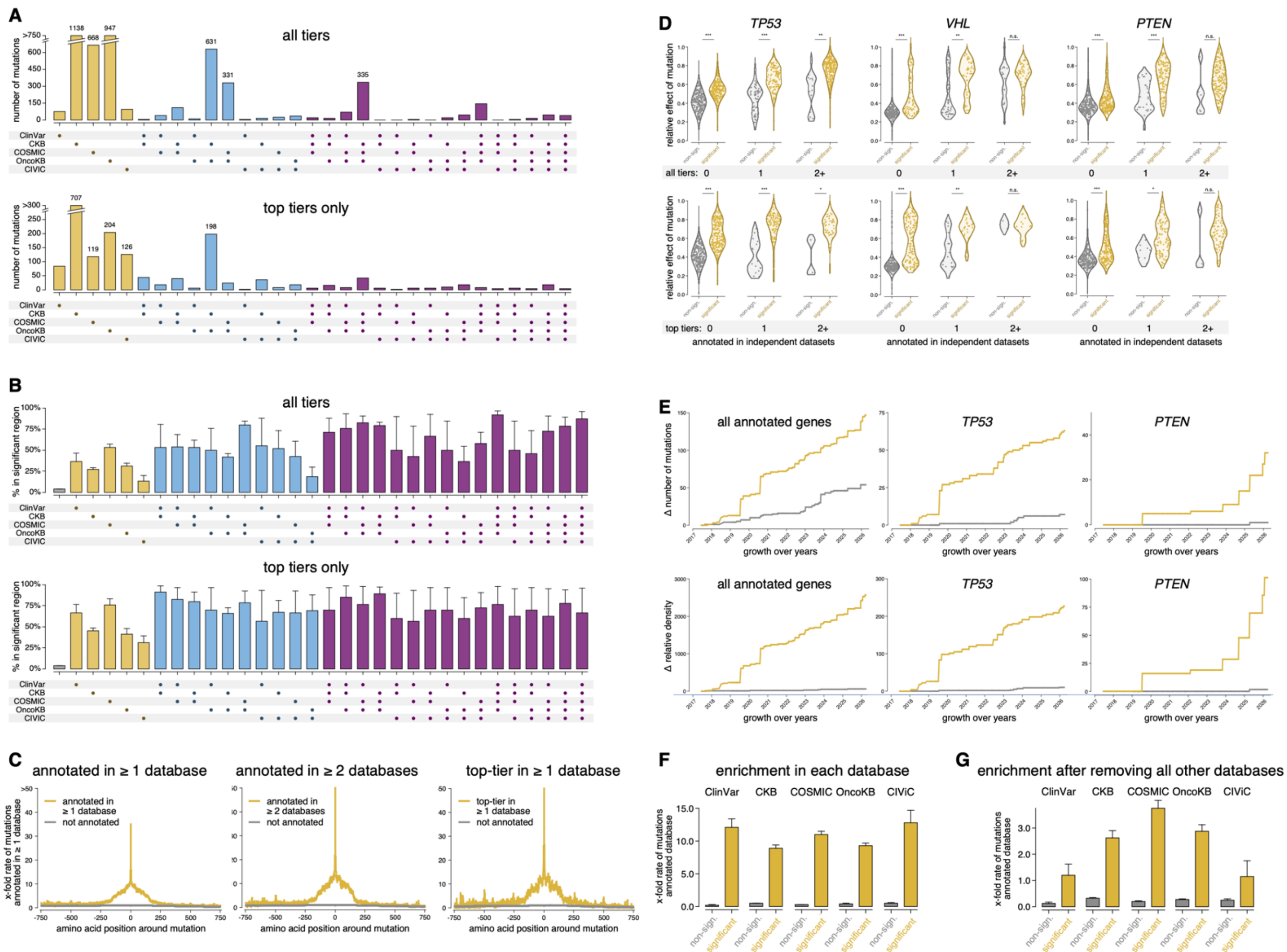

Figure S26. Clinical annotations are inconsistent across databases and significant regions provide context for interpreting singly annotated mutations, related to Figure 6.

(A) UpSet plots show the overlap across five databases commonly used for clinical annotation of oncogenic mutations (ClinVar, CKB, COSMIC, OncoKB, CIViC). For a fair comparison, only genes annotated by ≥3 independent databases were included, because not all databases curate the same set of genes. Overlap is shown for all annotations (top) and top-tier annotations (bottom).

(B) Bar graphs show the fraction of mutations falling within significant regions for each overlap category (orange, blue, purple) as well as for mutations not annotated in any database (gray) – for all annotations (top) and top-tier annotations (bottom). Whiskers indicate 95% confidence intervals estimated using the Wilson method.

These data indicate that most annotations are supported by a single database only, indicating substantial discordance across resources and uncertainty in clinical interpretation. Mutations supported by multiple databases were more likely to fall within significant regions, suggesting that significant regions may help prioritize singly annotated mutations for further clinical review.

(C) For mutations annotated as oncogenic in ≥1 database (left), in ≥2 databases (middle), or as top-tier in ≥1 database (right), we computed the odds that nearby mutations were also annotated as oncogenic in at least one database (odds ratio relative to the baseline annotation rate; y-axis). We plotted this enrichment as a function of amino acid position relative to the reference mutation (x-axis). As a control, we computed the same odds ratios around mutations not annotated in any database (gray). Oncogenic mutations cluster locally within protein sequences – a biologically meaningful signal leveraged by our hidden Markov model to support the interpretation of mutations with limited database support.

(D) Violin plots compare normalized effect scores (y-axis) of mutations in significant (yellow) vs. non-significant (gray) regions in *TP53*, *VHL*, and *PTEN* (% of maximum effect in mutagenesis screens). Mutations are stratified by support in 0, 1, or ≥2 independent databases (left to right), based on top-tier annotations (top) and all annotations (bottom)). Differences between significant vs. non-significant regions were prominent for singly annotated mutations, suggesting that significant regions can help prioritize oncogenic mutations with limited database support for further clinical review.

(E) Increase of ClinVar annotations over time (x-axis) for mutations in significant (yellow) vs. non-significant (gray) regions – shown as absolute number of annotations (top) or density normalized by region size (bottom) for all annotated genes (left), *TP53* (middle), and *PTEN* (right). To facilitate comparability, once a mutation appeared in a ClinVar release, it was retained in all subsequent versions. Although significant regions span only a subset of each gene, new annotations accumulated in significant regions at a higher density, suggesting that mutations in significant regions are more likely to be annotated as oncogenic in later ClinVar releases.

(F) Bar graphs show enrichment (odds ratio relative to baseline annotation frequency) of annotated mutations in significant (yellow) vs. non-significant (gray) regions in each database (ClinVar, CKB, COSMIC, OncoKB, CIViC). Bars represent 95% confidence intervals estimated using the Wilson method.

(G) Enrichment persisted after excluding mutations annotated in other databases, indicating that the signal was not driven solely by well-established mutations, but remained for singly annotated mutations in each database.

Together, these results support the biological relevance of singly annotated mutations in significant vs. non-significant regions. They suggest that significant regions may help prioritize oncogenic mutations that have limited support in existing annotation resources.
